# The *WTX/AMER1 *gene family: evolution, signature and function

**DOI:** 10.1186/1471-2148-10-280

**Published:** 2010-09-15

**Authors:** Agnès Boutet, Glenda Comai, Andreas Schedl

**Affiliations:** 1 INSERM, U636, F-06108 Nice, France; 2 Université de Nice-Sophia Antipolis, Laboratoire de génétique du développement Normal et Pathologique, F-06108 Nice, France

## Abstract

**Background:**

*WTX *is a novel gene mutated in a proportion of Wilms' tumors and in patients suffering from sclerosing bone dysplasia. On the molecular level WTX has been shown to act as an antagonist of canonical *Wnt/β-catenin *signaling in fish and mammals thus linking it to an essential pathway involved in normal development and cancer formation. Interestingly, WTX seems to also localize to an intranuclear component called paraspeckles. In spite of the growing interest of molecular biologists in *WTX*, little is known about its paralogs and its phylogenetic history.

**Results:**

Using the amino-acid sequence of *WTX/AMER1 *as a tool for the assignment of orthology and paralogy, we here identify two novel proteins, *AMER2 *and *AMER3*, as "*WTX*" related. This *Amer *gene family is present in all currently available vertebrate genome sequences, but not invertebrate genomes and is characterized by six conserved blocks of sequences. The phylogenetic analysis suggests that the *protoAmer *gene originated early in the vertebrate lineage and was then duplicated due to whole genome duplications (WGD) giving rise to the three different *Amer *genes.

**Conclusion:**

Our study represents the first phylogenetic analysis of *Amer *genes and reveals a new vertebrate specific gene family that is likely to have played an important role in the evolution of this subphylum. Divergent and conserved molecular functions of *Wtx/Amer1*, *Amer2 *and *Amer3 *are discussed.

## Background

Early 2007, a search for genes deleted in Wilms' tumors, a pediatric solid tumor of the kidney led to the identification of the X-linked gene *WTX *(also called AMER1) [1]. Using large-scale interactome mapping a second independent study demonstrated that WTX induces degradation of β-catenin via the proteasome system, thus identifying this gene as an important modulator of this crucial signaling pathway [2]. Wtx/Amer1 also physically interacts with APC [3], a tumor suppressor gene involved in colorectal cancer [4]. In addition, on the cellular level WTX localizes to subnuclear domains that have been identified as paraspeckles [5]. Recent analysis suggests that WTX may also play an important function during normal development: expression analysis demonstrated a dynamic expression pattern throughout embryogenesis [6] and mutations have been identified in patients suffering from a range of developmental defects including osteopathia striata congenita with cranial sclerosis (OSCS) and cardiac anomalies [7]. To better characterize the functional and structural properties of the *WTX/AMER1 *gene it is essential to understand its molecular evolution and its phylogenetic history. Duplications are common events during evolution and are one of the main driving forces for the emergence of new genes that can lead to the appearance of new gene families. The aim of the present study was to identify potential new members of the "*WTX/AMER*" family, characterize their phylogenetic relationships and analyze their evolutionary history.

## Results and Discussion

### Wtx/Amer1 is the founding member of a novel vertebrate gene family

**Figure 1:**
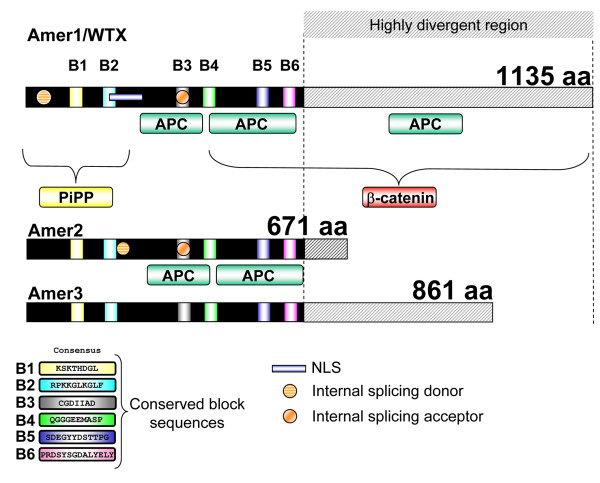
**Primary structure and specific conserved sequence blocks of Amer proteins**. Overall structure of human WTX/AMER1, AMER2 and AMER3 showing the relative position of the conserved blocks (B1 to B6), the APC interacting domains, the nuclear localization signal (NLS), the internal splicing sites (donor and acceptor), the phospholipid binding activity and the β-catenin binding region. Note that B2 partially overlaps with the NLS in human WTX/AMER1. In contrast, only the splicing acceptor is located in a conserved domain (B3). The conserved consensus sequences of domain B1 to B6 are depicted in Figure SM1 B (additional file 1).

**Figure 2:**
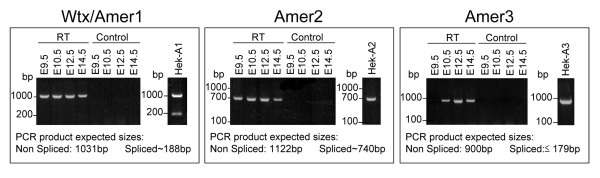
**Alternative splicing of *Amer *transcripts**. RT-PCR of the N-terminus of *Amer *transcripts performed from E9.5 to E14.5 mouse embryos and HEK293T cells transfected with *Wtx/Amer1 *(HEK-A1), *Amer2 *(HEK-A2) or *Amer3 *(HEK-A3). Two RNA products are detectable by RT-PCR after transfection of HEK293T cells with mouse *Wtx/Amer1 *as previously reported [5] (right gel in the Wtx/Amer1 box). By contrast, a single band corresponding to the full-length transcript is detected in mouse embryos suggesting that regulation of splicing may be cell type dependent (left gel). For *Amer2*, only one transcript is detected endogenously in mouse embryos and after transfection of HEK293T cells. Nucleotide sequence analysis revealed a 357-bp (119 amino acids) deletion and indicated that *Amer2 *mRNA is produced by alternative splicing in the 5' end of exon 1. For *Amer3*, a single band corresponding to the full-length transcript is detected either in mouse embryos or after transfection of HEK293T cells. Results have been obtained with three different primer pairs for each *Amer *gene. RT: RNA samples incubated with reverse transcriptase; control: RNA samples incubated without reverse transcriptase as a DNA contamination control.

Using the WTX/AMER1 sequence as a bait for protein-protein comparisons in the human genome, *FAM123A *(*AMER2*) and *FAM123C *(*AMER3*) were identified as two genes that share several domains of significant sequence identity with *WTX/AMER1 *and are located in chromosomes 13 and 2 respectively. *Amer2 *and *Amer3 *were also present in mouse mapping to chromosomes 14 and 1 respectively. Alignment of the mouse and human sequences highlighted the presence of six highly conserved blocks that we named B1 to B6 (Figure 1 and additional file 1, Figure SM1). The mouse and human AMER proteins are encoded by a single exon although 5' untranslated sequences map to additional exons (additional file 1, Figure SM1). In the case of WTX/AMER1 additional splicing within the coding exon can result in alternative protein variants [7]. These spliced forms are detected after transient transfection of mouse Wtx/Amer1 in human cells, but not in mouse embryos from E9.5 to E14.5 dpc (Figure 2). Sequencing of RT-PCR products from the embryonic samples indicated the presence of a shorter transcript than expected for Amer2 (Figure 2). This result showed that splicing leads to an in-frame deletion of amino acids within the N-terminal region of Amer2. The existence of two variants for human AMER2 had already been reported by Grohmann et al. (2007) [3] after *in silico *analysis. Alignment of the spliced form of Amer2 with the full-length transcript revealed a splice site donor with an AG-GT core sequence which is universally found at the exon/intron junction. Sequence analysis localized this splice site donor just after the conserved domain B2 (see Figure 1). By contrast, Amer3 does not seem to undergo splicing, as we detected only one long transcript by RT-PCR both in mouse embryos and in transiently transfected cells with mouse Amer3 (Figure 2).

To further investigate the evolutionary origin and phylogenetic distribution of the *Amer *genes, we extended the *in silico *searches to other available genomic databases. We could identify orthologs of *Wtx/Amer1*, *Amer2 *and *Amer3 *in primates (i.e. *Pan troglodyte*), and all other studied mammals (i.e. *Canis familiaris*), including the most basal therian groups, xenarthra (i.e. *Dasypus novemcinctus*), marsupials (i.e. *Monodelphis domestica*) and monotremes (i.e. *Ornithorhynchus anatinus*). The three *Amer *genes were also present in other tetrapod species such as birds (i.e. *Gallus gallus*), amphibians (i.e. *Xenopus tropicalis*) and in actinopterygians (i.e. the teleost fish *Danio rerio*) (for a complete list of orthologs see additional file 2; Table S1). We were unable to find any ortholog in cyclostome genomes such as the lamprey (*Petromyzon marinus)*, but this is probably due to the fact that this genome has not yet been completely sequenced and assembled.

In stark contrast to their presence in apparently all vertebrates, no *Amer *genes could be identified in the genomes of model invertebrates including flies or nematodes and other protostomes such as mollusks (*Lottia gigantea*) and annelids (*Capitella capitata*). A similar situation already exists for other Wnt signaling components such as the *Dkk *family, which was believed to be vertebrate specific [8]. More recently, however, *Dkk *genes have been described in the diploblastic hydra and sea anemone [9]. It has, therefore, been postulated that gene families are missing in model protostomes due to the fast evolutionary rates and rampant gene loss in *Drosophila *and *Caenorhabditis elegans *genomes [10]. To test whether a similar situation may exist for the *Amer *gene family, we searched for *Amer *orthologs in a wide range of metazoans, from the most basal (cnidarians and placozoans) to the vertebrates' closest relatives. No hits in any of the analyzed genomes could be detected, not even in non-vertebrate chordates (i.e. *Branchiostoma floridae*, *Ciona intestinalis*) and other non-vertebrate deuterostomes such as sea urchin (*Strongylocentrotus purpuratus*) despite the low occurrence of gene loss in some of these species [11]. Although we cannot rule out the possibility that *Amer*-related genes are present in some of these species, but have diverged beyond recognition, our data strongly suggest that this gene family is vertebrate specific. While the six conserved blocks of sequences found in the human and mouse proteins are present in all the vertebrate orthologs (Figure 1 and additional file 1, Figure SM1), PROSITE analysis did not identify protein domains of known function (additional file 1, Figure SM2). Taken together, our data support the common origin of the three genes, which seemed to have arisen at the time of the origin of vertebrates.

### Phylogenetic relationship of *Wtx/Amer1*, *Amer2 *and *Amer3*

**Figure 3:**
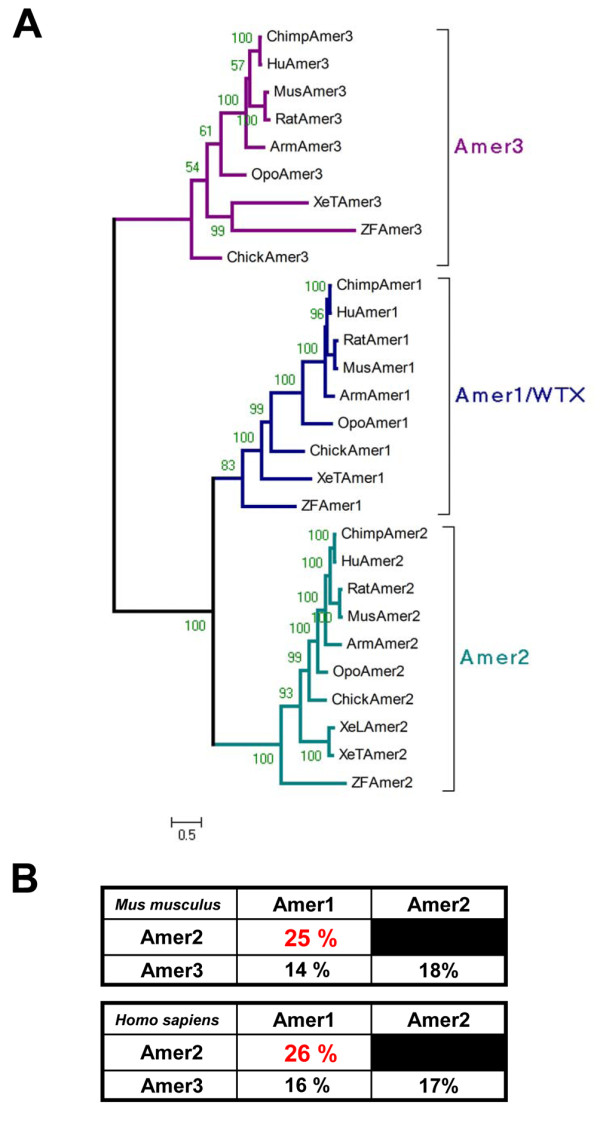
**Phylogenetic analysis of the *Amer *gene family**. **A**. Unrooted Bayesian phylogenetic tree of Wtx/Amer1, Amer2 and Amer3 from each class of the vertebrate subphylum using the whole sequence estimated under the JTT + I + G model (1 MrBayes run of 1,000,000 generations; 453,000 generation burn-in). Numbers indicate posterior probabilities. Abbreviations of taxa are described in Table S1 (additional file 2). Multiple alignment is provided in Figure SM7 (additional file 4). **B**. Similarity percentage between human and mouse Amer proteins for the whole sequence showing that WTX/Amer1 and Amer2 are more closely related. Identical results were obtained for chicken and teleost fishes (data not shown).

To analyze in more detail the evolutionary history of the *Amer *gene family we constructed a phylogenetic tree using the protein sequences identified above. Phylogenetic analysis revealed that *Wtx/Amer1*, *Amer2 *and *Amer3 *form three independent groups in each class of vertebrate species from fish to placental mammals (Figure 3A). Among the three genes *Wtx/Amer1 *and *Amer2 *show a higher extent of sequence similarity (see similarity percentage Figure 3B). In all the species analyzed, the three genes are located on three different chromosomes suggesting that they evolved by genome duplications rather than tandem duplication events (additional file 1, Figure SM3). If *Wtx/Amer1*, *Amer2 *and *Amer3 *genes arise from a common ancestor, one should expect to find them in the vicinity of a common gene family. Schematic representation of chromosomal locations for each gene is given in Figure SM3 (additional file 1). The following genes were found in syntenic position with *Wtx/Amer1*, *Amer2 *and *Amer3*: *MTMR6/8 *(myotubulin related protein 6/8) and *ARHGEF4/9 *(cdc42 guanine nucleotide exchange factor (GEF) 4/9). In *Drosophila *the ortholog for both *MTMR6 *and *MTMR8*, is the gene (CG3530) located on chromosome 2R. Similarly, the ortholog for ARHGEF4 and ARHGEF9 is RhoGEF3, a gene located on the *Drosophila *chromosome 3L (additional file 1, Figure SM3). At those two putative syntenic regions, we could not find any gene with significant level of sequence conservation to the *Amer *gene family. More precisely none of the six conserved sequence blocks could be found in the genes neighbouring CG3530 or RhoGEF3. The fact that *ARHGEF4/9 *is a neighbour of both *Wtx/Amer1 *and *Amer3 *and that *MTMR6/8 *neighbours both *Wtx/Amer1 *and *Amer2 *suggested that the ancestors of these two gene families (ARHGEF and MTMR) were located close to each other early in the vertebrate lineage (additional file 1, Figure SM4). This suggests a loss of paralogs after or between the two rounds of complete duplication in vertebrates (additional file 1, Figure SM4). Based on our analysis, we propose an evolutionary model (additional file 1, Figure SM4) that is consistent with the two rounds of complete genome duplication in vertebrates [12]. In our model, a putative *Amer4 *gene may have been lost after duplication and the presence of a "protoAmer" at the basis of the vertebrate lineage would represent the ancestral vertebrate condition preceding the gene split into *Amer1/2 *and *Amer3/4 *(additional file 1, Figure SM4). Surprisingly, the ortholog distribution of *Wtx/Amer1*, *Amer2 *and *Amer3 *in teleosts does not reflect the additional round of genome duplication that occurred in these species (Figure 3A). A secondary loss of the duplicated genes can explain the present situation. Analysis of the degree of identical residues between human and zebrafish *Amer *gene products shows that it is relatively conserved (additional file 3, Figure SM5). In addition, residues showing a high degree of similarity after multiple alignment of Amer proteins from human and zebrafish correspond to the specific conserved domains found in Amer proteins (Block B1 to B6, Figure 1; additional file 1, Figure SM1).

The radial phylogenetic tree (additional file 3, Figure SM6) confirmed the subdivision of the three genes into three different groups.

### Divergent and conserved molecular functions of *Wtx/Amer1 *genes

**Figure 4:**
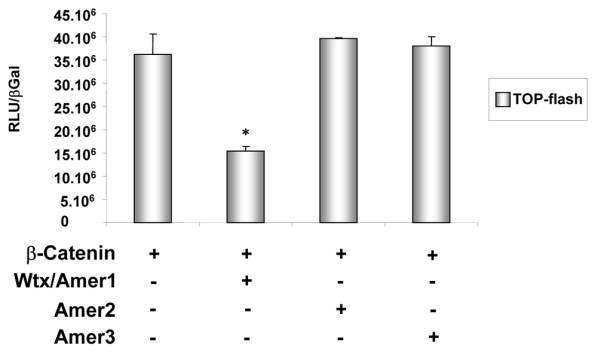
**Regulation of β-catenin activity by Amer proteins**. Co-transfection experiments of β-catenin and mouse Amer expression constructs together with a luciferase reporter gene (TOP-flash) into HEK293T cells are shown. The TOP-FLASH construct is designed to measure transcriptional activation mediated by β-catenin. As previously reported, WTX/AMER1 acts as a negative regulator of β-catenin activity. In contrast, AMER2 and AMER3 do not interfere with β-catenin dependent activation of the TOP-flash reporter gene. Luciferase values were normalized to β-galactosidase. Data are the average of two independent experiments ± SEM. * *p *≤ 0.01.

As noted by François Jacob in 1977, it is at the molecular level that the tinkering aspect of evolution becomes most apparent [13]. Even when gene families evolve by duplication, their members can undergo significant changes leading to structural and functional diversity. According to this view, the *protoAmer *gene may have encoded a short protein to which the highly divergent C-terminal half of *WTX/AMER1 *and *AMER3 *might have been added at a later time point during evolution (Figure 1). This C-terminal part of the *WTX/AMER1 *protein is of importance as it has been demonstrated to be involved in the degradation of the Wnt signaling pathway effector β-catenin [2]. According to the primary structure we expect that Amer2 and Amer3 are unable to inhibit β-catenin activity. Indeed, Amer2 failed to counteract β-catenin activation in an *in vitro *reporter system (Figure 4). While Wtx/Amer1 and Amer3 are both long proteins, their C terminal half lacks significant sequence identity and our phylogenetic analysis places the two genes into two different groups (see radial phylogenetic tree and cladogramme). Functional experiments revealed that Amer3 was also unable to decrease β-catenin dependent gene activation (Figure 4). Taken together these data indicate that *Wtx/Amer1 *might have been co-opted to interact with β-catenin. While addition of the β-catenin interaction domain to *Wtx/Amer1 *seems to be the most likely scenario, we cannot completely rule out the possibility of a functional loss of this capability in Amer2 and 3 proteins. In contrast, the function of the *Amer *gene family as proteins that recruit APC to the membrane might be conserved, as both *WTX/AMER1 *and *AMER2 *possess sequences that allow the molecular interaction with APC (Figure 1; [3]). The APC interacting domains overlap four of the six conserved blocks (Figure 1) indicating that AMER3 might also be involved in APC shuttling. If so, it seems that the ability to recruit APC is one of the ancestral functions of the *Amer *family.

## Conclusion

In this study we reported the first phylogenetic analysis of the *Amer *gene family and identify *Amer3 *as a novel family member. The common ancestry of the three *Amer *genes is supported by the presence of six conserved sequence blocks in all the orthologs, as well as the syntenic conservation with NTMR and ARHGEF paralogs in their three genomic loci. The absence of any sequence similarity to *Amer *genes in invertebrate genomes and the presence of three *Amer *paralogs in all studied vertebrate species strongly support the hypothesis that these genes are novel inventions that originated early in vertebrate evolution. Additional functional studies using animal models misexpressing *Amer2 *and *Amer3 *or knock out alleles will be required to determine their exact function in embryogenesis. Given the importance of *Wtx/Amer1 *in normal development and its involvement in human pathologies, it is tempting to speculate that mutations in *Amer2 *and *Amer3 *may also underlie developmental disorders.

## Methods

### Database search and sequence queries

Search for Amer homologous proteins in vertebrates was made through the NCBI and Ensembl (http://www.ensembl.org; release 56, september 2009) protein databases (see the complete list in additional file 2; Table S1). *In silico *proteins were obtained using GenomeScan software (http://genes.mit.edu/genomescan.html). In order to find potential orthologs in invertebrate genomes, mouse *Wtx/Amer1*, *Amer2 *and *Amer3 *were blasted against the amphioxus (http://genome.jgi-psf.org/Brafl1/Brafl1.home.html), the sea urchin (http://www.spbase.org/SpBase/) the sea anemone (*Nematostella vectensis*, http://genome.jgi-psf.org/Nemve1/Nemve1.home.html), annelid (*Lottia gigantea*, http://genome.jgi-psf.org/Lotgi1/Lotgi1.home.html), mollusk (*Capitella capitata*, http://genome.jgi-psf.org/Capca1/Capca1.home.html) and placozoan (*Trichoplax adhaerens*, http://genome.jgi-psf.org/Triad1/Triad1.home.html) genomes using tblastn under unrestrictive conditions (e-value = 100). No hits were found. The absence of Wtx/Amer1 from the *Drosophila *genome has been previously reported [1].

### Alignment and phylogenetic analysis

Amino acid sequences of Wtx/Amer1, Amer2, Amer3 were aligned using Clustal W [14] and manually corrected with BioEdit [14,15]. Bayesian Inference trees were performed using MrBayes 3.1.2 [16,17] with the model recommended by ProTest 1.4 [18] under the Akaike information and the Bayesian information criterions. We used the JTT + I + G model for both trees. Convergence was reached when the value for the standard deviation of split frequencies stayed below 0.01. Burn-in was determined by plotting parameters across all runs for both analysis: all trees prior to convergence were discarded and consensus trees were calculated for the remaining trees. We used one MrBayes run of 1,000,000 generations and 453,000 generation burn-in for the tree presented in Figure 3A and one MrBayes run of 2,000,000 generations and 1,893,000 generation burn-in for the tree presented in Figure SM6 (additional file 3). The trees were viewed and edited with the TreeExplorer programme in MEGA 4.0 [19]. Maximum likelihood analysis was conducted using RAxML version 7.0.3 [20] using a JTT model of evolution, 1000 bootstrap replicates and the rapid bootstrapping algorithm. The phylogenetic tree obtained using ML is consistent with the one obtained by Bayesian inference (data not shown). Alignment files used to calculate the phylogenetic trees are presented in Figure SM7 and SM8 (additional file 4 and 5 respectively).

### Plasmids

The plasmid expressing human β-catenin was kindly provided by Jurgen Behrens (University of Erlangen, Germany). The TOP-FLASH and FOP-FLASH luciferase reporter constructs have been described previously [21]. Briefly, the TOP-FLASH construct is designed to measure transcriptional activation mediated by β-catenin and FOP-FLASH is the mutated counterpart of the TOP-FLASH plasmid. Mouse Wtx/Amer1, Amer2 and Amer3 expression plasmids were obtained by PCR amplification of the coding sequence from BAC constructs (bMQ-277D5 for Wtx/Amer1, bMQ-344G22 for Amer2 and bMQ-123E19 for Amer3) (Geneservice, Cambridge, UK)) and insertion into the pAcGFP1-C1 plasmid (Clontech). All constructs were verified by DNA sequencing.

### Detection of *Wtx/Amer1 *and *Amer2 *variants by RT-PCR

Total RNA from E9.5 to E14.5 dpc mouse embryos or *Wtx/Amer1*, *Amer2 *or *Amer3 *transfected HEK293T cells was extracted using TRIzol reagent (Invitrogen) and RNA purification was performed using the RNeasy Mini kit (Qiagen) and Rnase-free DNAse digestion (Qiagen). cDNA were synthesized from 1 μg of RNA using the MMLV reverse transcriptase system and random hexamers (Invitrogen). All PCR assays were performed using the GoTaq^® ^Green Mix for PCR (Promega). Amplification of the N-terminus of Wtx/Amer1, Amer2 and Amer3 was performed using three different pairs of primers for each gene. Primer sequences used in Figure 2 are as follows: Wtx/Amer1, foward: tgaggcaacagaaggacca, reverse: tggagagtcaacaggatgaagctgttcaa; Amer2, foward: atggactcgcattgtgagtgcg, reverse: cgagctcccatctgcaaa; Amer3, foward: gaggagaggaaagaccttcatc, reverse: tcccagaacttgttgaagtctg. The other primer pairs used in this assay are available on demand. Briefly, all forward primers were located in the non-coding exon 1 or at the 5'end of exon 2 and all reverse primers just downstream of the internal splicing acceptor. PCR products were sequenced (SeqLAB, Sequence Laboratories, Göttingen, Germany) to check the specificity of Amer variants.

### Reporter assay

HEK293T cells were cultured in DMEM supplemented with 10% (v/v) fetal calf serum, in a 5% CO2 humidified atmosphere. HEK293T cells were transiently transfected with the TOP-FLASH reporter or its mutated counterpart (FOP-FLASH) and β-catenin with either *Wtx/Amer1*, *Amer2 *and *Amer3 *expression plasmids (200 ng each). Cells were also co-transfected with 50 ng of CMV (cytomegalovirus)-β-galactosidase as an internal control. Transient transfection of expression plasmids was performed with Fugene according to manufacturers instructions (Invitrogen). After 36 h, luciferase activity was measured according to the Luciferase Assay System (Promega) and data were normalized to β-galactosidase activity and plotted. Luciferase activities obtained after transfection with the FOP-FLASH construct are not shown as they were close to zero for each condition.

## Abbreviations

AMER: APC membrane recruitment; APC: adenomatous polyposis coli; WDG: whole duplication genome; NLS: nuclear localization signal; OSCS: osteopathia striata congenital with cranial sclerosis; DKK: Dickkopf.

## Authors' contributions

AS initially identified Amer2 and Amer3 from the database. GC contributed to the ortholog searches and performed the splicing analysis. AB carried out alignment, phylogenetic construction and functional experiments. AS and AB wrote the paper. All authors read and approved the final manuscript.

## Supplementary Material

Additional file 1 **Additional figures (SM1-4) and corresponding captions**. Figure SM1 is a figure showing the exon-intron structure of *Amer *genes and the consensus sequences of the six specific conserved domains found in all Amer proteins. Figure SM2 displays the protein domains for each Amer protein. Figure SM3 shows the conserved synteny of *Amer *genes in vertebrates. Figure SM4 proposes an evolutionary scenario for the origin of the *Amer *gene family. Click here for file

Additional file 2 **Table S1**. List of *Wtx/Amer1*, *Amer2 *and *Amer3 *orthologs. Click here for file

Additional file 3 **Additional figures (SM5-6) and corresponding captions**. Figure SM5 displays multiple alignment of Amer proteins from human and zebrafish. Figure SM6 shows the phylogenetic tree generated with a wide array of Amer proteins. Click here for file

Additional file 4 **Additional figure SM7**. Figure SM7 contains multiple alignment performed with Wtx/Amer1, Amer2 and Amer3 from each class of the vertebrate subphylum using the whole sequence (28 sequences in total). Click here for file

Additional file 5 **Additional figure SM8**. Figure SM8 contains multiple alignment performed with a broader array of Wtx/Amer1, Amer2 and Amer3 proteins (73 sequences in total). Click here for file
