# Supplementary material for: The WTX/AMER1 gene family: evolution, signature and function
Source: BMC Evol Biol. 2010 Sep 15;10:280. doi: 10.1186/1471-2148-10-280 (PMC2949870; doi:10.1186/1471-2148-10-280)
Supplement: Additional file 1 — Additional figures (SM1-4) and corresponding captions. Figure SM1 is a figure showing the exon-intron structure of Amer genes and the consensus sequences of the six specific conserved domains found in all Amer proteins. Figure SM2 displays the protein domains for each Amer protein. Figure SM3 shows the conserved synteny of Amer genes in vertebrates. Figure SM4 proposes an evolutionary scenario for the origin of the Amer gene family. [file 1471-2148-10-280-S1.PDF]

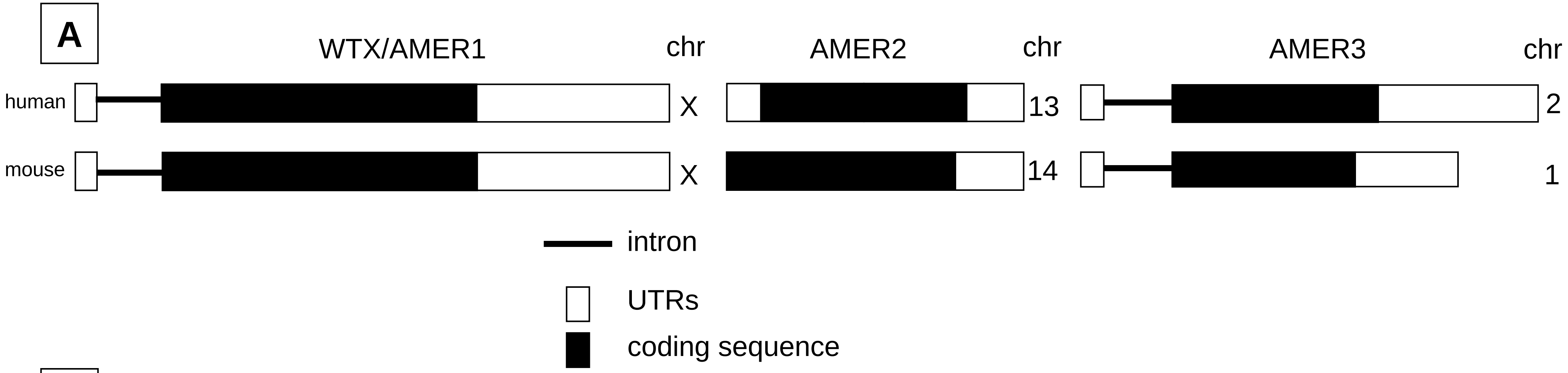

B

B 1

**MusAmer2**/60-67	RSRTHDGL
**RatAmer2**/60-67	RSRTHDGL
**HuAmer2**/102-109	RSRTHDGL
**ChimpAmer2**/102-109	RSRTHDGL
**ArmAmer2**/103-110	RSKTHDGL
**OpoAmer2**/60-67	RSKTHDGL
**ChickAmer2**/60-67	RSRTHDGL
**XeTAmer2**/90-97	RSKTLDGL
**XeLAmer2**/71-78	RSKTLDGL
**ZFAmer2**/63-70	RSKTHDGL
**MusAmer1**/92-99	KSKTHDGL
**RatAmer1**/92-99	KSKTHDGL
**HuAmer1**/92-99	KSKTHDGL
**ChimpAmer1**/92-99	KSKTHDGL
**ArmAmer1**/91-98	KSKTHDGL
**OpoAmer1**/95-102	KSKTHDGL
**ChickAmer1**/98-105	KSKTHDGI
**XeTAmer1**/252-259	KSKTHDGI
**ZFAmer1**/74-81	KSQTYDGV
**HuAmer3**/91-98	KCKTHDSM
**ChimpAmer3**/91-98	KSKTHDSM
**MusAmer3**/87-94	KSKTHDSV
**RatAmer3**/87-94	KSKTHDNV
**ArmAmer3**/68-73	KNRMQDCG
**OpoAmer3**/91-98	KSKTHDCV
**ChickAmer3**/89-96	KSKTHDCV
**XeTAmer3**/72-79	KSKTHDCV
**ZFAmer3**/79-86	RSKTHDCV
consensus	KSKTHDGL

B 2

144-153	KQKRGLKGIF
144-153	KQKRGLKGIF
189-198	KQKRGLRGLF
189-198	KQKRGLRGLF
185-194	RQKRGLKGLF
149-158	KQKKGLKGIF
138-147	RQKKGLKGIF
171-180	RQKRGLKGLF
152-161	RQKRGLKGLF
135-144	RRGRGLKGLF
164-173	KPKKSLKSFF
164-173	KPKKGLKGFL
164-173	KPKKGLKGFF
164-173	KPKKGLKGFF
163-172	KPKKGLKGFF
169-178	RPKKGLKGLF
178-187	RPKKGLKGLL
337-346	RPKKGLKGLF
122-131	RQRRGLRGLF
155-164	RPKKCFRNLF
155-164	TPKKCFRNLF
151-160	RSKKCFRNLF
151-160	RSKKCFRNLF
131-140	RPKKCLRNLF
153-162	RSKKGFRDIF
184-193	RSKKGLRDIF
152-161	KTKKGFRNIF
169-178	KPRKAIKDLF
Consensus	RPKKGLKGLF

B 3

345-351	CGDIIAD
345-351	CGDIIAD
262-268	CGDIIAD
262-268	CGDIIAD
237-243	CGDIIAV
349-355	CGDIIAD
333-339	CGDIIAD
311-317	CGDVIAD
349-355	CGDVIAD
313-319	CGDIIAD
327-333	CGDIIAE
327-333	CGDIIAE
328-334	CGDIIAE
328-334	CGDIIAE
326-332	CGDIIAE
326-332	CGDIIAE
350-356	CGDIIAE
505-511	CGDIIAD
301-307	CGDIIAD
250-256	CGEVFAD
250-256	CGEVFAD
242-248	CGEIFAD
242-248	CGEIFAD
226-231	CGEIFAD
244-250	CGEIFAD
279-285	CGEIFAD
239-245	CGEIFAD
259-265	CGEIFAD
Consensus	CGDIIAD

B 4

383-392	QGGGEEMASP
383-392	QGGGEEMASP
300-309	QGGGEEMASP
300-309	QGGGEEMASP
276-285	QGGGEEMASP
387-396	QGGGEEMASP
374-383	QGGGEEMASP
350-359	QGGGEEMASP
388-397	QGGGEEMASP
402-411	MGGGEEMASP
365-374	QGGGEEMALP
365-374	QGGGEEMALP
366-375	QGGGEEMALP
366-375	QGGGEEMALP
364-373	QGGGEEMALP
364-373	QGGGEEMAMQ
386-395	QGGGEEMAMS
541-550	QGGGEEMATP
338-347	QGGGEEMATP
289-298	QGSVEQLASP
289-298	QGSVEQLASP
281-290	QGGMEQLMSP
281-290	QGSMEQLMSP
265-274	QGGVEQLASP
282-291	QGGIEQLASP
316-325	QGGVEQLASP
276-285	QGGVEKLASP
296-305	QGGTECLASP
Consensus	QGGGEEMASP

B 5

484-495	SDEGYWDSTTPG
484-495	SDEGYWDSTTPG
401-412	SDEGYWDSTTPG
401-412	SDEGYWDSTTPG
376-387	SDEGYWDSTTPG
492-503	SDEGYWDSTTPG
469-480	SDEGYWDSTTPG
441-452	SDEGYWDSPTPG
480-491	SDEGYWDSTTLG
496-507	SDEGYWDSPTPG
487-498	SDEGYYDSTTPG
468-479	SDEGYYDSTTPG
467-478	SDEGYYDSTTPG
468-479	SDEGYYDSTTPG
465-476	SDEGYYDSTTPG
446-457	SDEGYYDSNTPG
471-482	SDEGYYDSTTPG
628-639	SDEGYYDSNTPG
425-436	SDEGYYDSTTPG
383-394	SDEGYYDSFSPG
383-394	SDEGYYDSFSPG
375-386	SDEGYYDSFSPG
375-386	SDEGYYDSFSPG
361-372	SDEGYYDSFSPG
375-386	SDEGYYDSFSPG
410-421	SDEGYYDSFSPG
362-373	SDEGYCDSMSPG
386-397	SDEGYCDYVSPG
Consensus	SDEGYYDSTTPG

B 6

513-526	PRDSDSGDALCDLY
513-526	PRDSNSGDVLCDLY
427-440	PRDSYSGDALYDLY
427-440	PRDSYSGDALYDLY
402-415	PRDSCSGDALYDLD
518-531	PRDSYSGDALYDLY
495-508	PRDSYSGDALYDLY
467-480	ARDSCSGDALYDLY
506-519	PRDSCSGDALYDLY
522-535	LRDSCSGDALYDLY
516-529	PRDSYSGDALYEFY
497-510	PRDSYSGDALYEFY
496-509	PRDSYSGDALYEFY
497-510	PRDSYSGDALYEFY
494-507	PRDSYSGDALYEFY
475-488	PRDSYSGDALYEFF
500-513	PRDSYSGDALYEFY
655-668	PRDSYSGDALYEFY
451-464	PRDSYSGDALYELF
413-426	PRDSYSGDALYELF
413-426	PRDSYSGDALYELF
405-418	PRDSYSGDALYELF
405-418	PRDSYSGDALYELF
391-404	PRDSYSGDALFELF
405-418	PRDSYSGDALYELF
439-452	PRDSYSGDALYELF
388-401	PRDSYSGDALFELF
415-428	PRDTYSGDALYELF
Consensus	PRDSYSGDALYELY

Figure SM1 A. Schematic drawing of exon-intron structures of *Amer* genes from human and mouse. white boxes indicate UTRs and black boxes indicate protein-coding regions B. Colored blocks (B1 to B6) contain sequence comparison and consensus sequences of the specific conserved domains found in Amer proteins. The relative position of each block on Amer proteins is illustrated in Figure 1.

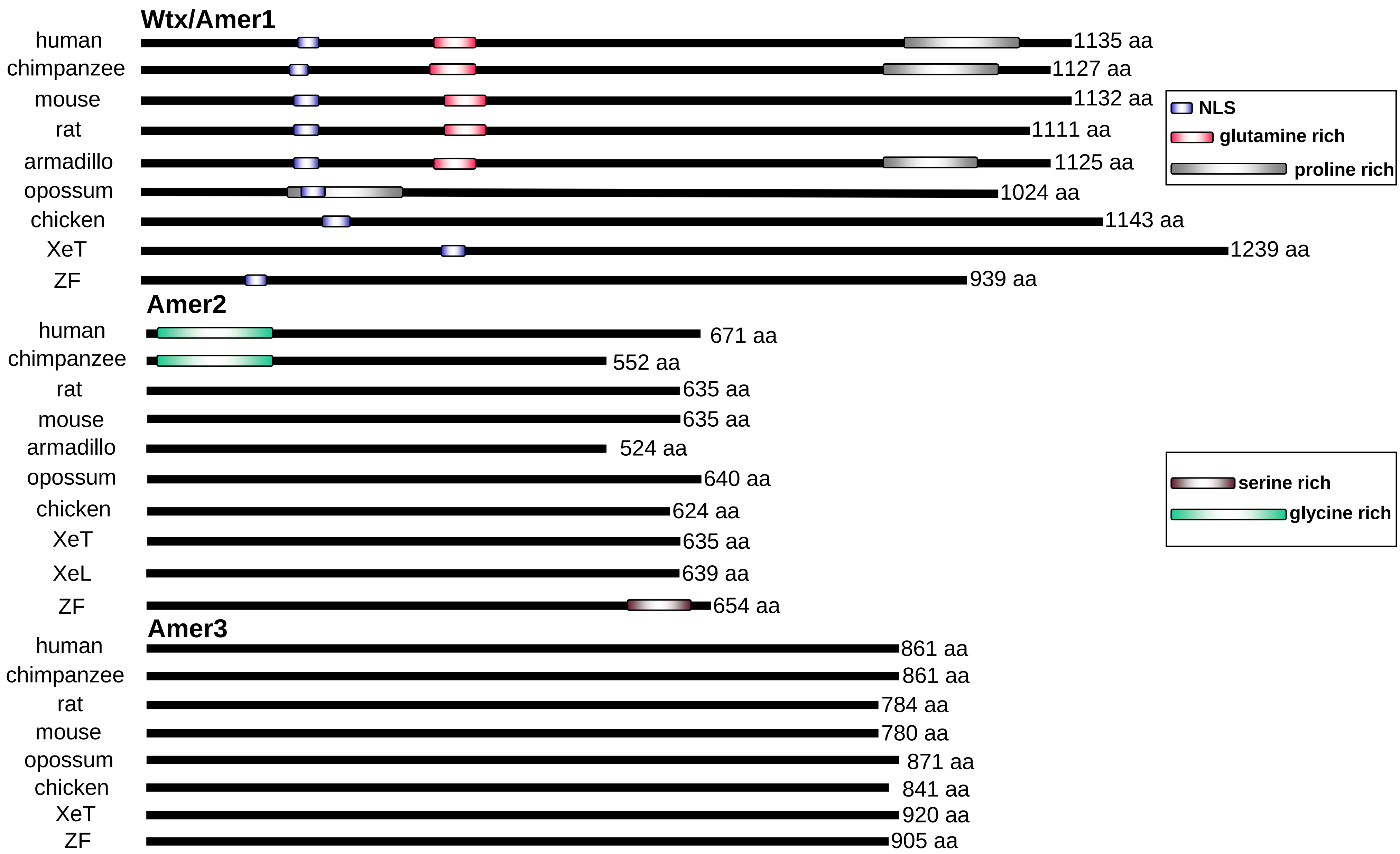

Figure SM2 Structure and domain for Amer proteins. Wtx/Amer1 is characterized by the presence of a NLS (Nuclear Localization Signal) at the N-terminal end, a glutamic acid-rich region and a proline-rich domain at the C-terminal end. All the Amer1 orthologs possess the NLS at the N-terminal end of the protein. The proline-rich domain is restricted to the primate, xenarthra and marsupial species. The glutamic acid-rich region is not found in birds, fishes, amphibian and opossum. Amer2 proteins do not possess any protein domain of known function shared by all the orthologs. Only fish proteins, medaka, zebrafish, stickelback (*Gasterosteus aculaetus*), tetraodon (*Tetraodon nigroviridis*), display a serin-rich domain in the C-terminal region. Only the sequence of zebrafish is shown. Amer3 proteins do not possess any protein domain of known function. Abbreviations of taxa are described in addition file 2 (Table 1).

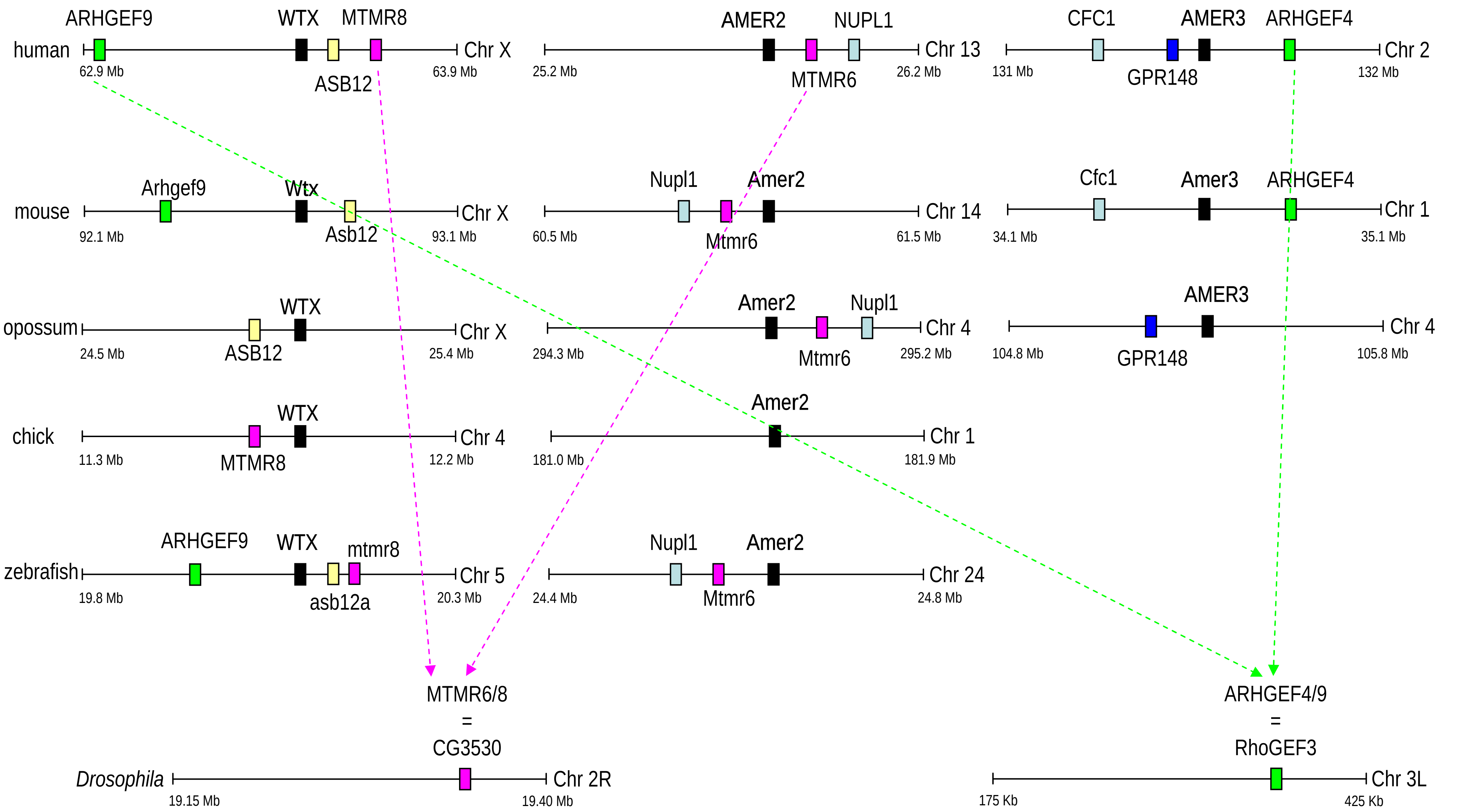

Figure SM3. Conserved synteny of *Amer* genes in vertebrates. Schematic representation of the chromosomal position of *Amer* and of their neighbouring genes in *Homo sapiens*, *Mus musculus*, *Monodelphis domestica*, *Gallus gallus*, *Danio rerio*. *Amer* genes are indicated by black squares. Members of the Rho guanin nucleotide exchange factor (green squares) family are found next to Wtx and Amer3. The ortholog of these two genes in *Drosophila* is RhoGEF3 located on chromosome 3L. Similarly members of the myotubuline related protein (pink squares) are found next to Wtx and Amer2. The ortholog of MTMR6 and MTMR8 is CG3530 in *Drosophila* located on chromosome 2R.

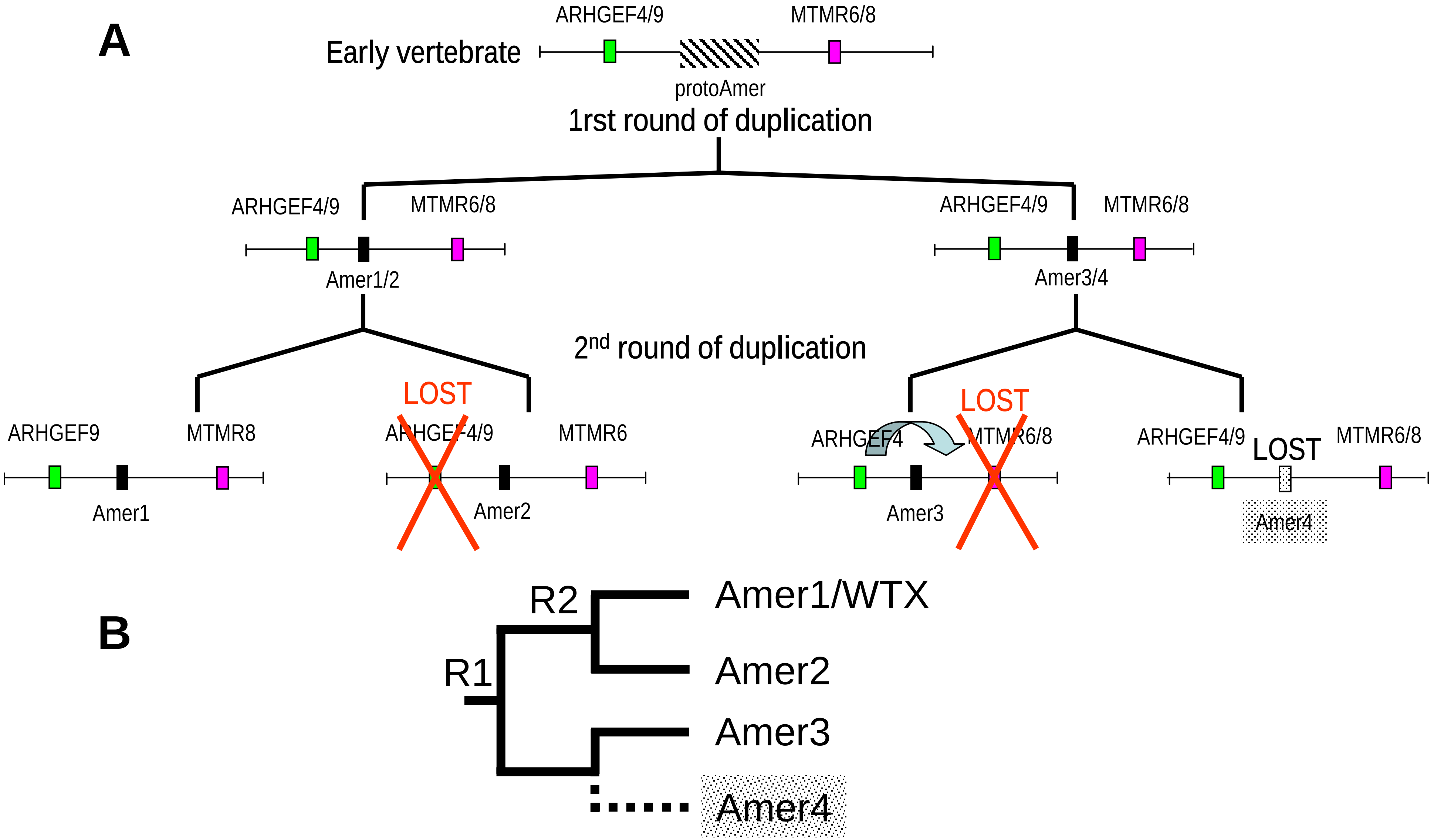

Figure SM4 A. Putative evolutionary scenario for the origin of the *Amer* gene family. As members of the Rho guanine nucleotide exchange factor (ARHGEF; green squares) family are found next to Wtx and Amer3 and members of the myotubuline related protein (MTMR; pink squares) are found next to Wtx and Amer2 in vertebrate (see Figure SM3), these two gene families might have been located close to each other early in the vertebrate lineage. After the second round of duplication, ARHGEF member next to Amer2 has been lost. The same occured for MTMR member next to Amer3. An *Amer4* gene does not exist and might hence have secondarily been lost. **B.** Schematic representation of the proposed scenario for the evolution of the *Amer* gene family in vertebrate
