# Supplementary material for: The WTX/AMER1 gene family: evolution, signature and function
Source: BMC Evol Biol. 2010 Sep 15;10:280. doi: 10.1186/1471-2148-10-280 (PMC2949870; doi:10.1186/1471-2148-10-280)
Supplement: Additional file 2 — Table S1. List of Wtx/Amer1, Amer2 and Amer3 orthologs. [file 1471-2148-10-280-S2.PDF]

**TABLE 1. Ortholog searches**

| WTX/AMER1 | | | | | |
|---|---|---|---|---|---|
| **Species** | **Ensembl Prot seq** | ***In silico* prediction** | **NCBI** | **Chromosomal / Scaffold location** | **abbreviations** |
| **Placental mammals** | | | | | |
| Human (*Homo sapiens*) | 1135 aa | | | Chr X | Hu |
| Mouse (*Mus musculus*) | 1132 aa | | | Chr X | Mus |
| Chimpanzee (*Pan troglodytes*) | 1127 aa | | | Chr X | Chimp |
| Gorilla (*Gorilla gorilla*) | 1136 aa | | | scaffold_3408: 29,933-33,343 | Go |
| Macaque (*Macaca mulatta*) | 803 aa | 1134 aa* | XM_001096922 | Chr X | Mac |
| Orangutan (*Pongo pygmaeus*) | 805 aa | 1136 aa* | | Chr X | Pong |
| Mouse lemur (*Microcebus murinus*) | 800 aa | 1145 aa* | | scaffold_6289: 48,420-55,529 | Micro |
| Hyrax (*Procavia capensis*) | 1149 aa | | | GeneScaffold_6690: 33,430-36,879 | Hyr |
| Megabat (*Pteropus vampyrus*) | 1096 aa | | | scaffold_8000: 30,363-33,653 | Meg |
| Rat (*Rattus norvegicus*) | 1111 aa | | | Chr X | Rat |
| Cow (*Bos taurus*) | 789 aa | | XM_584347 (1119 aa) | Chr X | Cow |
| Dog (*Canis familiaris*) | 1130 aa | 1130 aa* | XM_843253 | Chr X | Dog |
| Guinea Pig (*Cavia porcellus*) | 1100 aa | | | scaffold_122: 1,398,803-1,402,186 | Gui |
| Horse (*Equus caballus*) | 1126 aa | | | Chr X | Ho |
| Hedgehog (*Erinaceus europaeus*) | 815 aa | 1098 aa* | | scaffold_247247: 11,607-17,340 | Hed |
| Cat (*Felis catus*) | | 1056 aa* | | GeneScaffold_4377: 42,131-45,912 | Cat |
| Elephant (*Loxodonta africana*) | 1132 aa | | | scaffold_4532: 121,634-125,079 | Ele |
| Rabbit (*Oryctolagus cuniculus*) | 781 aa | 1078 aa* | | GeneScaffold_6115: 13,878-20,888 | Rab |
| Bushbaby (Otolemur garnettii) | 798 aa | 1080 aa* | | scaffold_17174: 5,207-11,732 | Bush |
| Shrew (*Sorex araneus*) | 798 aa | 1115 aa* | | scaffold_230962: 31,390-42,415 | Shr |
| Tree Shrew (*Tupaia belangeri*) | | 864 aa* | | scaffold_148274: 49,242-56,040 | Tup |

| | | | | | |
|---|---|---|---|---|---|
| Dolphin (*Tursiops truncatus*) | 1114 aa | | | GeneScaffold_3116: 82,676-86,436 | Dol |
| Armadillo (*Dasypus novemcinctus*) | 1125 aa | | | scaffold_52243: 8,780-12,157 | Arm |
| **Marsupial mammals** | | | | | |
| Opossum (*Monodelphis domestica*) | 701 aa | 1024 aa* | XM_001368717 | Chr X | Opo |
| **Birds** | | | | | |
| Chicken (*Gallus gallus*) | | | XM_420290 | Chr 4 | Chick |
| **Amphibians** | | | | | |
| Xenopus (*Xenopus tropicalis*) | | 1239 aa* | EST (BJ639508) | Scaffold 10 | XeT |
| **Teleost fishes** | | | | | |
| Zebrafish (*Danio rerio*) | 939 aa | | BC155291 | Chr 5 | ZF |
| Medaka (*Oryzias Latipes*) | | 977 aa* | | Chr 14 | Med |
| Stickleback (*Gasterosteus aculeatus*) | | 993 aa* | | groupVII: 17,183,033-17,184,016 | Stick |
| Fugu *(Takifugu rubripes)* | | 1006 aa* | | scaffold_6: 1,219,128-1,220,087 | Fug |

| **AMER2** | | | | | |
|---|---|---|---|---|---|
| **Species** | **Ensembl Prot seq** | ***In silico* prediction** | **NCBI** | **Chromosomal / Scaffold location** | **abbreviations** |
| **Placental mammals** | | | | | |
| Human (*Homo sapiens*) | 552 aa | | | Chr 13 | Hu |
| Mouse (*Mus musculus*) | 635 aa | | | Chr 14 | Mus |
| Chimpanzee (*Pan troglodytes*) | 552 aa | | XM_509590 | Chr 13 | Chimp |
| Macaque (*Macaca mulatta*) | 550 aa | | | Chr 17 | Mac |
| Orangutan (*Pongo pygmaeus*) | 546 aa | | | Chr 13 | Pong |
| Rat (*Rattus norvegicus*) | 635 aa | 646 aa | XM_001063071 | Chr 15 | Rat |
| Microbat (*Myotis lucifugus*) | | 560 aa | | scaffold_166430: 75,130-77,188 | Myo |
| Pika (*Ochotona princes*) | 545 aa | 569 aa | | scaffold_4354: 100,979-103,165 | Pika |
| Rabbit *(Oryctolagus cuniculus*) | 545 aa | | | scaffold_210979: 56,269-58,326 | Rab |
| Squirrel (*Spermophilus tridecemlineatus*) | | 501 aa | | GeneScaffold_5979: 255,465-277,405 | Squi |

| | | | | | |
|---|---|---|---|---|---|
| Kangaroo rat (*Dipodomys ordii*) | 548 aa | | | scaffold_20825: 9,269-11,401 | Krat |
| Boar (*Sus scrofa*) | | | XM_001924197 (518 aa) | Chr 11 | Boar |
| Armadillo (*Dasypus novemcinctus*) | | 524 aa | | GeneScaffold_5237: 35,019-48,733 | Arm |
| **Marsupial mammals** | | | | | |
| Opossum (*Monodelphis domestica*) | 553 aa | 640 aa | XM_001375738 | Chr 4 | Opo |
| **Birds** | | | | | |
| Chicken (*Gallus gallus*) | 624 aa | | | Chr 1 | Chick |
| **Amphibians** | | | | | |
| Xenopus (*Xenopus tropicalis*) | 635 aa | | BC135184 | scaffold_636: 563,743-565,650 | XeT |
| Xenopus (*Xenopus laevis*) | | | BC072359 (639 aa) | | XeL |
| **Squamates** | | | | | |
| Anole Lizard (*Anolis carolinensis*) | | 637 aa | | scaffold_38: 5,267,831-5,268,811 | Liz |
| **Teleost fishes** | | | | | |
| Zebrafish (*Danio rerio*) | 654 aa | | NM_001100152 | Chr 24 | ZF |
| Medaka (*Oryzias Latipes*) | | 685 aa | | Chr 20 | Med |
| Stickleback (*Gasterosteus aculeatus*) | 609 aa | | | groupXXI: 4,702,544-4,704,622 | Stick |
| *Fugu (Takifugu rubripes)* | | 683 aa | | scaffold_162: 19,596-21,846 | Fug |
| Tetraodon (*Tetraodon nigroviridis*) | | 692 aa | | Chr 6 | Tetra |

| **AMER3** | | | | | |
|---|---|---|---|---|---|
| **Species** | **Ensembl Prot seq** | ***In silico* prediction** | **NCBI** | **Chromosomal / Scaffold location** | **abbreviations** |
| **Placental mammals** | | | | | |
| Human (*Homo sapiens*) | 861 aa | | | Chr 2 | Hu |
| Mouse (*Mus musculus*) | 780 aa | | | Chr 1 | Mus |
| Chimpanzee (*Pan troglodytes*) | 861 aa | | | Chr 2b | Chimp |
| Gorilla (*Gorilla gorilla*) | 861 aa | | | scaffold_15596: 32,603-35,188 | Go |
| Macaque (*Macaca mulatta*) | 861 aa | | | Chr 13 | Mac |
| Orangutan (*Pongo pygmaeus*) | 861 aa | | | Chr 2b | Pong |

| | | | | | |
|---|---|---|---|---|---|
| Megabat (*Pteropus vampyrus*) | 775 aa | | | scaffold_23676: 1,970-4,342 | Mega |
| Rat (*Rattus norvegicus*) | 784 aa | | XM_001055491 | Chr 9 | Rat |
| Cow (*Bos taurus*) | | 807 aa | XM_869018 | Chr 2 | Cow |
| Dog (*Canis familiaris*) | 855 aa | | | Chr 19 | Dog |
| Guinea Pig (*Cavia porcellus*) | 788 aa | | | scaffold_3: 71,090,185-71,092,548 | Gui |
| Horse (*Equus caballus*) | 763 aa | 1043 aa | XM_001915331 | Chr 18 | Ho |
| Rabbit (*Oryctolagus cuniculus*) | 841 aa | | | scaffold_175702: 11,147-13,706 | Rab |
| Dolphin (*Tursiops truncatus*) | 846 aa | | | scaffold_108978: 8,870-11,470 | Dol |
| Armadillo (*Dasypus novemcinctus*) | 472 aa | | | scaffold_32713: 39,924-41,372 | Arm |
| **Marsupial mammals** | | | | | |
| Opossum (*Monodelphis domestica*) | 871 aa | | XM_001376608 | Chr 4 | Opo |
| **Monotremal mammals** | | | | | |
| Platypus (*Ornithorhynchus anatinus*) | | 840 aa | | Contig7170: 25,324-27,151 | Pla |
| **Birds** | | | | | |
| Chicken (*Gallus gallus*) | | | XM_001232633 | Chr 9 | Chick |
| **Amphibians** | | | | | |
| Xenopus (*Xenopus tropicalis*) | | 920 aa | | Scaffold 55 | XeT |
| **Teleost fishes** | | | | | |
| Zebrafish (*Danio rerio*) | | | XM_001341803 (905 aa) | Chr 2 | ZF |

* When ORFs predicted by automated database annotation were shorter than anticipated, we use GenomeScan software to build the correct gene model using the human *Amer* genes as guide.
