# Supplementary material for: The WTX/AMER1 gene family: evolution, signature and function
Source: BMC Evol Biol. 2010 Sep 15;10:280. doi: 10.1186/1471-2148-10-280 (PMC2949870; doi:10.1186/1471-2148-10-280)
Supplement: Additional file 3 — Additional figures (SM5-6) and corresponding captions. Figure SM5 displays multiple alignment of Amer proteins from human and zebrafish. Figure SM6 shows the phylogenetic tree generated with a wide array of Amer proteins. [file 1471-2148-10-280-S3.PDF]

A

```
HuA2            METSRSRGGGGAVSERGGAGASVGVCRRKAEAGAGTGTLAADMDLHCDCAAETPAAEPPS 60
ZFA2            ------------------------------------------MEVQTECSEPPPCDPQPP 18
HuA1            -----------METQKDEAAQAKGAAASGSTREQTAEKGAKNKAAEATEGPTSEPSSSGP 49
ZFA1            ----------------------------MEIATRCEVGAMRGPSSDSVSHDIPQPQSPPS 32
HuA3            -------------------------------MELKRGKTFIKSSLQVSHEKPPDPAAVAA 29
ZFA3            ----------------------------MELSKSEDSSDNRKRSNEAHGRSKQPDEHTSK 32
                                                             .

HuA2            GKINKAAFKLFKKRKSGGTMPS-IFG-VKNKGDGKSSGP---TGLVRSRTHDGLAEVL-- 113
ZFA2            GKLNKAAFKLFGKRKSGSSMPS-IFS-VRNKGESTGKAAGKTLELVRSKTHDGLITDTPS 76
HuA1            GRLKKTAMKLFGGKKGICTLPS-FFGGGRSKGSGKGSSK---KGLSKSKTHDGLSEAA-- 103
ZFA1            VKIRKTAFKFFGGRKSICVLPS-FFG-GRGRSQRKGSSK---TGVTKSQTYDGVSRACWD 87
HuA3            AREGTGPWSVLPGGQQRPHSEK-GPQ-ASPSAQEYDRCPNKGAQLDPKGGPAALCGATFK 87
ZFA3            ITMNNGFVNEHNTDESPSSLPKPDILFPGVSDTLSEGHRHLCSSVTRSKTHDCVRGLGLQ 92
                    .   .     :      .                      :  .     :

HuA2            VLESGRKEEPRGGGDSGGGGGGRPNPGPPRAAGPG-GGSLASSSVAKSHSFFSLLKKNGR 172
ZFA2            ELDSHRKEESASSDQLHAGTPDGVSTAP-----------LRSSITKSFSFFSLLRRSSS 124
HuA1            ---HGPEDVVSEGTGFSLPLPELPCQFP-------------SSQSAHGALETGSRCKTSV 147
ZFA1            DLGRSSSEVASGDFEFCSEPQKSQEDHGKSQ-------SLPRQRRGLRGLFSSIRRHRKN 140
HuA3            PVRKCKTHDSMSGAGRATAATGQLVGSASFP---------GSPGSRRMIDYRHFVPQMPF 138
ZFA3            QAKNSEYKDGTSWRHHQKLLTSVSFPGFETPLRLLRETQDTSGSSHEIDDYRNLTPQVPF 152
                       .                                                :

HuA2            SENGKGEPVDASKAGGKQKRGLRGLFSGMRWHRK-DKRAKAEAAEGRAPGGGLILP---- 227
ZFA2            RAG------DGTTTVGRRGRGLKGLFSSMRWRRKPQIQEDTLEVAKEVKEGDLILSSSSG 178
HuA1            AGATEKAVAEKFPSMPKPKKGLKGFFSSIRRHRK-SKVTGAEQSEPGAKGPERVRARPHE 206
ZFA1            KNVEVEKREALEMSSSFHAKTVPGALPSVSDRGDYHGDSQGEELVPDVPNQTTGSECELP 200
HuA3            VPAVAKSIPRKRISLKRPKKCFRNLFHIRRNKTEDLASLAAEGKSLPSPG---------- 188
ZFA3            VPCIGKSIPKRRRSLRKPRKAIKDLFVHKSYKHEKATPPSTPSRVFG------------- 199
                             :     : . . :     : .

HuA2            -------------------------------------------------GSLTASLECV 237
ZFA2            SVKTEKDMTLTLEPLPQVFEESPLPGDSDKWKVASMQGIQGTNEVECGNCGPSVSQQHTV 238
HuA1            HVSSAPQVPCFEETFQAPRKENANPQDAPGPKVSPTP-------------EPSPPATEKM 253
ZFA1            LAATECTIDVTLVPEK---------------------------------RRSRVEMDKR 226
HuA3            -------------------------------------------------DPSDPGGRRS 198
ZFA3            -------------------------------------------------ENATILMRIR 209


HuA2            KEETPR----------------------------AAREPEEPSQDAPRDP---------- 259
ZFA2            TEESPAPSPLRVQTGGLQNHKHSSSTHLSSIPTCALTPPMEHSTADPQSEQSVDRLCSMF 298
HuA1            ACKDPEKPMEACASAHVQPKPAPEASSLEEPHSPETGEKVVAGEVNPPNGPVGDPLSLLF 313
ZFA1            RRAEEEGIGEDEKTGRQEGLMTYHQPLSAESELDRLAEQNVDVPDGEPPVASCSSENLVF 286
HuA3            KAFLPPGEGPG-----------------------LDGLCQDLLDSELLADASFGLCRALC 235
ZFA3            KAARHRECS-------------------------TAGSRCNDELSETPSDSSSESGPNVC 244


HuA2            ------------AGCGDIIADQEEEAGPSCDKHVPGPG---------------------- 285
ZFA2            TDVTSLKSFDSLTGCGDIIADPEEDSGNGGSATSSGTGSSSGGCMGRRLSGAGTNSERCS 358
HuA1            GDVTSLKSFDSLTGCGDIIAEQDMDSMTDSMASGGQRAN--------------------- 352
ZFA1            GDVSSLKSFDSLTGCGDIIADQDDVSVAESSVSADRGS---------------------- 324
HuA3            EDVASLQSFDSLTGCGEVFADESSVPSLELNEGPESPT---------------------- 273
ZFA3            EDAVSLKSFGSQAGCGEIFADDLVSPDGVLNSQHDRVACG-------------------- 284
                            :***:::*:    .

HuA2            --------------------------KPALSKKNP---GVVAYQGGGEEMASPDEVDDTY 316
ZFA2            PAKPPLPPQVSSLASIHASCYMPAHQRPRAAPKKPQGSGVVAYMGGGEEMASPEGVDDAD 418
HuA1            ----------------------------RDGTKRS--SCLVTYQGGGEEMALPDDDDEEE 382
ZFA1            ----------------------------RNAGKRS--SCFVTYQGGGEEMATPDEIDADY 354
HuA3            ----------------------------QAAQGLESKVPRGPLQGSVEQLASPAQNEASD 305
ZFA3            -----------------------------TPKQSP---TTLGIQGGTECLASPANAEVLD 312
                                            *. * :* *   :

HuA2            LQEFWDMLSQTEEQGPEPQEGAAKVAAALETKVVPETPKDTRCVEAAKDASSVKRRRLNR 376
ZFA2            MQGLWHMLPQKDEDSPAPRR--AEPVLHHAPARLEKRPPQVKALGLSKIPVSGSSKTGKQ 476
HuA1            EEEEEVELEEEEEEVKEEEED-DDLEYLWETAQMYPRPNMNLGYHPTTSPGHHGYMLLDP 441
ZFA1            LQSLWESETSNEVCYIPSDRG------SDSPSLTPDQQLSSIRATSSSSPMGITETALTP 408
HuA3            FTRFWDSVNRSVRQQQRALLG----PWLSGPQGTDRDQSRLDTAGLAELPLCPCRDPRSG 361
ZFA3            MFGLWETLNRTLLSEQSLKAMGPATKHTAPITISPSTTNSADITVSTPHPVEPEIKELNA 372
                                                              :  .

HuA2            IPIEP-HPKEEPKHPEKEQQEGVPNSDEGYWDSTTPGPEEDSSSSG----KKAGIPRDSY 431
ZFA2            QPSRP-SPP-----PVDKELQDAPPSDEGYWDSPTPGPEDEDSTFL----RRDGLLRDSC 526
HuA1            VRSYPGLAPGELLTPQSDQQESAPNSDEGYYDSTTPGFEDDSGEALGL-VRRDCLPRDSY 500
ZFA1            ADLLS---------PQSDRQESVPNSDEGYYDSTTPGMEEESRERP----HQERLPRDSY 455
HuA3            SKASSIDTG----TPKSEQPESVSTSDEGYYDSFSPGLEEDKKEAESPGTPAATFPRDSY 417
ZFA3            KVMTP------------KSDNQGNTSDEGYCDYVSPGYEDHSKSSFSP-VHSSKIPRDTY 419
                    .            .  :    ***** *  :** *:..            : **:

HuA2            SGDALYDLYADPDGSPATLPGG-------------------------------------- 453
ZFA2            SGDALYDLYDPDSPSAAGSDDD-------------------------------------- 548
HuA1            SGDALYEFYEPDDSLENSPPGDDCLYDLHGRSSEMFDPFLNFEPFLSSRPPGAMETEEER 560
ZFA1            SGDALYELFEPDDRLLSPSLPP-------------------------------------- 477
HuA3            SGDALYELFHDPSEGPLGPSPD-------------------------------------- 439
ZFA3            SGDALYELFCDPSDAEITPICD-------------------------------------- 441
                ******:::   .

HuA2            ------------------------------------------------------------
ZFA2            ------------------------------------------------------------
HuA1            LVTIQKQLLYWELRREQLEAQEARAREAHAREAHAREAYTREAYGREAYAREAHTWEAHG 620
ZFA1            ------------------------------------------------------------
HuA3            ------------------------------------------------------------
ZFA3            ------------------------------------------------------------


HuA2            -----------------------KDNEETSS----LSRLKP-----VSPGTITCPLRTPG 481
ZFA2            -----------------------ASSPTKSAGDLKMNLPSP-----KCSSSATSSFRSMK 580
HuA1            REARTREAQAREVRCRETQVRETQARQEKPVLEYQMRPLGPSVMGLAAGVSGTSQISHRG 680
ZFA1            ----------------------KDAHSFVGAPLQADKSPTNPLYSLASTAIETGAMETEE 515
HuA3            ----------------------DDLCVSESLSGPALGTPLSICSFRVGAEENLAPAPGPD 477
ZFA3            ----------------------DEIDLTDSIVGQCSDLPLSMYSFRVGAEENLAPSLARD 479
```

```
HuA2           ---SLLKDSKIPISIKHLTNLPSS------------------------------------ 502
ZFA2           GSTSLPRDSKIPISVRQTPPSHSSSQGALSSN---------------------------- 612
HuA1           ITSAFPTTASSEPDWRDFRPLEKRYEGTCSKKDQSTCLMQLFQSDAMFEPDMQEANFGGS 740
ZFA1           ERLSKIQHALLCCELQNLRSPSKNQLLFHSDCFYDD------------------------ 551
HuA3           LLSQGFLQSSWKGKECLLKLCDTELAITMGIVSWLR------------------------ 513
ZFA3           FVGQELLESKWMGKDCLLKLCDTEISLAMGVVNWLN------------------------ 515
                       :    .        .

HuA2           --------HPVVHQQPSRSEMP-RTKIPVSKVLVRRVSNRGLAGTTIRATACHDSAKKL- 552
ZFA2           -------LSPTSTTPPKKTDAPPRTKIPVSKVPVRRSGGKSTSTSQSRK----------- 654
HuA1           PRRAYPTYSPPEDPEEEEVEKEGNATVSFSQALVEFTSNGNLFSSMSCSSDSDSSFTQNL 800
ZFA1           -------SNLPVDDSKQDLQEVINQRYPQSPPRSQAVKEGVPRIRGQVQESSLFAPCADS 604
HuA3           -----RGPTPRAPPTPGQPAAPPGSQGAPRAPTEKLGGREGLASDAGGATVCSAPSRQEL 568
ZFA3           ------QKTDKSNPSELKSSQTSGEEGGDLCLRCKSKSERVRRPPRRVRTVNSIRDAAKF 569
                                                 .

HuA2           ------------------------------------------------------------
ZFA2           ------------------------------------------------------------
HuA1           PELPPMVTFDIADVERDGEGKCEENPEFHNDEDLAASLEAFELGYYHKHAFNNYHSRFYQ 860
ZFA1           VLNPQVIETTRPQPQSDDQGSLRPSRGCSQSQEELMVCFSQALVDFTKNTRLYRNSTESL 664
HuA3           WAHPGTTGLLAGESKALG-GATQGTGTLSRDASREEETRGHSEGLFSSMESAATSTTDTS 627
ZFA3           KEPSQRGVRVLPSKCDTNTVMSSLDSPHSQPNTPTSGVCFRIFNIGSPMTPGGDLQSPVV 629


HuA2           ------------------------------------------------------------
ZFA2           ------------------------------------------------------------
HuA1           GLPWGVSSLPRYLGLPGLHPRPPPAAMALNRRSRSLDTAETLEMELSNSHLVQGYLESDE 920
ZFA1           DGSESSSPFGPSLRALPAIVTFDVVDMENEGECEQQTDLAEEEEELASPYEPFEDDGCYL 724
HuA3           GKNKAPVPSTWPCSQKEPGPPGVLGCFRGPWRPGHGGDTLDAEPMLAG------------ 675
ZFA3           SSPGSGTRSLFVLAINKESLCESCKSSLKNGAKDLHLCRSCMSLIEHIKTSDLWARSSFP 689


HuA2           ------------------------------------------------------------
ZFA2           ------------------------------------------------------------
HuA1           LQAQQEDSDEEDEEEEEGEWSRDSPLSLYTEPPGAYDWPAWAPCPLPVGPGPAWISPNQL 980
ZFA1           QQDAFAECDQRTFDAYEQSLLLSNAWGIASLPRHLSLGRPCPPVPAPLALNRRSRSLDTD 784
HuA3           ------------------CVARVAALKISSNEQPPAAWPPRQDMGSGLFGQRWARGPDML 717
ZFA3           KSTLTPQPITQDLLSPASTCGIGSDISIASLVEQCASQFSSMKINMTQAHPREIRDAVVP 749


HuA2           ------------------------------------------------------------
ZFA2           ------------------------------------------------------------
HuA1           DRPSSQSPYRQATCCIPPMTMSISLSVPESRAPGESGPQLARPSHLHLPMGPCYNLQPQA 1040
ZFA1           SLEFQTSEIYTSVTKYDSKGTAFSQSRTVDCNDMDFPRQPCRITVDSWRRGYRQNFDSSN 844
HuA3           EQKQSSSSPSMTTIHGLPYSASTQDQRCRDR-----------VQDLSWLRVEPTGLGVQA 766
ZFA3           EQVVKRNKDHSQKYLKSTHKRRPVAATEKGLHARHRLSRSSFSEDVKRSLLDAGGLGFVS 809


HuA2           ------------------------------------------------------------
ZFA2           ------------------------------------------------------------
HuA1           SQSMRARPRDVLL-PVDEPSCSSSSGGFSPSPLPQAKPVGITHGIPQLP--RVRPEHPQP 1097
ZFA1           ASQQELKLPHLSQ-STVRPSHLPLKNNCRSRNLPAATRVDGEGEILFGGGDALYPCSYPP 903
HuA3           WASVEDQPLQLST-EAVEQVAHGSQLDSEPRSAPAARWSSQGHHPESLGLTLNSQQEGGV 825
ZFA3           TSPSNDLVLETYTPCTVESVDTDVSQATRPTSLPLVASSEFSCREGLMKIEGGNSAKKTN 869


HuA2           --------------------------------------
ZFA2           --------------------------------------
HuA1           QPTHYGPSSLDLSKERAEQGASLATSYSSTAMNGNLAK 1135
ZFA1           MGTQWKNRPVGVTQGVPHLRSEQSADHQEITMKNRR-- 939
HuA3           SASAPECRCSLLAREGLLCGQPEVGASGPAMAEPHL-- 861
ZFA3           KSRHRKKSAVNHEGSCSGFPGDRKVERRSRMKKGNV-- 905
```

B

	ZFAmer1	ZFAmer2	ZFAmer3
HumanAmer1	29%		
HumanAmer2		32%	
HumanAmer3			21%

Figure SM5. A. Multiple alignment of Amer proteins from human and zebrafish. Residues highlighted in red show a high degree of similarity and correspond to conserved block sequences described in Figure 1 and Figure SM1 (B1 to B6). Alignment was performed using clustalW. **B.** Similarity percentage between human and zebrafish Amer proteins. Abbreviations are as described in Table 1.

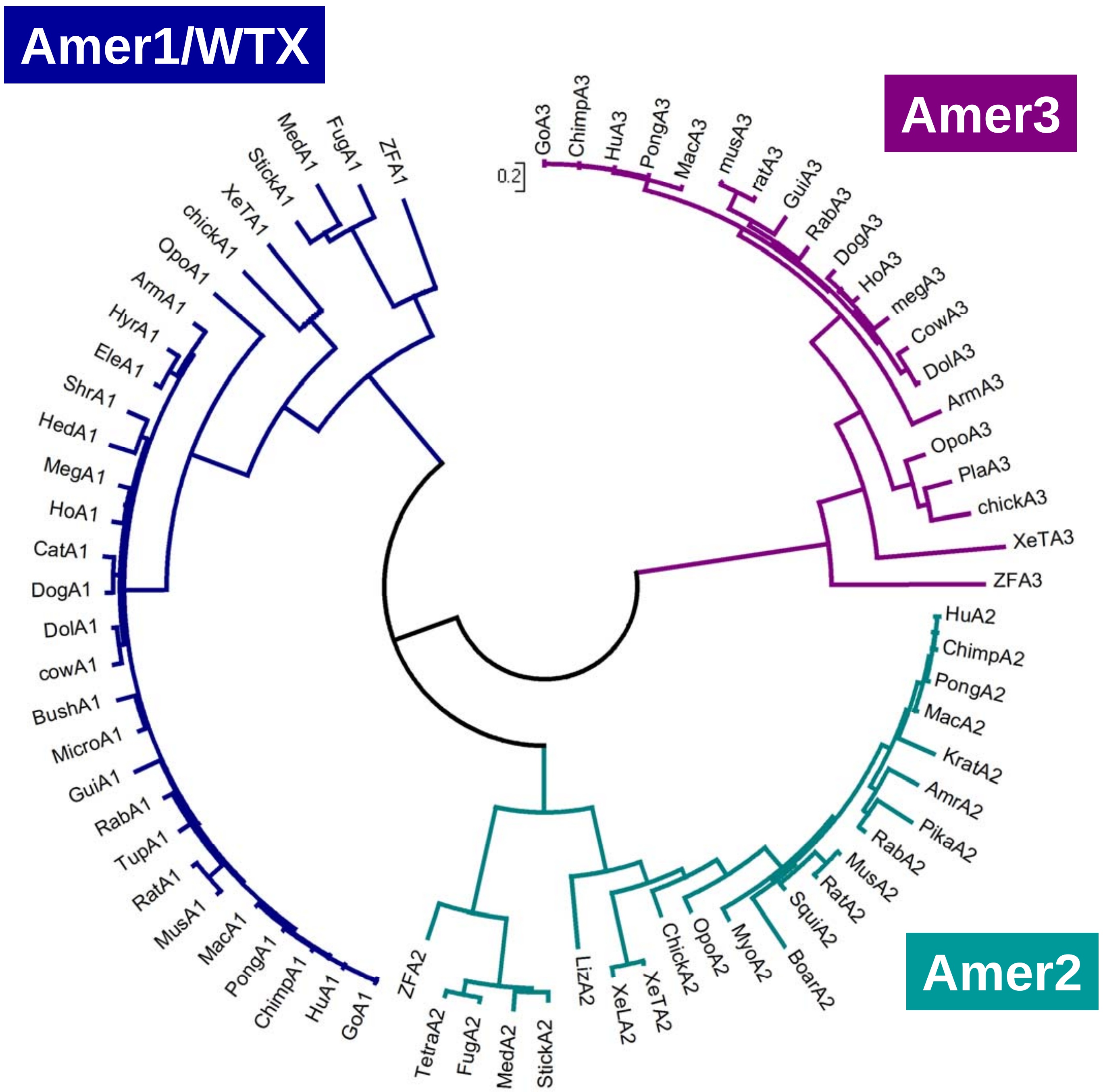

Figure SM6. Phylogenetic analysis with a broader array of Amer proteins. The phylogenetic tree has been generated with 73 sequences from 40 species by the Bayesian Inference method. Amino acid sequences overlapping the region carrying the six conserved block domains of Wtx/Amer1, Amer2 and Amer 3 were used to make the alignment (see Figure SM8 (additional data file 5). Bayesian tree was estimated under the JTT + I + G model (1 MrBayes run of 2,000,000 generations; 1,893,000 generation burn-in). Abbreviations of taxa are described in additional data file 2 (Table 1). The tree obtained with the Maximum Likelihood (ML) method displayed a similar topology (data not shown).
