# Supplementary material for: The WTX/AMER1 gene family: evolution, signature and function
Source: BMC Evol Biol. 2010 Sep 15;10:280. doi: 10.1186/1471-2148-10-280 (PMC2949870; doi:10.1186/1471-2148-10-280)
Supplement: Additional file 4 — Additional figure SM7. Figure SM7 contains multiple alignment performed with Wtx/Amer1, Amer2 and Amer3 from each class of the vertebrate subphylum using the whole sequence (28 sequences in total). [file 1471-2148-10-280-S4.PDF]

```
                  10        20        30        40        50        60        70        80        90       100
          ....|....|....|....|....|....|....|....|....|....|....|....|....|....|....|....|....|....|....|....|
MusA1     ----------------------------------------------------------------------------------------------------
HuA1      ----------------------------------------------------------------------------------------------------
ChimpA1   ----------------------------------------------------------------------------------------------------
RatA1     ----------------------------------------------------------------------------------------------------
ArmA1     ----------------------------------------------------------------------------------------------------
OpoA1     ----------------------------------------------------------------------------------------------------
ChickA1   ----------------------------------------------------------------------------------------------------
XeTA1     MGDQGLVLVPIFVDNLRPIDKNAECKLPVGWHTWRFAEVSSQGNFRDFGKSRRHPVEWHFGEISRLRQGRLVVQRLISPCVTALRYEITSSEQLVMVGTC
ZFA1      ----------------------------------------------------------------------------------------------------
MusA2     ----------------------------------------------------------------------------------------------------
HuA2      ----------------------------------------------------------------------------------------------------
ChimpA2   ----------------------------------------------------------------------------------------------------
RatA2     ----------------------------------------------------------------------------------------------------
ArmA2     ----------------------------------------------------------------------------------------------------
OpoA2     ----------------------------------------------------------------------------------------------------
ChickA2   ----------------------------------------------------------------------------------------------------
XeTA2     ----------------------------------------------------------------------------------------------------
XeLA2     ----------------------------------------------------------------------------------------------------
ZFA2      ----------------------------------------------------------------------------------------------------
HuA3      ----------------------------------------------------------------------------------------------------
ChimpA3   ----------------------------------------------------------------------------------------------------
MusA3     ----------------------------------------------------------------------------------------------------
RatA3     ----------------------------------------------------------------------------------------------------
ArmA3     ----------------------------------------------------------------------------------------------------
OpoA3     ----------------------------------------------------------------------------------------------------
ChickA3   ----------------------------------------------------------------------------------------------------
XeTA3     ----------------------------------------------------------------------------------------------------
ZFA3      ----------------------------------------------------------------------------------------------------

                 110       120       130       140       150       160       170       180       190       200
          ....|....|....|....|....|....|....|....|....|....|....|....|....|....|....|....|....|....|....|....|
MusA1     ----------------------------------------------------------MESQQD----EAVQTKGASTS------SDAQDQGAEKGAKNK
HuA1      ----------------------------------------------------------METQKD----EAAQAKGAAAS------GSTREQTAEKGAKNK
ChimpA1   ----------------------------------------------------------METQKD----EAAQAKGAATS------GSTREQTAEKGAKNK
RatA1     ----------------------------------------------------------MEIQQD----ETVQIKRTSTS------SDAQDQGAEKGAKNK
ArmA1     ----------------------------------------------------------METQKD----DASQAKGAAAS------GTIQGQGTEKGAKHK
OpoA1     ----------------------------------------------------------METYRDAHSEELLKTKGKAAV------GEPQGKGAESRAPGQ
ChickA1   ----------------------------------------------------------METGCT---EEAARTQSQSAACRQCKGGEQQPEENVERLSQR
XeTA1     EFTKHLLYVPGKDTMSWAEIAYPWEHVHLQPGTYAESTPARGTVCLQLRIPVKCITFAMESRQS----GQEKHTGTKTS---LSVCDSHDSILSDGDSGK
ZFA1      -------------------------------------------------------------------------------------MEIATRCEVGAMRGP
MusA2     ---------------------------------------------------------------------------------------------------M
HuA2      ---------------------------------------------------------METSRSRGGGGAVSERGGAGASVGVCRRKAEAGAGTGTLAADM
ChimpA2   ---------------------------------------------------------METSRSRGGGGAVSERGGAGASVGVCRRKAEAGAGTGTLAADM
RatA2     ---------------------------------------------------------------------------------------------------M
ArmA2     ------------------------------------------------------MATSGRSSQRRSREAGRERPGARAAEGGCGRKEEAGP--GTPSADM
OpoA2     ---------------------------------------------------------------------------------------------------M
ChickA2   ---------------------------------------------------------------------------------------------------M
XeTA2     --------------------------------------------------------------------GAEKRLPVRNSASGICGGLQEEGGRIKEKANM
XeLA2     ---------------------------------------------------------------------------------------QEEGGRIKEKANM
ZFA2      ---------------------------------------------------------------------------------------------------M
HuA3      ---------------------------------------------------------------------MELKRGKTFIKSSLQVSHE-KPPDPAAVAAA
ChimpA3   ---------------------------------------------------------------------MELKRGKTFIKSSLQVSHE-KPPDPAAVAAA
MusA3     ---------------------------------------------------------------------MELRRGKTFIKSSVQISHE-KLIDS----PA
RatA3     ---------------------------------------------------------------------MELRRGKTFIKSSVQISHE-KLIDS----PA
ArmA3     --------------------------------------------------------------------------------------QD-KPPDP-ATTLA
OpoA3     ---------------------------------------------------------------------MELRRGKTFIKSSLQISHE-KPTVAEAALRG
ChickA3   ---------------------------------------------------------------------MELKRGKTFIKSSAQHERV-PPAHTAPANRD
XeTA3     ---------------------------------------------------------------------MELLRGKTFIKSYTQNPPENLPCIGVADKKI
ZFA3      -----------------------------------------------------------------------------------MELSKSEDSSDNRKRSN
```

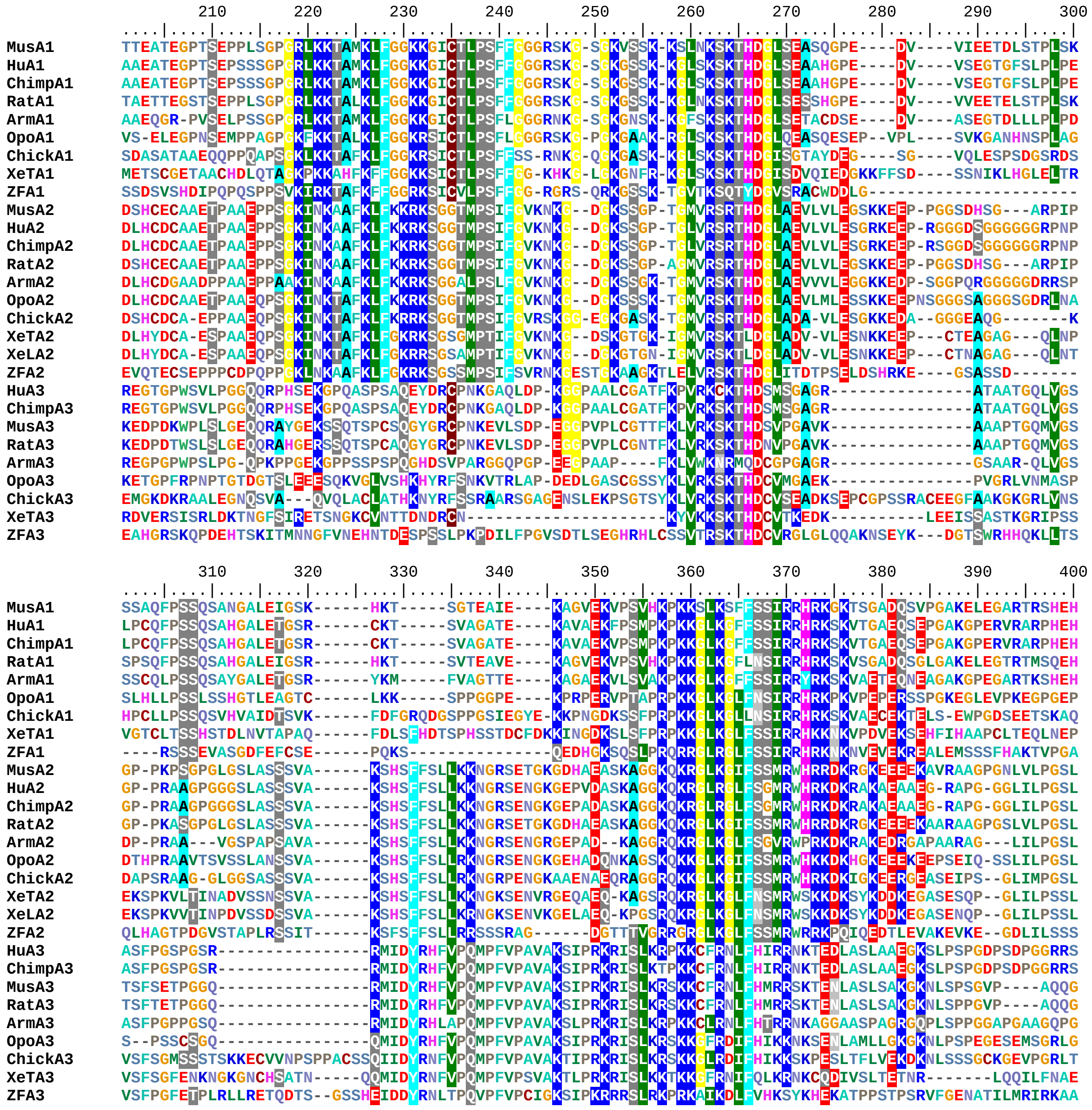
210 220 230 240 250 260 270 280 290 300
MusA1
HuA1
ChimpA1
RatA1
ArmA1
OpoA1
ChickA1
XeTA1
ZFA1
MusA2
HuA2
ChimpA2
RatA2
ArmA2
OpoA2
ChickA2
XeTA2
XeLA2
ZFA2
HuA3
ChimpA3
MusA3
RatA3
ArmA3
OpoA3
ChickA3
XeTA3
ZFA3
310 320 330 340 350 360 370 380 390 400

```
                  410       420       430       440       450       460       470       480       490       500
          ....|....|....|....|....|....|....|....|....|....|....|....|....|....|....|....|....|....|....|....|
MusA1     VSSISLPSS-EEIFRDTRKENAKPQDAPGPKMSPAQVHFSPTTEKAACKNPEK-------LTRTCASEFMQPKPVLEGGSLEEPHTSETEGKVVA-----
HuA1      VSSAPQVPCFEETFQAPRKENANPQDAPGPKVSPTPEPSPPATEKMACKDPEK-------PMEACASAHVQPKPAPEASSLEEPHSPETGEKVVA-----
ChimpA1   MSSAPQVPCFEETFQAPRKENANPQDAPGPKVSPTPEPSPPATEKMACKDPEK-------PMEACASAHVQPKPAPEASSLEEPHSPETGEKVVA-----
RatA1     VSSISLPSS-EEIFRDAIKENTKPQDAPGPKMSPAREHFSLTTEKTACKNPEK-------LTMTCASEFMQPKPILEASSLEEPHTSETEGKVVT-----
ArmA1     VSSAPLLHS-DDTLQPPNKEDAKPLDAPEPKVSQPPDPSLPATAKTAYKDPEK-------SKEACASALLQPKPASKASSPEECHSPETGEKVVV-----
OpoA1     LSLAPQPISRDECLP-LLPSNAKSIELQGP----EPDPPLPVVGEMSQENPEQ-------FSDASISVPPPLEPNPEPGGPGDSSGPEGRGDEEE-----
ChickA1   GTKAETPGTVEEGVPGSVPLAAACPGSSEDNCLVRTVADFGEAAEPDWLQADKG------SCEGDVVAVPGGKDDLDAKSEVDAVVYTESNYSHLPVALH
XeTA1     ELENMQEGNEQTKIECLPTEVSQEPSLSTAVGTEETESSECLSDVAPIPSVELP------VEPDHLNSLNKDHSLDAQLDSAVICSSDDGIFSEIVSP--
ZFA1      LPSVSDRGDYHGDSQGEELVPDVPNQTTGSECELPLAATECTIDVTLVPEKRRSRVEMDKRRRAEKEGIGEDEKTGRQEGLMTYHQPLSAESELDRLAEQ
MusA2     TASLECVKEEPPRAARRPDSPGQDASRHAAGEPAGGEQAPASAESAPERICLEAGSPTGSGDQSSRGEDAEGHRREEKPGAALESGAGEVQAAEDASKTG
HuA2      TASLECVKEETPRAAREPEEPSQDAPRDPAG---------------------------------------------------------------------
ChimpA2   TASLECVKEETPRAAREPEEPSQDAPRDPAG---------------------------------------------------------------------
RatA2     TASLECVKEEPPRTARRPDSPGQDAPRHAAGEPAGGEQAPASAERAPERTCLEASSPTGPGDQSSRGEDAEGHRRAEKPGAALESGAGEVQAAEDASRTG
ArmA2     TASLDFDS--------------------LTG---------------------------------------------------------------------
OpoA2     TASLECIKEEIPKPLCEPENPTKEIRKEPSCEFRGGEEVNASVDKPEVRNSGESLSPVLHNNKNTQREDATGHRHEERHRESGEPGTGEIRTAQDTARTG
ChickA2   TASLECIKEETPKPLSETPNGAGDTGVESQQEKRGGDACVSAEEPQ-----AGGGESRDSKTPPGEDPAAAARRLEELCGERPDPGAGEVGTAKDAAITG
XeTA2     TASLECIKEETQKPLCEKGKSTEDIPADVPLAEHSGDVNTSAEENS--LKASEESPCSALITEQPQLEDAPLAQLQENLCQLPQPEVETLQNNKDEHVT-
XeLA2     TASLECIKEETQKPLCEKEKSEEDIPADVPSVEHSGDVNTSAEENP--LNGCVESPCPALITKEPQLEDPPVIQQVDNLYQLPDPEVETLPDNKDEDVTG
ZFA2      SGSVKTEKDMTLTLEPLPQVFEESPLPGDSDKWKVASMQEIQGTNEVECGNCGPSVSQQHTVTEESPAPSPLRVQTGGFQNHKHSSSTHLSSIPTCALTP
HuA3      KAFLPPGEGPGLDGLCQDLLDSELLADASFGLCR------------------------------------------------------------------
ChimpA3   KAFLPPGEGPGLDGLCQDLLDSELLADASFGLCR------------------------------------------------------------------
MusA3     TAFLSMGEGLGLDSLCQDLSDSEFLHDSPFDLCS------------------------------------------------------------------
RatA3     KPFFSMGEGLGLDSLCQDLSDSEFLHDSPFDLCS------------------------------------------------------------------
ArmA3     QAFCAEGDAPGPDGLSQDLSDSELPAEAALDLCR------------------------------------------------------------------
OpoA3     K----PGETLAADSLAQDLSDSEIQSDSSYDYCG------------------------------------------------------------------
ChickA3   KSLFNTGETFAADCLSQDCSDNELQSDSSYDCCN------------------------------------------------------------------
XeTA3     EGKMDSREIHPDELSVYEFSDNELNIDP-IDRYH------------------------------------------------------------------
ZFA3      RHRECSTAGSRCNDELSETPSDSSSESGPN----------------------------------------------------------------------

                  510       520       530       540       550       560       570       580       590       600
          ....|....|....|....|....|....|....|....|....|....|....|....|....|....|....|....|....|....|....|....|
MusA1     --------------------------GEVNPPNGPVGDQLSLLFGDVTSLKSFDSLTGCGDIIAEQDMDSMTDS--------------------------
HuA1      --------------------------GEVNPPNGPVGDPLSLLFGDVTSLKSFDSLTGCGDIIAEQDMDSMTDS--------------------------
ChimpA1   --------------------------GEVNPPNGPVGDPLSLLFGDVTSLKSFDSLTGCGDIIAEQDMDSMTDS--------------------------
RatA1     --------------------------GDANPPNGPVGDQLSLLFGDVTSLKSFDSLTGCGDIIAEQDMDSMTDS--------------------------
ArmA1     --------------------------GEVNLPSGPVGDQLSLLFGDVTSLKSFDSLTGCGDIIAEQDMDSMTDS--------------------------
OpoA1     --------------------------GEPS--LGLSGDQLSLLFGDVTSLKSFDSLTGCGDIIAEQDVDSITES--------------------------
ChickA1   -----------------------PDLADNDPPSLHSGDQLSLLFGDVTSLKSFDSLTGCGDIIAEPDILSIAES--------------------------
XeTA1     --------------------------DVIDKVPLSSGDQISLIFEDVSSLKSFDSLTGCGDIIADQDIDTVSDN--------------------------
ZFA1      -----------------------NVDVPDGEPPVASCSSENLVFGDVSSLKSFDSLTGCGDIIADQDDVSVAES--------------------------
MusA2     DVPIKTVPLVDSEGGSGR-ASAVPDPSSVDPPSDPSADRICLMFSDVTSLKSFDSLTGCGDIIADP-EEEAGPS--------------------------
HuA2      ----------------------------------------------------------CGDIIADQ-EEEAGPS--------------------------
ChimpA2   ----------------------------------------------------------CGDIIADQ-EEEAGPS--------------------------
RatA2     DVPIKTVPLVDSEGGSGR-ASAVPDPSSVDPPSDPSADRICLMFSDVTSLKSFDSLTGCGDIIADP-EEEAGPS--------------------------
ArmA2     ----------------------------------------------------------CGDIIAVLQEEEAGAS--------------------------
OpoA2     DVPIKTIPLVEPGCNSGP-DTAVPDPSSIDPPSEPSIDRICLMFADVTSLKSFDSLTGCGDIIADQ-EDEAGAS--------------------------
ChickA2   DIPITTIPPVEPHCDSGQETAAAPDPSSVDPPSEQSIDRICLMFADVTSLKSFDSLTGCGDIIADQEEDVGGGSGG------------------------
XeTA2     ---------------------------------------------------------GCGDVIADQ-DDDGGSSM-------------------------
XeLA2     DIPVNTVSIVEPECDVGQ-EIAAPDPTTVDPPSEPSFDRICLMLADVTSLKSFDSLTGCGDVIADQ-DDDGGSSK-------------------------
ZFA2      ----------------------PMEHSTADPQSEQSVDRLCSMFTDVTSLKSFDSLTGCGDIIADPEEDSGNGGSATSSGTGSSSGGCMGRRLSGAGTNS
HuA3      -----------------------------------------ALCEDVASLQSFDSLTGCGEVFADESSVPSLELNE------------------------
ChimpA3   -----------------------------------------ALCEDVASLQSFDSLTGCGEVFADESSVPSLELNE------------------------
MusA3     -----------------------------------------ALCEDVASLKSFDSLTGCGEIFADGSSVPSVELKD------------------------
RatA3     -----------------------------------------ALCEDVASLKSFDSLTGCGEIFADGSSVPSVELKD------------------------
ArmA3     -----------------------------------------ALCEDVASLKSFDSLTGCGEIFADESSVPSLELGL------------------------
OpoA3     -----------------------------------------ALCEDVASLKSFDSLTGCGEIFADEVSA-SLELEG------------------------
ChickA3   -----------------------------------------ALCEDVASLKSFDSLTGCGEIFADESSA-HLELES------------------------
XeTA3     -----------------------------------------RFCEDVASLKSFDSLTGCGEIFADENHT--FLGTD------------------------
ZFA3      ------------------------------------------VCEDAVSLKSFGSQAGCGEIFADDLVSPDGVLNS------------------------
```

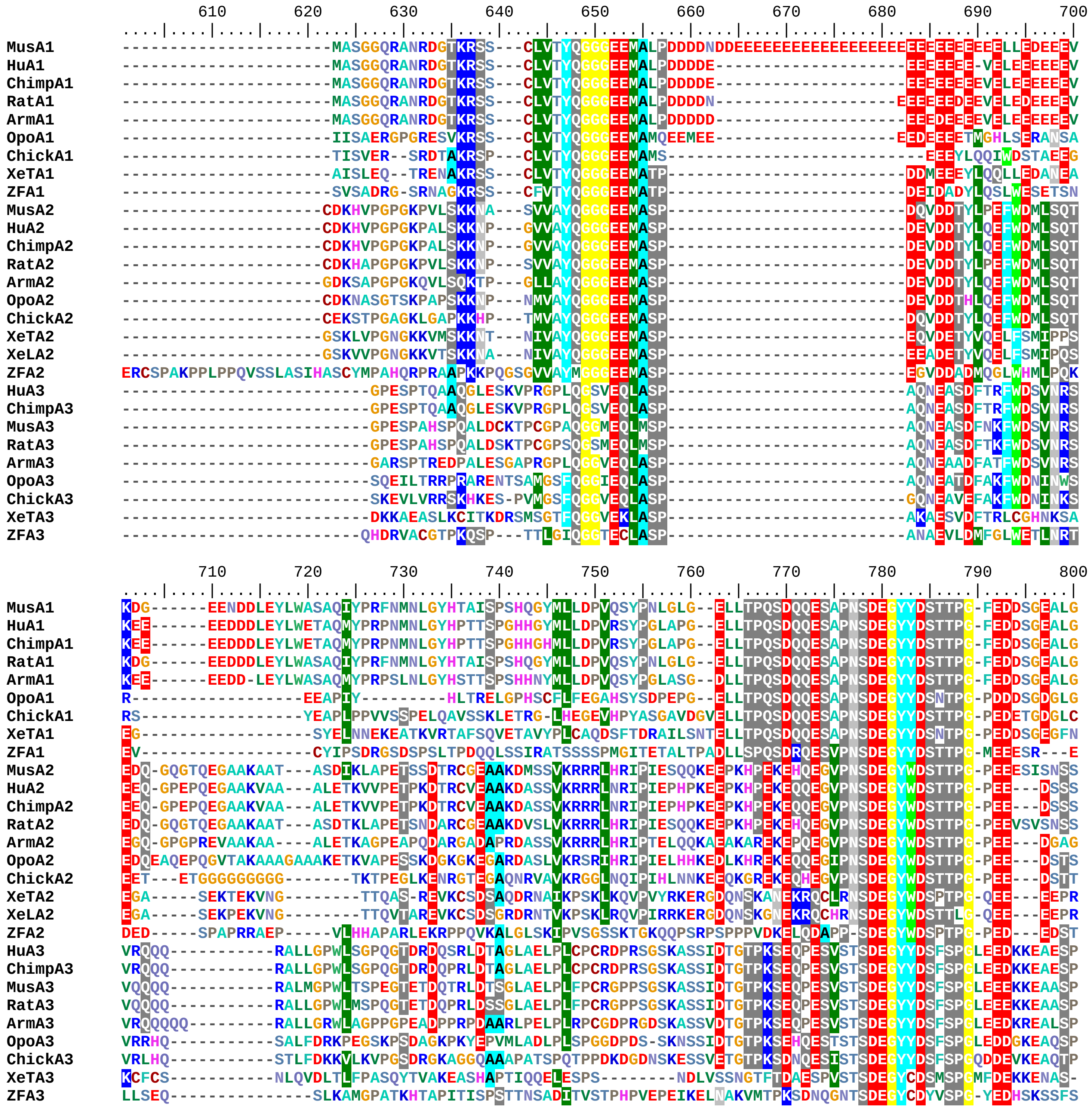

610 620 630 640 650 660 670 680 690 700
MusA1
HuA1
ChimpA1
RatA1
ArmA1
OpoA1
ChickA1
XeTA1
ZFA1
MusA2
HuA2
ChimpA2
RatA2
ArmA2
OpoA2
ChickA2
XeTA2
XeLA2
ZFA2
HuA3
ChimpA3
MusA3
RatA3
ArmA3
OpoA3
ChickA3
XeTA3
ZFA3
710 720 730 740 750 760 770 780 790 800
MusA1
HuA1
ChimpA1
RatA1
ArmA1
OpoA1
ChickA1
XeTA1
ZFA1
MusA2
HuA2
ChimpA2
RatA2
ArmA2
OpoA2
ChickA2
XeTA2
XeLA2
ZFA2
HuA3
ChimpA3
MusA3
RatA3
ArmA3
OpoA3
ChickA3
XeTA3
ZFA3

```
                 810       820       830       840       850       860       870       880       890       900
             ....|....|....|....|....|....|....|....|....|....|....|....|....|....|....|....|....|....|....|....|
MusA1        LAHRDCLPRDSYSGDALYEFYEPDDSLEHSPPGDDCLYDLRGRNSEMLDPFLNLEPFSS-RPP-------GAMETEEERLVTIQKQLLYWELRREQREAQ
HuA1         LVRRDCLPRDSYSGDALYEFYEPDDSLENSPPGDDCLYDLHGRSSEMFDPFLNFEPFLSSRPP-------GAMETEEERLVTIQKQLLYWELRREQLEAQ
ChimpA1      LVRRDCLPRDSYSGDALYEFYEPDDSLENSPPGDDCLYDLHGRSSEMFVPFLNFEPFSSSRPP-------GAMETEEERLVTIQKQLLYWELRREQLEAQ
RatA1        LVHRDCLPRDSYSGDALYEFYEPDDSLEHSPPGDDCLYDLRGRNSEMLDPFLNLEPFSS-RPP-------GAMETEEERLVTIQKQLLYWELRREQREAQ
ArmA1        LIHKDCLPRDSYSGDALYEFYEPVDSLENSPPGDDCLYDLHGCSSEMFDRFLDFEPFSSSRPP-------GAMETEEERLVTIQKQLLYWELRREQLEAR
OpoA1        PGKKDQLPRDSYSGDALYEFFEPCDSLTGSPPGDEGLFDPPGPGPELFDNFLSFKPFPSSKGPDPS---LGTMETEEERLVTIQRQLLYWELRRKRQEPQ
ChickA1      EIKKDRLPRDSYSGDALYEFYEPDDTLMSPSHGEQSLFESKVSHPEIFSYFLDFCLPPEKSLIQMMDQKRGVMETEEERLAAIQKELLFWELQREPGLKR
XeTA1        --HKERLPRDSYSGDALYEFYEPEDSLMSPAPGGESLYETKTPCSEIFDQFFDFSLTSNNDLIQDHVHKTCATETEEERLAVIQKQLLFWERQREATLKG
ZFA1         RPHQERLPRDSYSGDALYELFEPDDRLLSPSLPPKDAHSFVGAPLQADKSPTNPLYSLASTAIET-----GAMETEEERLSKIQHALLCCELQN------
MusA2        SSKKVVIPRDSDSGDALCDLYVEPEASPATLP---------ATEDPPCLSRLKPVSPGTITCP-----LRTPGSLLKDSKIPISIKHLS----------
HuA2         SGKKAGIPRDSYSGDALYDLYADPDGSPATLPG------GKDNEETSSLSRLKPVSPGTITCP-----LRTPGSLLKDSKIPISIKHLT----------
ChimpA2      SGKEAGIPRDSYSGDALYDLYADPDGSPATLPG------GKDNEETSSLSRLKPVSPGTITCP-----LRTPGSLLKDSKIPISIKHLT----------
RatA2        SSKKVVIPRDSNSGDVLCDLYVEPEASPAALP---------ATEDPPCLSRLKPVSPGTITCP-----LRTPGSLLKDSKIPISIKHLS----------
ArmA2        SGQKAGLPRDSCSGDALYDLDAEPDDGAPGLP---------ATEETSCLSRLKPVSPVTITCP-----LRTPGSLLKDSRIPISIKHLA----------
OpoA2        TIQKEGIPRDSYSGDALYDLYTDPEENPAGLP---------SDEEVSGLSRSKPVSPITITCP-----LKTPGSLLKDSKIPISIKHLA----------
ChickA2      SIQKETLPRDSYSGDALYDLYAEPDENPPGGP---------PEEEVTCMPRSKPVSPITTTCS-----LKTPSSTVKDSKIPISIKHLA----------
XeTA2        SVGKQALARDSCSGDALYDLYTDPDESIAKAQ---------VEEPPLSHSHSKPLSPVTTSCP-----VKTASSN-KESKIPISIKHLP----------
XeLA2        SVGKQALPRDSCSGDALYDLYTDPDESIPKAQ---------AEEPPVSHSHSKPLSPVTASCP-----VKTASSN-KESKIPISIKHLP----------
ZFA2         FLRRDGLLRDSCSGDALYDLYDPDSPSAAGSDDDASSPTKSAGDLKMNLPSPKCSSSATSSFRS----MKGNTSLPRDSKIPISVRQTPP----------
HuA3         GTPAATFPRDSYSGDALYELFHDPSEGPLGPSPDDDLCVSESLSGPALGTPLSICSFRVGAEE-------NLAPAPGPDLLSQGFLQSSWK---------
ChimpA3      GTPAATFPRDSYSGDALYELFHDPSEGPVGPSPDDDLCVSESLSGPALGTPLSICSFRVGAEE-------NLAPAPGPDLLSQGFLQSSWK---------
MusA3        GTPAATFPRDSYSGDALYELFYDPSEAPVGPILDDD-CVSESLSGPALGTPLSMCSFRVGAEE-------NLAPAPGPDLLSQGFLQSTWK---------
RatA3        ATPAAAFPRDSYSGDALYELFYDPSEAPVGPILDDD-CVSESLSGPALGTPLSMCSFRVGAEE-------NLAPAPGPDLLSQGFLQSTWK---------
ArmA3        GTPTSTFPRDSYSGDALFELFCDPSESPAGPGLDDDLCVSESLSGPALGTPLSMCSFCVGAEE-------NLAPPPGPDLLSQS-LQSSWE---------
OpoA3        GVPSGRFPRDSYSGDALYELFYDPSEAKISPILDDDLCVSENLSEQAVEIPLSIYSFHVGAEE-------NLAPQPTLDIISQSFLHSTWK---------
ChickA3      GVP-GRFPRDSYSGDALYELFYDPNEVKINPVMDDDLCTSESVSEQAIEIPLSIYSFHVGAEE-------NMASQPGIDIISQGFFHSTWK---------
XeTA3        ---LPTYPRDSYSGDALFELFYNSNETTPCSALDSALSISG----HSTDNPQSMYSFCVGSEE-------NMATHIASDLVEDFGLQGSWK---------
ZFA3         PVHSSKIPRDTYSGDALYELFCDPSDAEITPICDDEIDLTDSIVGQCSDLPLSMYSFRVGAEEN-------LAPSLARDFVGQELLESKWMG--------

                 910       920       930       940       950       960       970       980       990       1000
             ....|....|....|....|....|....|....|....|....|....|....|....|....|....|....|....|....|....|....|
MusA1        EACAREAHAREAYARDTHTRESYG---------------RNVRARETQALEAHSQEGRVQETKVRQEKPALEYQMRPLGPSVMGLVAGTSG--GSQTSHR
HuA1         EARAREAHAREAHAREAYTREAYGREAYAREAHTWEAHGREARTREAQAREVRCRETQVRETQARQEKPVLEYQMRPLGPSVMGLAAGVSG--TSQISHR
ChimpA1      EARAREAHAREAHAREAYTREAYGREAYARETHTWEAHGREARTREAQAREVRSRETQVRETQARQEKPVLEYQMRPLGPSVMGLAAGVSG--TSQISHR
RatA1        EACAREAHAREAYSRDTHTRESYG---------------KNTRARETQALEVHSQEGRVQETKVRQEKPALEYQMRPLGPSVMGLVPGTPG--GSQTSHR
ArmA1        EA--WEAHAREAHAREAHTREIYVQEAHAQEVHAREARAREVYTKEAHAREAWARENHVREAQIQQKNPIMEYQMRPLGPSVMGLVAGVSG--ASQASYR
OpoA1        GP------------------------------------------------------------YPQREKPSEEHHERALGP-----FRKNTG--GDPAALL
ChickA1      LDVP-------------------------------------------------------SKEKCPREKQHVECNTRAANLIGKSQSCLGSEQIASQALNR
XeTA1        MAHVN-------------------------------------------------------KGIFPKEEQNVECKNKADILCHNKDCHLNNHSLLQNISKG
ZFA1         ----------------------------------------------------------------LRSPSKNQLLFHSDCFYDDSNLPVDDS---KQDLQE
MusA2        ----------------------------------------------------------------------------------------------------
HuA2         ----------------------------------------------------------------------------------------------------
ChimpA2      ----------------------------------------------------------------------------------------------------
RatA2        ----------------------------------------------------------------------------------------------------
ArmA2        ----------------------------------------------------------------------------------------------------
OpoA2        ----------------------------------------------------------------------------------------------------
ChickA2      ----------------------------------------------------------------------------------------------------
XeTA2        ----------------------------------------------------------------------------------------------------
XeLA2        ----------------------------------------------------------------------------------------------------
ZFA2         ----------------------------------------------------------------------------------------------------
HuA3         ----------------------------------------------------------------------------------------------------
ChimpA3      ----------------------------------------------------------------------------------------------------
MusA3        ----------------------------------------------------------------------------------------------------
RatA3        ----------------------------------------------------------------------------------------------------
ArmA3        ----------------------------------------------------------------------------------------------------
OpoA3        ----------------------------------------------------------------------------------------------------
ChickA3      ----------------------------------------------------------------------------------------------------
XeTA3        ----------------------------------------------------------------------------------------------------
ZFA3         ----------------------------------------------------------------------------------------------------
```

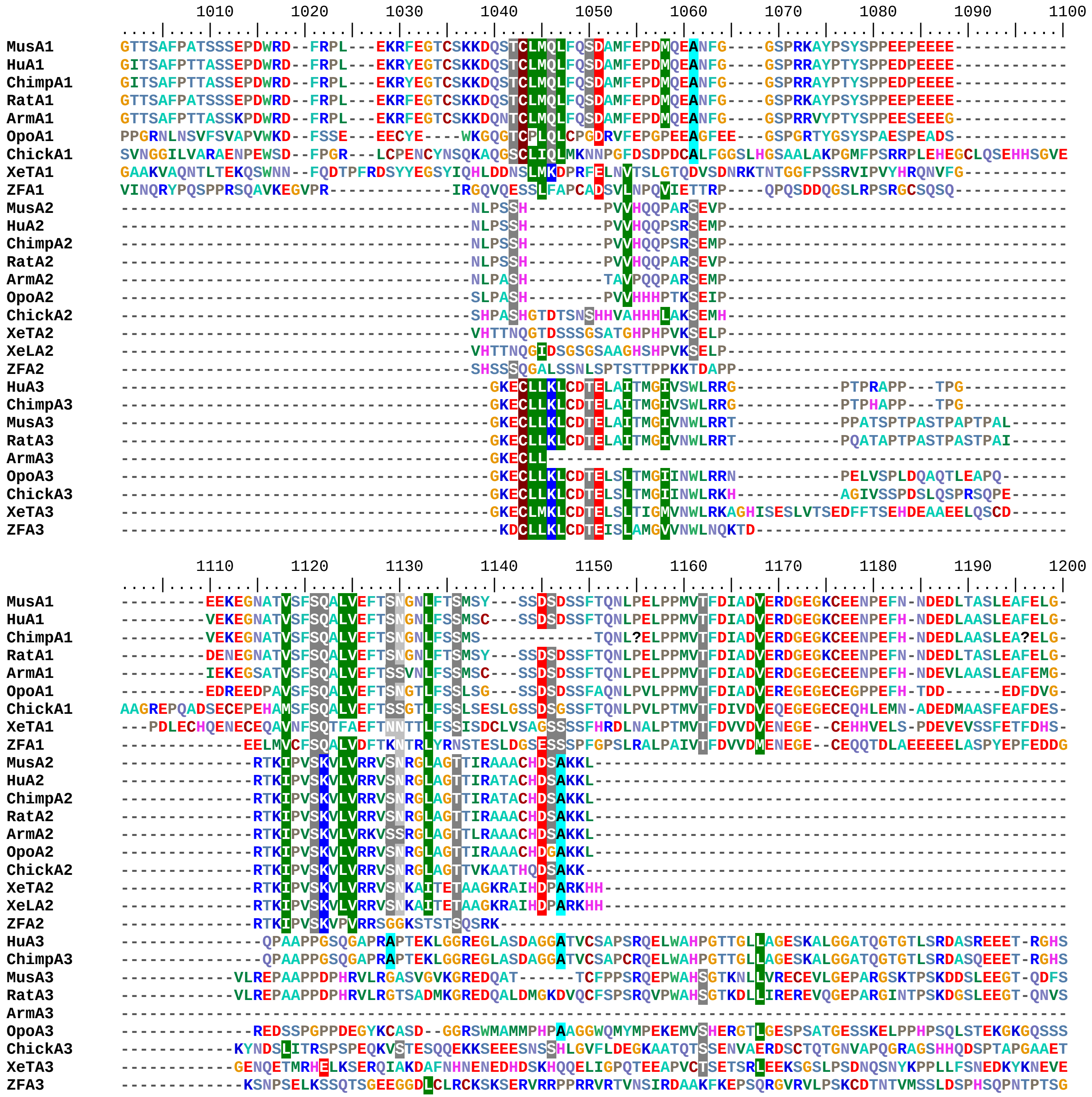
1010 1020 1030 1040 1050 1060 1070 1080 1090 1100
MusA1 GTTSAFPATSSSEPDWRD--FRPL---EKRFEGTCSKKDQSTCLMQLFQSDAMFEPDMQEANFG----GSPRKAYPSYSPPEEPEEEE-----------
HuA1 GITSAFPTTASSEPDWRD--FRPL---EKRYEGTCSKKDQSTCLMQLFQSDAMFEPDMQEANFG----GSPRRAYPTYSPPEDPEEEE-----------
ChimpA1 GITSAFPTTASSEPDWRD--FRPL---EKRYEGTCSKKDQSTCLMQLFQSDAMFEPDMQEANFG----GSPRRAYPTYSPPEDPEEEE-----------
RatA1 GTTSAFPATSSSEPDWRD--FRPL---EKRFEGTCSKKDQSTCLMQLFQSDAMFEPDMQEANFG----GSPRKAYPSYSPPEEPEEEE-----------
ArmA1 GTTSAFPTTASSKPDWRD--FRPL---EKRFEGTCSKKDQNTCLMQLFQSDAMFEPDMQEANFG----GSPRRVYPTYSPPEESEEEG-----------
OpoA1 PPGRNLNSVFSVAPVWKD--FSSE---EECYE----WKGQGTCPLQLCPGDRVFEPGPEEAGFEE---GSPGRTYGSYSPAESPEADS-----------
ChickA1 SVNGGILVARAENPEWSD--FPGR---LCPENCYNSQKAQGSCLIQLMKNNPGFDSDPDCALFGGSLHGSAALAKPGMFPSRRPLEHEGCLQSEHHSGVE
XeTA1 GAAKVAQNTLTEKQSWNN--FQDTPFRDSYYEGSYIQHLDDNSLMKDPRFELNVTSLGTQDVSDNRKTNTGGFPSSRVIPVYHRQNVFG----------
ZFA1 VINQRYPQSPPRSQAVKEGVPR------------IRGQVQESSLFAPCADSVLNPQVIETTRP----QPQSDDQGSLRPSRGCSQSQ-----------
MusA2 -------------------------------------NLPSSH--------PVVHQQPARSEVP------------------------------------
HuA2 -------------------------------------NLPSSH--------PVVHQQPSRSEMP------------------------------------
ChimpA2 -------------------------------------NLPSSH--------PVVHQQPSRSEMP------------------------------------
RatA2 -------------------------------------NLPSSH--------PVVHQQPARSEVP------------------------------------
ArmA2 -------------------------------------NLPASH--------TAVPQQPARSEMP------------------------------------
OpoA2 -------------------------------------SLPASH--------PVVHHHPTKSEIP------------------------------------
ChickA2 -------------------------------------SHPASHGTDTSNSHHVAHHHLAKSEMH------------------------------------
XeTA2 -------------------------------------VHTTNQGTDSSSGSATGHPHPVKSELP------------------------------------
XeLA2 -------------------------------------VHTTNQGIDSGSGSAAGHSHPVKSELP------------------------------------
ZFA2 -------------------------------------SHSSSQGALSSNLSPTSTTPPKKTDAPP-----------------------------------
HuA3 ---------------------------------------GKECLLKLCDTELAITMGIVSWLRRG-----------PTPRAPP---TPG-----------
ChimpA3 ---------------------------------------GKECLLKLCDTELAITMGIVSWLRRG-----------PTPHAPP---TPG-----------
MusA3 ---------------------------------------GKECLLKLCDTELAITMGIVNWLRRT-----------PPATSPTPASTPAPTPAL------
RatA3 ---------------------------------------GKECLLKLCDTELAITMGIVNWLRRT-----------PQATAPTPASTPASTPAI------
ArmA3 ---------------------------------------GKECLL---
OpoA3 ---------------------------------------GKECLLKLCDTELSLTMGIINWLRRN-----------PELVSPLDQAQTLEAPQ-------
ChickA3 ---------------------------------------GKECLLKLCDTELSLTMGIINWLRKH-----------AGIVSSPDSLQSPRSQPE------
XeTA3 ---------------------------------------GKECLMKLCDTELSLTIGMVNWLRKAGHISESLVTSEDFFTSEHDEAAEELQSCD-----
ZFA3 --KDCLLKLCDTEISLAMGVVNWLNQKTD---------------------------------
1110 1120 1130 1140 1150 1160 1170 1180 1190 1200
MusA1 ---------EEKEGNATVSFSQALVEFTSNGNLFTSMSY---SSDSDSSFTQNLPELPPMVTFDIADVERDGEGKCEENPEFN-NDEDLTASLEAFELG-
HuA1 ---------VEKEGNATVSFSQALVEFTSNGNLFSSMSC---SSDSDSSFTQNLPELPPMVTFDIADVERDGEGKCEENPEFH-NDEDLAASLEAFELG-
ChimpA1 ---------VEKEGNATVSFSQALVEFTSNGNLFSSMS------------TQNL?ELPPMVTFDIADVERDGEGKCEENPEFH-NDEDLAASLEA?ELG-
RatA1 ---------DENEGNATVSFSQALVEFTSNGNLFTSMSY---SSDSDSSFTQNLPELPPMVTFDIADVERDGEGKCEENPEFN-NDEDLTASLEAFELG-
ArmA1 ---------IEKEGSATVSFSQALVEFTSSVNLFSSMSC---SSDSDSSFTQNLPELPPMVTFDIADVERDGEGECEENPEFH-NDEVLAASLEAFEMG-
OpoA1 ---------EDREEDPAVSFSQALVEFTSNGTLFSSLSG---SSDSDSSFAQNLPVLPPMVTFDIADVEREGEGECEGPPEFH-TDD------EDFDVG-
ChickA1 AAGREPQADSECEPEHAMSFSQALVEFTSSGTLFSSLSESLGSSDSGSSFTQNLPVLPTMVTFDIVDVEQEGEGECEQHLEMN-ADEDMAASFEAFDES-
XeTA1 ---PDLECHQENECEQAVNFSQTFAEFTNNTTLFSSISDCLVSAGSSSSFHRDLNALPTMVTFDVVDVENEGE--CEHHVELS-PDEVEVSSFETFDHS-
ZFA1 -------------EELMVCFSQALVDFTKNTRLYRNSTESLDGSESSSPFGPSLRALPAIVTFDVVDMENEGE--CEQQTDLAEEEEELASPYEPFEDDG
MusA2 --------------RTKIPVSKVLVRRVSNRGLAGTTIRAAACHDSAKKL--
HuA2 --------------RTKIPVSKVLVRRVSNRGLAGTTIRATACHDSAKKL--
ChimpA2 --------------RTKIPVSKVLVRRVSNRGLAGTTIRATACHDSAKKL--
RatA2 --------------RTKIPVSKVLVRRVSNRGLAGTTIRAAACHDSAKKL--
ArmA2 --------------RTKIPVSKVLVRKVSSRGLAGTTLRAAACHDSAKKL--
OpoA2 --------------RTKIPVSKVLVRRVSNRGLAGTTIRAAACHDGAKKL--
ChickA2 --------------RTKIPVSKVLVRRVSNRGLAGTTVKAATHQDSAKK---
XeTA2 --------------RTKIPVSKVLVRRVSNKAITETAAGKRAIHDPARKHH---
XeLA2 --------------RTKIPVSKVLVRRVSNKAITETAAGKRAIHDPARKHH---
ZFA2 --------------RTKIPVSKVPVRRSGGKSTSTSQSRK--
HuA3 ---------------QPAAPPGSQGAPRAPTEKLGGREGLASDAGGATVCSAPSRQELWAHPGTTGLLAGESKALGGATQGTGTLSRDASREEET-RGHS
ChimpA3 ---------------QPAAPPGSQGAPRAPTEKLGGREGLASDAGGATVCSAPCRQELWAHPGTTGLLAGESKALGGATQGTGTLSRDASQEEET-RGHS
MusA3 ------------VLREPAAPPDPHRVLRGASVGVKGREDQAT------TCFPPSRQEPWAHSGTKNLLVRECEVLGEPARGSKTPSKDDSLEEGT-QDFS
RatA3 ------------VLREPAAPPDPHRVLRGTSADMKGREDQALDMGKDVQCFSPSRQVPWAHSGTKDLLIREREVQGEPARGINTPSKDGSLEEGT-QNVS
ArmA3 --
OpoA3 --------------REDSSPGPPDEGYKCASD--GGRSWMAMMPHPAAGGWQMYMPEKEMVSHERGTLGESPSATGESSKELPPHPSQLSTEKGKGQSSS
ChickA3 ------------KYNDSLITRSPSPEQKVSTESQQEKKSEEESNSSHLGVFLDEGKAATQTSSENVAERDSCTQTGNVAPQGRAGSHHQDSPTAPGAAET
XeTA3 ------------GENQETMRHELKSERQIAKDAFNHNENEDHDSKHQQELIGPQTEEAPVCTSETSRLEEKSGSLPSDNQSNYKPPLLFSNEDKYKNEVE
ZFA3 -------------KSNPSELKSSQTSGEEGGDLCLRCKSKSERVRRPPRRVRTVNSIRDAAKFKEPSQRGVRVLPSKCDTNTVMSSLDSPHSQPNTPTSG

```
                    1210      1220      1230      1240      1250      1260      1270      1280      1290      1300
              ....|....|....|....|....|....|....|....|....|....|....|....|....|....|....|....|....|....|....|....|
MusA1         -YYHKHAFNSYHSRFYQG--------LPWGVSSLPRYLGLPGVHPRPPPAAMALNRRSRSLDNAESLELELSSSHLAQGYMESDELQAHQEDSDEEGEEE
HuA1          -YYHKHAFNNYHSRFYQG--------LPWGVSSLPRYLGLPGLHPRPPPAAMALNRRSRSLDTAETLEMELSNSHLVQGYLESDELQAQQEDSDEEDEEE
ChimpA1       -YYHKHAFNNYHSRFYQG--------LPWGVSSLPRYLGLPGLHPRPPPAAMALNRRSRSLDTAETLEMELSNSHLVQGYLESDELQAQQEDSDEEDEEE
RatA1         -YYHKHAFNSYHSRFYQG--------LPWGVSSLPRYLGLPGVHPRPPPAAMALNRRSRSLDNAETLELELSSSHLAQGYVESDELQDHQEDSDEEGEEE
ArmA1         -YYHKHTFSNYRNRFYQG--------LPWGVSSLPRYLGLPGMHPRPPPAAMALNRRSRSLDTAETLELGLSNSHLSQGYVESDELQAEQEDSDEEEDEE
OpoA1         -YIPKETLDDLDERLFPGS----FRGLPWGVGSLPRHLGLPGLSPPPLPPPMTLNRRSRSLD-TETLELELAGSQLAPSYMESGKREEPEEGR-------
ChickA1       -YVQKESFAECDERMFLGYPQGSFQSCNWGVASLPRHLHLHGLSP-AMPAPLSLNRRSRSLD-TESLEFELADLQVSKNGLKPCQLWSKWDKKYSDGARR
XeTA1         -CVQES-LADCEELFHTDLPN-PLQTYNWGVTSLPRHFGHYKLNS-SMPAPLSSNRRSRSLD-TENLETELGALRLSKSGLKSYDFLGPREDGKNS----
ZFA1          CYLQQDAFAECDQRTFDAYEQSLLLSNAWGIASLPRHLSLGRPCP-PVPAPLALNRRSRSLD-TDSLEFQTSEIYTSVTKYDSKGTAFSQSRTVDCN---
MusA2         ----------------------------------------------------------------------------------------------------
HuA2          ----------------------------------------------------------------------------------------------------
ChimpA2       ----------------------------------------------------------------------------------------------------
RatA2         ----------------------------------------------------------------------------------------------------
ArmA2         ----------------------------------------------------------------------------------------------------
OpoA2         ----------------------------------------------------------------------------------------------------
ChickA2       ----------------------------------------------------------------------------------------------------
XeTA2         ----------------------------------------------------------------------------------------------------
XeLA2         ----------------------------------------------------------------------------------------------------
ZFA2          ----------------------------------------------------------------------------------------------------
HuA3          EGLFSSMESAATSTTDTSGKNKAPVPSTWPCSQKEPGPPGVLGCFRGPWRPGHGGDTLDAEPMLAGCVARVAALKISSNEQPPAAWPPRQDMGSGLFGQR
ChimpA3       EGLFSSMESAATSTTDTSGKNKAPVPSAWPCSQKEPGPPGVLGCFRGPWRPGHGGDTLDAEPMLAGCVARVAALKISSNDQPPAAWPPRQDMGSGLFGQR
MusA3         EGQSS---SEATMT-SISGNNKAVTSATCLSSQKELGTPGNLRYSQGPLRPGHRGSALDPGPMLVGCVTHVAALQIYPDSNS-----PRQDKGNGLFWKP
RatA3         EGRSS---SEVTTTTSISRNSKAVNTATRLSSQKELGTPGNLRCSQGSLRPGPGGSALDPGPMLVGCV---AALQIYPDSHS-----PRQDKGNGLLWKP
ArmA3         ----------------------------------------------------------------------------------------------------
OpoA3         EPSSLPIEMEEVTLTDALDEHRVLSPSLCPSTNEKPSQPLNLRCFRSQSSPGLKRGEPDIVPVLESCMAQVASLKISYENKPLAEWCLRKEEGSTLCKRQ
ChickA3       TGASN-----CVPQLQANGDDTQPSSAEDKATMTDRRQTSGGEDPLTESLSRHSGSQSSENPLLNDNHDAESCYSYKTAATSPPDPLEREDRSEAVYHPL
XeTA3         SYNCYFNKLDNTDSVNVFNSSNELQPP--KADNQNNIHSLDLECISSCMKPTMTENDQNIIKLLESCANQIASLHLSYKGHSNQQEKHINTEKIIQSQEV
ZFA3          VCFRIFNIGSPMTPGGDLQSPVVSSPGSGTRSLFVLAINKESLCESCKSSLKNGAKDLHLCRSCMSLIEHIKTSDLWARSSFPKSTLTPQPITQDLLSPA

                    1310      1320      1330      1340      1350      1360      1370      1380      1390      1400
              ....|....|....|....|....|....|....|....|....|....|....|....|....|....|....|....|....|....|....|....|
MusA1         E-GEWGRDSPLSLYTEPPGVYDWPPWAHCPLPVGPGL----AWMSPNQLYEPFNQSSYVQATCCVPP----VAMPVSVPGRTPG------DSVSQLARPS
HuA1          EEGEWSRDSPLSLYTEPPGAYDWPAWAPCPLPVGPGP----AWISPNQLDRPSSQSPYRQATCCIPP----MTMSISLSVPESR---APGESGPQLARPS
ChimpA1       EEGEWSRDSPLSLYTEPPGAYDWPAWAPCPLPVGPGP----AWISPNQLDRPSSQSPYRQATCCIPP----MTTSISLSVPESR---APGESGPQLARPS
RatA1         E-GEWGRDSPLSLYTEPPGVYDWPPWAHCPVPVGPGL----AWLSPNQLDEPFNQSSYVQATCCVPP----VAM--SVPGRAPG------DSVSQPARPS
ArmA1         ----WNRDSPLSLYTEPPGAYDWPTWAPCPFPVGPGP----TWVSPSKLDGPSIQFPYGQAACCLPP----MAMSMSLPVPGPKPN-AFQKTGPQLARPS
OpoA1         ----WGWESPLDSYVEPPETYDWPVWARCSPPTGLSH----GWQQPD-----VARTPFGSPACYGSL----RGLAPRDSWDRGT------QVRHQPVRPS
ChickA1       SRSKEEAELSAPEGGETNGVLSWPGLQHLQYDAELAPGGIKHWGFAPAAGMPSSWEPSERSDADPSF----FPLSRSCARETLERQPQDPEPNRLLVRPS
XeTA1         ----WRYENECFRAHET----EWGGCSFLEQSSQTSR----------------QFSQYTPTVSSNHG----YGRANEGIIPSKQ-----LPLNRQMAKTT
ZFA1          -------DMDFPRQPCRITVDSWR-------------------------------RGYRQNFDSSNA----SQQELKLPHLSQS-----------TVRPS
MusA2         ----------------------------------------------------------------------------------------------------
HuA2          ----------------------------------------------------------------------------------------------------
ChimpA2       ----------------------------------------------------------------------------------------------------
RatA2         ----------------------------------------------------------------------------------------------------
ArmA2         ----------------------------------------------------------------------------------------------------
OpoA2         ----------------------------------------------------------------------------------------------------
ChickA2       ----------------------------------------------------------------------------------------------------
XeTA2         ----------------------------------------------------------------------------------------------------
XeLA2         ----------------------------------------------------------------------------------------------------
ZFA2          ----------------------------------------------------------------------------------------------------
HuA3          WARGPDMLEQKQSSSSPSMTTIHGLPYSASTQD-QRCRDRVQDLS-------------------------------------------------------
ChimpA3       WARGPDMLEKKQSSSSPSMTTIHGLPYSASTQD-QRCRDRVQDLS-------------------------------------------------------
MusA3         QAWGPNILQKNPISSKPNEAAGCGLSSSASPQD-QKCRDLFLDLN-------------------------------------------------------
RatA3         QAWGPNAVLKKPTSSKRNEAAACGLSSSASPQD-QRCHDLFLDLN-------------------------------------------------------
ArmA3         ----------------------------------------------------------------------------------------------------
OpoA3         KVGSHCVLLQNECNRNPPNSGYPFLSPTGKLQE-TSFLNRTSQVSSN-----------------------------------------------------
ChickA3       SICDELLQPLGLGHFQGYISPTPRESSAGIVQLLEHCMSRVASLK-------------------------------------------------------
XeTA3         SNNIKNYIEQHKHGLQPTIYPALSCRKETKETNKETAISQLINCKEDSKDNHTSQMFTEQMRTPVFLSRPTFLPYFCNNFSPLISRCSSALYKMHNSREA
ZFA3          STCGIGSDISIASLVEQCASQFSSMKINMTQAHPREIRDAVVPEQVVKRNKDHSQKYLKSTHKRRP-----------------------VAATEKGLHAR
```

```
                   1410      1420      1430      1440      1450      1460      1470      1480      1490      1500
           ....|....|....|....|....|....|....|....|....|....|....|....|....|....|....|....|....|....|....|....|
MusA1      HLPLPMGPCYNLQSQ-----ASQSGRAKPRDVLLPVDEPSCSSISG---ANSQSQAKPVGITHGIPQLPRV----RPEPFQLQPNHYRASNLDLSKERGE
HuA1       HLHLPMGPCYNLQPQ-----ASQSMRARPRDVLLPVDEPSCSSSSGGFSPSPLPQAKPVGITHGIPQLPRV----RPEHPQPQPTHYGPSSLDLSKERAE
ChimpA1    HLHLPMGPCYILQPQ-----ASQSMRARPRDVLLPVDEPSCSSSSGGFSPSPLPQAKPVGITHGIPQLPRV----RPEHPQPQPTHYGPSSLDLSKERAE
RatA1      HLPLPMGPCYNLQSQ-----ASQSGRARPRDVLLPVDEPSCSSISG---ANSLSQAKPVGITHGIPQLPRV----RPEPLQLQPNHYRASNLDLSKERGE
ArmA1      HLPLPMGPCYNPQPQ-----ASQNVRARPQDVLLPVNEPSCSSSSGGFIPSPLPQTKPVGITHGNHQLPRV----QPETPQLQSIHYKVSDLDLSKERAE
OpoA1      HLDLQGGPCWNPQAQ-----YAQAETKKQAFMMSLESKRPIKSLSP----KLPVKCKSVGIIRGAPRSPQS----WEEQPKWHHVHSRGTNTNMLKGRDN
ChickA1    NLPLQPDTRQPQEASGSYRYHGEAPAKKLARVLPLGEPEPPQSISFAQSPEKPAKCKPVGVTQGMPQCHDSDDTETLKSSACFAERYGSTSKELLKARAA
XeTA1      DPPLQCNTRQSQEASTMCGYPAEQTTKKLALVLPLEDRRGVGIHQS--SYKSHTSGKPIGVTQAMPQQNKN--------EDDFIKPPADYQDNLLKRTVE
ZFA1       HLPLKNNCRSRNLPAATRVDGEGEILFGGGDALYPCSYPPMG---------TQWKNRPVGVTQGVPHLRSE--------------QSADHQKKKKKKKK-
MusA2      ----------------------------------------------------------------------------------------------------
HuA2       ----------------------------------------------------------------------------------------------------
ChimpA2    ----------------------------------------------------------------------------------------------------
RatA2      ----------------------------------------------------------------------------------------------------
ArmA2      ----------------------------------------------------------------------------------------------------
OpoA2      ----------------------------------------------------------------------------------------------------
ChickA2    ----------------------------------------------------------------------------------------------------
XeTA2      ----------------------------------------------------------------------------------------------------
XeLA2      ----------------------------------------------------------------------------------------------------
ZFA2       ----------------------------------------------------------------------------------------------------
HuA3       WLRVEPTGLGVQAWASVEDQPLQLST-----------EAVEQVAHGSQLDSEPRSAPAARWSSQGHHPESLGLTLNSQQEGGVSASAPECRCSLLAREGL
ChimpA3    WLRVEPTGLGVQAWASVEDQPLQLST-----------EAVEQVAHGSQLDSEPRSAPAARWSSQGHHPESLGLTLNSQQEGGVSASAPECRCSLLAREGL
MusA3      QLKLEPSRLGPQACSSVDSQPQQLCP-----------RAPEQVPHRGSVGS-------------------------------------------------
RatA3      QLTLEPSRLGTQACASVDSLPQQLCP-----------RAPEQVPHRGSVGS-------------------------------------------------
ArmA3      ----------------------------------------------------------------------------------------------------
OpoA3      KINFEYAQLNGQALSYIQDLHLQLSPGDPSV--ATQEVQPKFIPLFASSDSVGPGAFPQASCAGSSSPQNLCPCSDIKIPGPTSGSPLECHHNLLSRVGA
ChickA3    -ISYDNKHLEDNCIG--NDMNGIIWT------------MYSSVTFIGPYESHAKAEAQPSAQCSSLFTS-------------------------------
XeTA3      SIFLNTPIKCDKSLGNTEEYSEDANSTSSLLGSAEMSKRTESLTPTAYVDGKGQGQPPDKRRLTTLCVKKCLVVPVPRSNESSQVQAHAVDIQHLTQNLG
ZFA3       HRLSRSSFSEDVKRSLLDAGGLGFVSTSPSNDLVLETYTPCTVESVDTDVSQATRPTSLPLVASSEFSCREGLMKIEGGNSAKKTNKSRHRKKSAVNHEG

                   1510      1520
           ....|....|....|....|..
MusA1      QGASLSTSYSSTAMNGNLAK--
HuA1       QGASLATSYSSTAMNGNLAK--
ChimpA1    QGASLATSYSSTAMNGNLAK--
RatA1      QGASLSTSYSSTAMNGNLAK--
ArmA1      QRASLP----STAMNGHLAE--
OpoA1      HGLSFPPSYPSSAMNVSTGK--
ChickA1    PGNVLPSGCLSTAVNVMGAK--
XeTA1      QEVSANKKWANNYTECTGS---
ZFA1       ----------------------
MusA2      ----------------------
HuA2       ----------------------
ChimpA2    ----------------------
RatA2      ----------------------
ArmA2      ----------------------
OpoA2      ----------------------
ChickA2    ----------------------
XeTA2      ----------------------
XeLA2      ----------------------
ZFA2       ----------------------
HuA3       LCGQPEVGASGPAMAEPHL---
ChimpA3    LCGQPEVGASGPAMAEPHL---
MusA3      ----------------------
RatA3      ----------------------
ArmA3      ----------------------
OpoA3      LPCHAEVMAASPAMAETNL---
ChickA3    ----------------------
XeTA3      FAGRYRLLVPAPGRIQCFQ---
ZFA3       SCSGFPGDRKVERRSRMKKGNV
```

Figure SM7. Alignment of the 28 Amer1/WTX, Amer2, Amer3 proteins used for the construction of the Bayesian phylogenetic tree (Figure 3) and for the Maximum Likelihood tree. Amer proteins are named in the left margin using abbreviations of species (see additional file 2, Table 1). Proteins were aligned with the multiple sequence alignment program Clustal W and the alignment was manually refined where necessary.
