# Supplementary material for: The WTX/AMER1 gene family: evolution, signature and function
Source: BMC Evol Biol. 2010 Sep 15;10:280. doi: 10.1186/1471-2148-10-280 (PMC2949870; doi:10.1186/1471-2148-10-280)
Supplement: Additional file 5 — Additional figure SM8. Figure SM8 contains multiple alignment performed with a broader array of Wtx/Amer1, Amer2 and Amer3 proteins (73 sequences in total). [file 1471-2148-10-280-S5.PDF]

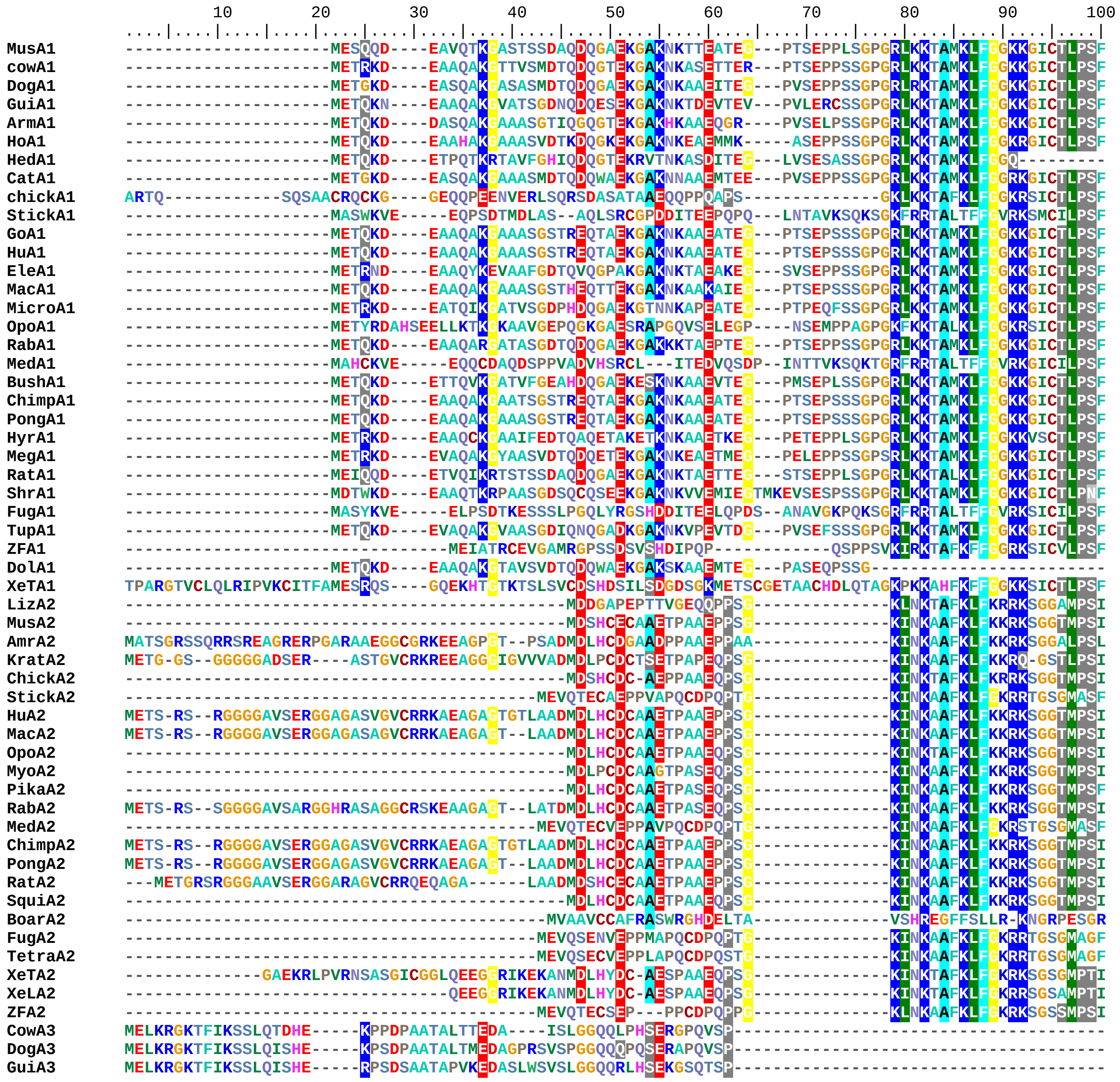

```
ZFA3      MELSKSEDSSDNRKRSNEA-------HGRSKQPDEHTSKITMNNGFVNEHNTDESPSSLPKPD-------------------------------------
ArmA3     -----------------QD-----KPPDP-ATTLAREGPGPWPSLP-GQPKPPGEKGPPSSP--------------------------------------
HoA3      MELKRGKTFIKSSLQISHE-----KPLDPAATVLAREDAGPWSVSPEGQQQPHGEKGPQVSP--------------------------------------
GoA3      MELKRGKTFIKSSLQVSHE-----KPPDPAAVAAAREGTGPWSVLPGGQQRPHSEKGPQASP--------------------------------------
MacA3     MELKRGKTFIKSSLQVSHE-----KPPDPAAVAPAREGAGPWSVLPGGQQRPQSEKDPQASP--------------------------------------
OpoA3     MELRRGKTFIKSSLQISHE-----KPTVAEAALRGKETGPFRPNPTGTDGTSLEEESQKVGL--------------------------------------
musA3     MELRRGKTFIKSSVQISHE-----KLIDS----PAKEDPDKWPLSLGEQQRAYGEKSSQTSP--------------------------------------
PlaA3     MELRRGRTFIKSSLQLSQE-----KPSAVDSAARGQEGATVAGPSSAGDGQLSGEEGPRASS--------------------------------------
RabA3     MELKRGKTFIKSSLQISHE-----RAPEPAATAPAREDAG------------SSEKGSHTRP--------------------------------------
ChimpA3   MELKRGKTFIKSSLQVSHE-----KPPDPAAVAAAREGTGPWSVLPGGQQRPHSEKGPQASP--------------------------------------
PongA3    MELKRGKTFIKSSLQVSHE-----KPPDPAAVAAAREGTGPWSVLPGGQQRPHSEKGPQASP--------------------------------------
megA3     MELKRGKTFIKSSLQTSHE-----KPPGPAATAPVREDTGPWSVLPGGQQQSHSEKGPQVSS--------------------------------------
ratA3     MELRRGKTFIKSSVQISHE-----KLIDS----PAKEDPDTWSLSLGEQQRAHGERSSQTSP--------------------------------------
DolA3     MELKRGKTFIKSSLQIDHE-----KPPDAAATAPATEDA----VSPGGQQRPHSERGPQVSP--------------------------------------
HuA3      MELKRGKTFIKSSLQVSHE-----KPPDPAAVAAAREGTGPWSVLPGGQQRPHSEKGPQASP--------------------------------------
chickA3   MELKRGKTFIKSSAQHERV-----PPAHTAPANRDEMGKDKR---AALEGNQSVAQVQLACL-----------ATHKNYRFSSRAARSGAGENS------
XeTA3     MELLRGKTFIKSYTQNPPEN----LPCIGVADKKIRDVERSISRLDKTNGFSIRETSNGKCV-----------NTTDNDRCN------------------

                   110       120       130       140       150       160       170       180       190       200
          ....|....|....|....|....|....|....|....|....|....|....|....|....|....|....|....|....|....|....|....|
MusA1     FGGGRSKGSGKVSSKKS-----------------LNKSKTHDGLSEASQGPEDV----VIEETDLSTPLSKSS-----------AQFPSSQSANGALEIG
cowA1     FGGGRSKGSGKGSSKKG-----------------LSKSKTHDGLSEAVHDPEDI----VSEGTGLSLPLPESS-----------CQLPSSQSVHGSLETD
DogA1     FGGGRSKGSGKSISRKG-----------------LSKSKTHDGLTEAAHGPKDI----FSEGTSFALPLPESS-----------CQLPSSQSAHGVLETD
GuiA1     FGGGRSKGSGKSVSKKG-----------------LRKSKTRDGLSEVDCGSKDV----VSEETDFSLPFPESP-----------SPFPSSPCTHGALEIG
ArmA1     LGGGRNKGSGKGNSKKG-----------------FSKSKTHDGLSETACDSEDV----ASEGTDLLLPLPDSS-----------CQLPSSQSAYGALETG
HoA1      FGGGRSKGSGKGSSKKS-----------------LSKSKTHDSLNEAACGPEDI----VSERTSLSLPLPESP-----------CELPSSRSAHRALETG
HedA1     ---------------------------------------------------EDI----VVEGASLSLFLPHSP-----------CQLPNSQSEHGILEAD
CatA1     FGGGRSKGSGKGISKKG-----------------LSKSKTHDGLTEAAHGPEDS----VSEGTRFALPLPESP-----------CQLPSSQSAHGVLCR-
chickA1   FSS-RNKGQGKGASKKG-----------------LSKSKTHDGISGTAYDEGSGVQLESPSDGSR----DSHP-----------CLLPSSQSVHVAIDTS
StickA1   FGG-RSRNPSKWTSKKG-----------------IGKSSTHDGLSKVSHDDNLS-----------------RR-----------CTSARDCEYLGQKDSA
GoA1      FGGGRSKGSGKGSSKKG-----------------LSNSKTHDGLSEAAHGPEDV----VSEGTGFSLPLPELP-----------CQFPSSQSAHGALETG
HuA1      FGGGRSKGSGKGSSKKG-----------------LSKSKTHDGLSEAAHGPEDV----VSEGTGFSLPLPELP-----------CQFPSSQSAHGALETG
EleA1     FGGGRSKGSGKGSSRKG-----------------LGKSKTHDGLGEAVYGPEDV----ASEGTDLTLPLPESP-----------CKFSSSQSTHGSLETG
MacA1     FGGGRSKGSGKGSSKKG-----------------LSKSKTHDGLSEAAHGPEDV----VSEGTGFSLPLPELP-----------CRFPSSQSAHGALETG
MicroA1   FGGGRSKGSGKGSSKKG-----------------LSKSRTHDGLSEAACGPEDV----VSEGASFSLHLSESP-----------CRFPSSQSAHGALETG
OpoA1     LGGGRSKGPGKGAAKRG-----------------LSKSKTHDGLQEASQESEPVPL--SVKGANHNSPLAGSL-----------HLLPSSLSSHGTLEAG
RabA1     FGGGRSKGSGKGSSRKG-----------------LSKSKTHDGLSEAAHGPEEV----VSEGTGFPLPLPESP-----------CGFPSSQSAHGALETG
MedA1     FGG-RSKNQNKWHSKKG-----------------ISKSKTHDGLSNISHTDHLG-----------------SV-----------CALTEASEYHSQRETA
BushA1    FGGGRSKYSGKGSTKKV-----------------LNKSKTHEALSEAAHGSED-----VSEGTSFSLTLPELL-----------CQFPSSQSIHGVLERG
ChimpA1   FGGGRSKGSGKGSSKKG-----------------LSKSKTHDGLSEAAHGPEDV----VSEGTGFSLPLPELP-----------CQFPSSQSAHGALETG
PongA1    FGGGRSKGSGKGSSKKG-----------------LSKSKTHDGLSEAAHGPEDV----VSEGTGFSLPLPELL-----------CRFPSSQSAHGALETG
HyrA1     LGGGRSKGFGKGSSRKG-----------------LSKSKTHDGLSEAVCGPEDV----AIEGTEFTLPLRESP-----------CKFSSSQSIHAALETG
MegA1     FGGGRSKGSGKGSSKKG-----------------LSKSKTHDGLSEAAHSHEEI----VSEGPSFSLPLPESP-----------CQLPNSQSAHGALETD
RatA1     FGGGRSKGSGKGSSKKG-----------------LNKSKTHDGLSESSHGPEDV----VVEETELSTPLSKSP-----------SQFPSSQSAHGALEIG
ShrA1     FGGGRSIGSGKSHTKKG-----------------LSQSKTHVGLSEVDQGPEDI----VSEGTSLSIPLLQPP-----------CQLPSSRSTHETLEVG
FugA1     FGG-RSKNPIKWSSKKG-----------------LAKSKTHDGLSKFSSDD--R-----------------SG-----------YVSAGDFECSNQRDAV
TupA1     FGGGRSKSSGKGSSKKG-----------------LSKSKTHDGLSEASHGLEDV----DSEGIGFSLPLPESP-----------SQFPSSLSTHGALETG
ZFA1      FGG-RGRSQRKGSSKTG-----------------VTKSQTYDGVSRACWDD----------------------------------------LGRSSSEVA
DolA1     ------------------------------------------------------------------------------------------LSAHGSLEAD
XeTA1     FGG-KHKGLGKGNFRKG-----------------LSKSKTHDGISDVQIEDGKKFFSDSSNIKLHGLELTRVG-----------TCLTSSHSTDLNVTAP
LizA2     FGVRSSKGKGDGGGAGGERA--AVASPVPAPAGGLVRSKTHDGLAEVG--LEGAVKKEKEAEEAEAP--------------ASPPSSSPSSS---AAVGK
MusA2     FGVK-NKG--DGKSSGP---------------TGMVRSRTHDGLAEVL-VLEGSKKEEPPGGSDHSGARP-----IPGPPKPSGPGLGSLAS---SSVAK
AmrA2     FGVK-NKG--DGKSSGK---------------TGMVRSKTHDGLAEVV-VLEGGKKEDPSGGPQRGGGGGDRR--SPDPPRAA---VGSPAP---SAVAK
KratA2    FGVK-HKG--DGKSSGP---------------AGMVKSRTHDGLAEAVSVLESGRKEEPR-----GWGGRGRA--GLGAPKAPEGRGGSSPANHHSAVAK
ChickA2   FGVR-SKGG-EGKGASK---------------TGMVRSRTHDGLADAV--LESGKKEDAGGGEAQGK---------DAPSRAAGGLGGSASS----SVAK
StickA2   FSFR-NKGATNGGSNGNPDNGNSVNGNGSAASAELVRSKTHDGLTSSN-SDADGQRGEGLAI---LEAG-------------------PVR-----SLSK
HuA2      FGVK-NKG--DGKSSGP---------------TGLVRSRTHDGLAEVL-VLESGRKEEPRGGGDSGGGGGGRP--NPGPPRAAGPGGGSLAS---SSVAK
MacA2     FGVK-NKG--DGKSSGP---------------TGLVRSRTHDGLAEVL-VLESGRKEEPRGGGDGGGGGGGRP--NPGPPRAAGPGGGSLAS---SSVAK
```

```
OpoA2     FGVK-NKG--DGKSSSK---------------TGMVRSKTHDGLAEVL-MLESSKKEEPNSGGGSAGGGSGDRLNADTHPRAAVTSVSSLAN---SSVAK
MyoA2     FGVK-NKG--DGKGAGP---------------PGMVRSRTHDGLAEVV-VLEGGRKEEPRGGGDGGG---------------------------------
PikaA2    FGVK-NKG--DGKSSGP---------------AGMVRSRTHDGLAEPG----RGEPAEAS----------------------------------------
RabA2     FGVK-NKG--DGKSSGPT--------------TGMVRSRTHDGLAEVV-VLEGGRKEEPR--------GGARP--NPAPPRAAGGGGGSAAP---SSVAK
MedA2     FSFR-NKGATSSVNNGNSENGNSLS---LAASAELVRSKTHDGLTGSN-NDTDGQKGDALAA---LEAG-------------------PVR-----SLSK
ChimpA2   FGVK-NKG--DGKSSGP---------------TGLVRSRTHDGLAEVL-VLESGRKEEPRSGGDSGGGGGGRP--NPGPPRAAGPGGGSLAS---SSVAK
PongA2    FGVK-NKG--DGKSSGP---------------TGMVRSRTHDGLAEVL-VLESGRKEEPRGGGDSGGGGGGRP--NPGPPRAAGPGGGSLAS---SSVAK
RatA2     FGVK-NKG--DGKSSGP---------------AGMVRSRTHDGLAEVL-VLEGSKKEEPPGGSDHSGARP-----IPGPPKASGPGLGSLAS---SSVAK
SquiA2    FGVK-NKG--DGKSSCP---------------TGMLS---------------ASRRKRPE----------------------------------------
BoarA2    AEQA-DAG--PAAGRQK---------------RGLRG-----LLGGVR-WRRKDRRPKDAAPAGRAG---------------------------------
FugA2     FSFR-NKGSGN---NMNSDNGNSLNGNSSVTSVEIIRSKTCDGLMSSS-NDADGQR-EGLAS---LEAG-------------------PVR-----SLTK
TetraA2   FSFR-NKGSAN---NPNSDNGNSLNGNSSATSVEIIRSKTCDGLMSSN-SDADGQR-EGLAS---LEAG-------------------PVR-----SLSK
XeTA2     FGVK-NKG--DSKGTGK---------------IGMVRSKTLDGLADVV--LESNKKEEPCTEAGAGQLNPEKS------PKVLTINADVSSN---SSVAK
XeLA2     FGVK-NKG--DGKGTGN---------------IGMVRSKTLDGLADVV--LESNKKEEPCTNAGAGQLNTEKS------PKVVTINPDVSSD---SSVAK
ZFA2      FSVR-NKGEST--------------GKAAGKTLELVRSKTHDGLITDTPSELDSHRKEESASSDQLHAGTPDG-----------VSTAPLRS----SITK
CowA3     ---STQGYNRCSDREAQPDTNGGPAALCGTTFKLVRKSKNHDSV----------PRADRAA----------------TATGQLVGSASFPGTP------S
DogA3     ---STQGYDRYPSKGAEPEPEVGAAAFCGATFKLVRKSKTHESV----------PGAGRAS----------------TATGQLVGSTSFPGPS------S
GuiA3     ---SAQGHGRNPNKGAHPNPDGGPVALCEATFKLVRKSKTHDSV----------LGAGTAT----------------TTTGQLVGSTSFPGTP------S
ZFA3      -ILFPGVSDTLSEGHRHLCSS-------------VTRSKTHDCVRGLGLQQAK-NSEYKDG-------------TSWRHHQKLLTSVSFPGFE-----TS
ArmA3     ---SPQGHDSVPARGGQPGPEEGPAAP----FKLVWKNRMQDCG----------PGAGRGS----------------AAR-QLVGSASFPGPP------G
HoA3      ---STQGHDKCSNKGGQPDPEVGAAAFCGATFKLVRKSKTHDNV----------PEAGRAG----------------AATGQLVGSASFPGPS------G
GoA3      ---STQEYDRCPNKGAQLDPKGGPAALCGATFKPVRKSKTHDSM----------SGAGRAT----------------AATGQLVGSASFPGSP------G
MacA3     ---SAQEYDRCPNRGAQPDPKGGPAALCGATFKPVRKSKTHDSV----------SGAGRAM----------------AATGQLVGSASFPGSP------G
OpoA3     ---VSHKHYRFSNKVTRLAPDEDLGASCGSSYKLVRKSKTHDCV----------MGAEKP-------------------VGRLVNSISFSGLG-----SS
musA3     ---CSQGYGRCPNKEVLSDPEGGPVPLCGTTFKLVRKSKTHDSV----------PGAVKAA----------------APTGQMVGSTSFSETP------G
PlaA3     ---ATQKPYRFSNKAARPGAYENFATSPGSSYKLVRKSKTHDCM----------IDADKT-------------------EGRLVNSLSFSGVGN-----S
RabA3     ---TVHGSDGCPNKEVQSDSEAGPVAS-GATFKLVRKSKTHESV----------PGAGRAA----------------GTSGQLVGSASFPGPP------G
ChimpA3   ---SAQEYDRCPNKGAQLDPKGGPAALCGATFKPVRKSKTHDSM----------SGAGRAT----------------AATGQLVGSASFPGSP------G
PongA3    ---SAQEYDRCPNKGAQLDPKGGPTALCGATFKPVRKSKTHDSM----------SGAGRAT----------------AATGQLVGSASFPGSP------G
megA3     ---STQGYNRCSNKGVQPDPEEGATAFCGATFKLVRKSKTHNSV----------LETNRV-----------------VATGQLVGSTSFPELS------G
ratA3     ---CAQGYGRCPNKEVLSDPEGGPVPLCGNTFKLVRKSKTHDNV----------PGAVKAA----------------APTGQMVGSTSFTETP------G
DolA3     ---STQGYDRCSDKGAQPDANGGPAALCGTTFKLVQKSKTQDTV----------PRADRAA----------------AATGQLVGSASFPGPP------R
HuA3      ---SAQEYDRCPNKGAQLDPKGGPAALCGATFKPVRKCKTHDSM----------SGAGRAT----------------AATGQLVGSASFPGSP------G
chickA3   -----------------------LEKPSGTSYKLVRKSKTHDCV----------SEADKSEPCGPSSR-ACEEGFAAKGKGRLVNSVSFSGMS------S
XeTA3     --------------------------------KYVKKSKTHDCV----------TKEDKLEEIS-----------SASTKGRIPSSVSFSGFE------T

                  210       220       230       240       250       260       270       280       290       300
          ....|....|....|....|....|....|....|....|....|....|....|....|....|....|....|....|....|....|....|....|
MusA1     SKHKTSGTEAIEKAGVE---------KVPSVHKPKKSLKSFFSSIRRHRK-GKTSGADQSVPGAKELEGARTRSHEHVSSI-SLPSSEEIFRDTRKENAK
cowA1     SRCKRSVAGATEKAGAE---------KAHFVPKPKKGLKGFFSSIRRHRK-SKVSGAEQSNPGAKESEGARARPHEYVSSA-LLSHADEVLQAPRKENAK
DogA1     SRCKTSVAGATEKAEAE---------KALSLSKPKKGLKGFFSSIRRHRK-SKVSGAEKSELRAKGPEGARARPHEYVSSV-ILHHTEDILQAPRKENAK
GuiA1     YQHKTSVTGATKNTRAD---------KVLPIPKPKKGIKDFFNSIRRHRK-SRITRAEQSEPQAKLPEQDRVRSHEHVNPV-PLLCSKETFQALTNKNAK
ArmA1     SRYKMFVAGTTEKAGAE---------KVLSVAKPKKGLKGFFSSIRRYRK-SKVAETEQNEAGAKGPEGARTKSHEHVSSA-PLLHSDDTLQPPNKEDAK
HoA1      SRCKTSTAGATEKAGAE---------KAHSVPKPKKGLKGFFSSIRRHRK-SKVSRSEQHEPGANGPEGARTRPHEHVSPA-LLLPAEETLQAPRKQNAK
HedA1     SRNKLSLAVSTEKSGTE---------KSNSVPKPKKGLKGFFSSIRRHRK-SKVSGTEQNEPRAKKSEGARARTHEDITSA-FLPKADETHQPFKKENTK
CatA1     -----------EQAPKA---------KHLWLEQSS-----------------------------------------------------------------
chickA1   VKFDFGRQDGSPPGSIEGYEKKPNGDKS-SFPRPKKGLKGLLNSIRRHRK-SKVAECEKTELSEWPGDSEETSKAQGTKAETPGTVEEGVPGSVPLAAAC
StickA1   AGLHASCPNECSHSNADQ--------KSLTLPRQRRGLRSLFQSFKSHRQSHRNVGLDKTEMIAMSSPRCEKEVP-----VVQDNDNQYVTECLDSEPDV
GoA1      SRCKTSVAGATEKAVAE---------KVPSMPKPKKGLKGFFSSIRRHRK-SKVTGAEQSEPGAKGPERVRARPHEHVSSAPQVPCFEETFQAPRKENAN
HuA1      SRCKTSVAGATEKAVAE---------KFPSMPKPKKGLKGFFSSIRRHRK-SKVTGAEQSEPGAKGPERVRARPHEHVSSAPQVPCFEETFQAPRKENAN
EleA1     PRCKMSVPGAIEKAEAE---------KFPSVLKPKKVLKRLFSSIRRQRK-GKVTGTEQSEPGVKMSEGARARSHKHVSSD-PLLHSEETLQPSSKEDAK
MacA1     SRCKTSVAGATEKAVAE---------KVPSMPKPKKGLRGFFSSIRRHRK-SKVTGAEQSEPGAKGPERVKARPHEHVSSAPRVPCPEETFQAPRKENAN
MicroA1   SRCKTSVAGATEKAGAE---------KVPSVPKPKKGLKGFFSSIRRHRK-SKVVGAEQSDPGAQGPGEARARLHEHISSAPQVPHSEETFQSPIKENGK
OpoA1     TCLKKSPPGGPEKPRPE---------RVPTAPRPKKGLKGLFNSIRRHRK-PKVPEPERSSPGKEGLEVPKEGPGEPLSLA-PQPISRDECLPLLPSNAK
RabA1     SRRKTSASGAPEKAGDE---------KVPSMPKPKKGLKGFFSSIRRHRK-SKVTGAEQNEPGAKEPEGTKARPHELVSSSPLLPS-EESFQALRKENSK
MedA1     AELQSSCHNECSHSADQQ--------KSLTLGRHKRGLRTLFQSFKQSRG-QRNAGLDTTEMIAISFPQSGREGP-----AVQVS--RPVTECLGSEPDV
BushA1    FRYKISMAGVTEKAGAE---------KVPFLPKPKKGLKGFFSSIRRHRK-SKVAGPEQSDPEAKGPEEARGRPHEHVSSAPQVLHADEAFQASQKENIK
ChimpA1   SRCKTSVAGATEKAVAE---------KVPSMPKPKKGLKGFFSSIRRHRK-SKVTGAEQSEPGAKGPERVRARPHEHMSSAPQVPCFEETFQAPRKENAN
```

```
PongA1    PRCKTSVAGATEKAVAE---------KVPSMPKPKKGLKGFFSSIRRHRK-SKVTGAEQSEPGTKGPERVRARPHEHVTSAPQVPCFEETFQAPRKENAN
HyrA1     SRHKTSTPGAAEKAETE---------IIPSVLKPRKGLKGFFSSIRRQRK-GKITVMEQSEQGVKMSEGARPR--EHASPD-PLLHSEETLQSSGKEDAK
MegA1     FSSKASVAIATEKAETE---------KALSVPKPKKGLKGFFSSIRRYRK-VKISGAEQSKPGAKGPKGARARPHEHANSA-LLSHAEETLQAPRKEKAK
RatA1     SRHKTSVTEAVEKAGVE---------KVPSVHKPKKGLKGFLNSIRRHRK-SKVSGADQSGLGAKELEGTRTMSQEHVSSI-SLPSSEEIFRDAIKENTK
ShrA1     SRSKISMTRATEKSGAN---------KVPPVPKPKKGLKGFFSSIRRHRK-SKVSGAGQIEPDSKES-LVRVRPHEHVSSA-LLTHAEETSQVLKKENAK
FugA1     GELRPSCLSECSP-AADQ--------KSLTFTRQKKGLRGLFSSFKYHRN-HRNVGLEKTEMLAVPSSHCNKRVP-----VNQDSGDQYVTECLRSEPDV
TupA1     SRQKTSVTGTIEKAEAE---------KVPSASKTKKGLK-----------------------------------------------------APIKENAK
ZFA1      SGDFEFCSEPQKSQEDHG--------KSQSLPRQRRGLRGLFSSIRRHRK-NKNVEVEKREALEMSSSFHAKTVPGALPSVSDRGDYHGDSQGEELVPDV
DolA1     SRRKTSVAGATEKARAE---------KAPFVPKPKKGLKGFFSSIRRHRK-SKVSGAEQSDPRAKEPEGARDRPHEHVSSA-LLSHT-ENLQAPRKENAK
XeTA1     AQFDLSFHDTSPHSSTDCFDKKINGDKSLSFPRPKKGLKGLFSSIRRHKK-NKVPDVEKSEHFIHAAPCLTEQLNEPELENMQEGNEQTKIECLPTEVSQ
LizA2     SHSFFSLLRKNG----GGAKG------ERPKGRGGGGLKGLFNSMRWSRRETHKPVGGK----GEGGDGPGLMPGSLTASLECIKEEAPPPPKQPLPPSS
MusA2     SHSFFSLLKKNG--RSETGKG-DHAEASKAGGKQKRGLKGIFSSMRWHRRDKRGKEEEEK-AVRAAGPGNLVLPGSLTASLECVKEEPPRAARRPDSPGQ
AmrA2     SHSFFSLLKKNG--RSENGRG-EPAD--KAGGRQKRGLKGLFSGVRWPRKDKRAKED----RGAPAARAGLILPGSLTASL-------------------
KratA2    SHSFFSLLKKNGGGRPDSGRGGEPSDAGKAAGKQKKGLRGIFSSMRWHKRDKR--GEAA--AARAPR-GGLLLPGSLTASLECVKEETPRAS--------
ChickA2   SHSFFSLLRKNG--RPENGKAAENAEQ-RAGGRQKKGLKGIFSSMRWHRKDKIGKEERG---EASEIPSGLIMPGSLTASLECIKEETPKPLSETPNGAG
StickA2   SLSFFSLLRRGS--------FRSGENGGAGLVRRGRGLKGFFSSMRWRRK----EKTNEV---DAEEAERKAEKDGEVGDPERAKDITLTLEPPPHHHQV
HuA2      SHSFFSLLKKNG--RSENGKG-EPVDASKAGGKQKRGLRGLFSGMRWHRKDKRAKAEAA--EGRAPG-GGLILPGSLTASLECVKEETPRA---------
MacA2     SHSFFSLLKKNG--RSENGKG-DPADASKAGGKQKRGLRGLFSGMRWHRKDKRAKAEAA--EGRAAG-GGLILPGSLTASLECVKEETPRA---------
OpoA2     SHSFFSLLRKNG--RSENGKG-EHADQNKAGSKQKKGLKGIFSSMRWHKKDKHGKEEEKE-EPSE-IQSSLILPGSLTASLECIKEEIPKPLCEPENPTK
MyoA2     -------------------------------------------------KDKRGKEGGAR-GALG-GPAGLALPGSLTASLECVTEEAPRPAFASENPSR
PikaA2    ----------------------------QAGCKQKKGLRGLFSSMRWPRKDKRGKEEEEKKGARAAGAGGLILPGSLTASLECVKEATPGAAAAVREPLQ
RabA2     SHSFFSLLRKNG--RSEPGRG-DPAEASRAGGKQKRGLKGLFSSMRWPRKDKRGKEEEK--GARAAGAGGLILPGSLTASLECVKEATPRAA--------
MedA2     SLSFFSFLRRGS--------FRSGENGGVGIVRRGRGLKGFFSSMRWRRK----EKTSEG---ETEDVDSKNEKVGGIAESEKVKSITLTLEPPPHPHQN
ChimpA2   SHSFFSLLKKNG--RSENGKG-EPADASKAGGKQKRGLRGLFSGMRWHRKDKRAKAEAA--EGRAPG-GGLILPGSLTASLECVKEETPRA---------
PongA2    SHSFFSLLKKNG--RSENGKG-EPADPSKAGGKQKRGLR-LFSGMRWHRKDKRAKAEAA--EGRAPG-GGLILPGSLTASL-CVKEETPR----------
RatA2     SHSFFSLLKKNG--RSETGKG-DHAEASKAGGKQKRGLKGIFSSMRWHRRDKRGKEEEEK-AARAAGPGSLVLPGSLTASLECVKEEPPRTARRPDSPGQ
SquiA2    ----------------------------------------------------------------------------------------PRASRRPQ-PGR
BoarA2    ------------------------------------------------------------------APGELARPGSLTASLECVKEEAPAAAREPHRPGA
FugA2     SLSFFSLLRRGS--------FRSTESGGTGLVRRGRGLKGFFSSMRWRRK----DKTNDK---EVEELEGKRKEDGTSDDSE--KDITLTLEPPPHHHQE
TetraA2   SLSFFSLLRRGS--------FKSSEGGGSGLVRRGRGLKGFFSSMRWRRK----DKSNEK---EIEELVGKRKEDGKIDDSEKLKDVTLTLEPPPHHHHE
XeTA2     SHSFFSLLKKNG--KSENVRG--EQAEQKAGSRQKRGLKGLFNSMRWSKKDKSYKDDKE---GASESQPGLILPSSLTASLECIKEETQKPLCEKGKSTE
XeLA2     SHSFFSLLKRNG--KSENVKG--ELAEQKPGSRQKRGLKGLFNSMRWSKKDKSYKDDKE---GASENQPGLILPSSLTASLECIKEETQKPLCEKEKSEE
ZFA2      SFSFFSLLRRSS--------SRAG-DGTTTVGRRGRGLKGLFSSMRWRRKPQIQEDTLEV---AKEVKEGDLILSSSSGSVKTEKDMTLTLEPLPQVFEE
CowA3     SQRMIDYRHFVPQMPFVPAVAKSIPRKRISLKRPKKCFRNLFH---------------------------------------------------------
DogA3     SQRMIDYRHFVPQMPFVPAVAKSIPRKRISLKRPKKCFRNLFH---------------------------------------------------------
GuiA3     GPRMIDYRHFVPQMPFVPAVVKSIPKKRISLKRPKKCFRNLFH---------------------------------------------------------
ZFA3      SHEIDDYRNLTPQVPFVPCIGKSIPKRRRSLRKPRKAIKDLFV---------------------------------------------------------
ArmA3     SQRMIDYRHLAPQMPFVPAVAKSLPRKRISLKRPKKCLRNLFH---------------------------------------------------------
HoA3      SQRMIDYRHFVPQMPFVPAVAKSIPRKRISLKRPKKCFRNLFH---------------------------------------------------------
GoA3      SRRMIDYRHFVPQMPFVPAVAKSIPRKRISLKRPKKCFRNLFH---------------------------------------------------------
MacA3     SRRMIDYRHFVPQMPFVPAVAKSIPRKRISLKRPKKCFRNLFH---------------------------------------------------------
OpoA3     GQQMIDYRHFVPQMPFVPAVAKSIPRKRISLKRSKKGFRDIFH---------------------------------------------------------
musA3     GQRMIDYRHFVPQMPFVPAVAKSIPRKRISLKRSKKCFRNLFH---------------------------------------------------------
PlaA3     SQQMIDYRNFVPQMPFVPAVAKSFPRKRISLKRSKKGFRDIFH---------------------------------------------------------
RabA3     GQRMIDYRHFVPQVPFVPAVAKSIPRKRISLKRPKKCFWNLFH---------------------------------------------------------
ChimpA3   SRRMIDYRHFVPQMPFVPAVAKSIPRKRISLKTPKKCFRNLFH---------------------------------------------------------
PongA3    SRRMIDYRHFVPQMPFVPAVAKSIPRKRISLKRPKKCFRNLFH---------------------------------------------------------
megA3     NQRMIDYHHFVLQMLFVPAVAKSISRKRIFLKRPKKCFRNLFH---------------------------------------------------------
ratA3     GQRMIDYRHFVPQMPFVPAVAKSIPRKRISLKRSKKCFRNLFH---------------------------------------------------------
DolA3     SQRMIDYRHFVPQMPFVPAVAKSIPRKRISLKRPKKCFRNLFH---------------------------------------------------------
HuA3      SRRMIDYRHFVPQMPFVPAVAKSIPRKRISLKRPKKCFRNLFH---------------------------------------------------------
chickA3   S-QIIDYRNFVPQMPFVPAVAKTIPRKRISLKRSKKGLRDIFH---------------------------------------------------------
XeTA3     NQQMIDYRNFVPQMPFVPSVAKTLPRKRISLKKTKKGFRNIFQ---------------------------------------------------------

                  310       320       330       340       350       360       370       380       390       400
          ....|....|....|....|....|....|....|....|....|....|....|....|....|....|....|....|....|....|....|....|
MusA1     PQDAPGPKMSPAQVHFSPTTEKAACKNPE--KLTRTCASEFMQPKPVLEGGSLEEPHTSETEGKVVAGEVNPPNGPVGDQLSLLFGDVTSLKSFDSLTGC
cowA1     FQDVPGPNIPSISETSLAATEKAAFKDAE--KTLEAFVSAFLQPKPAPEASGPEEFHSPETGEKLVAGEVNPPNGPLGDQLSLLFGDVTSLKSFDSLTGC
```

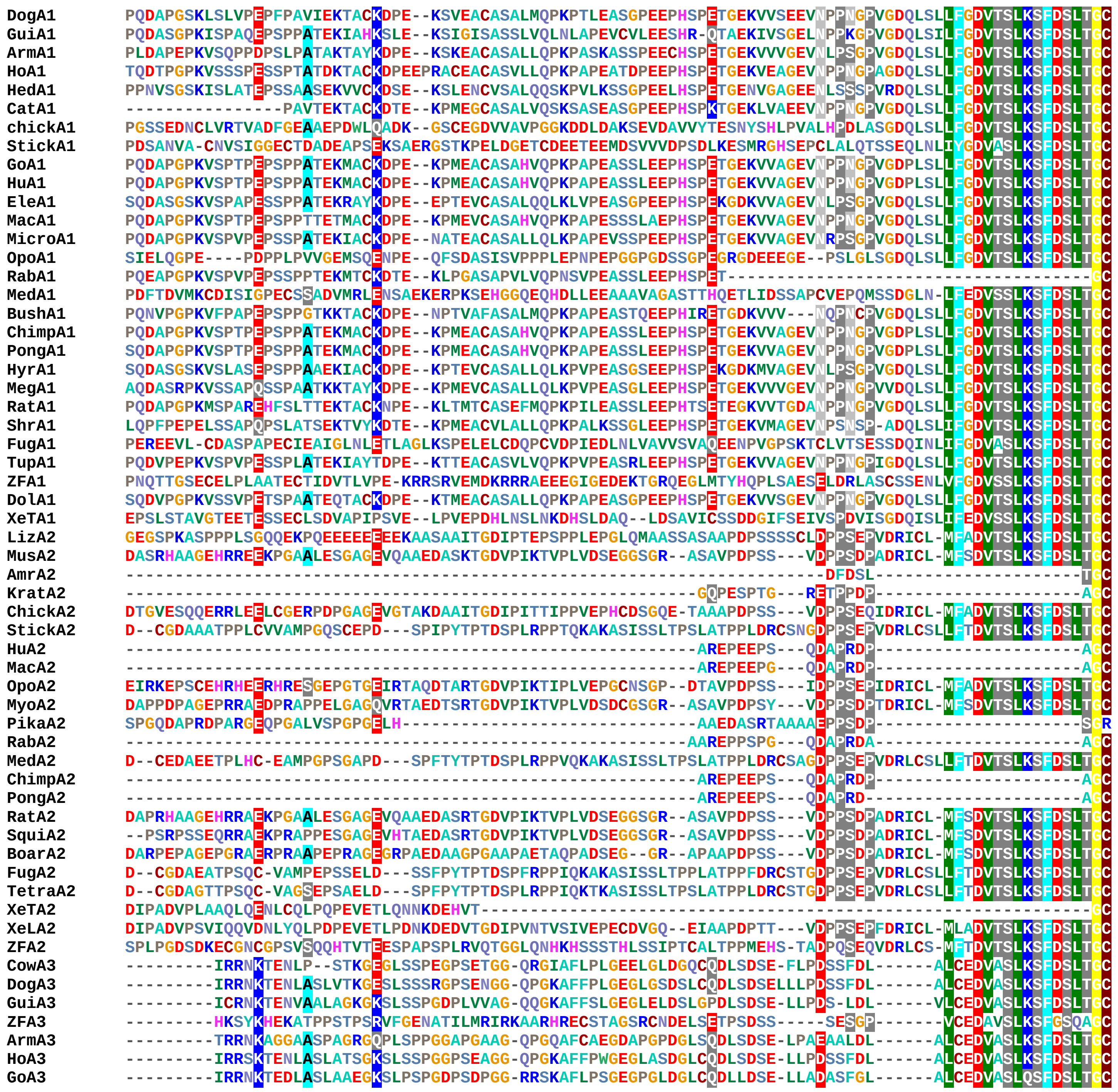
DogA1
GuiA1
ArmA1
HoA1
HedA1
CatA1
chickA1
StickA1
GoA1
HuA1
EleA1
MacA1
MicroA1
OpoA1
RabA1
MedA1
BushA1
ChimpA1
PongA1
HyrA1
MegA1
RatA1
ShrA1
FugA1
TupA1
ZFA1
DolA1
XeTA1
LizA2
MusA2
AmrA2
KratA2
ChickA2
StickA2
HuA2
MacA2
OpoA2
MyoA2
PikaA2
RabA2
MedA2
ChimpA2
PongA2
RatA2
SquiA2
BoarA2
FugA2
TetraA2
XeTA2
XeLA2
ZFA2
CowA3
DogA3
GuiA3
ZFA3
ArmA3
HoA3
GoA3

```
MacA3      ---------IRRNKTEELASLAAEGKGLPSPGDPSDPGG-RQSKAFLPPGEGPGLDSLCQDLSDSE-LLADASFGL------ALCEDVASLQSFDSLTGC
OpoA3      ---------IKKNKSENLAMLLGKGKNLPSPEGESEMSG-RLG----KPGETLAADSLAQDLSDSE-IQSDSSYDY------ALCEDVASLKSFDSLTGC
musA3      ---------MRRSKTENLASLSAKGKNLSPSG----VPA-QQGTAFLSMGEGLGLDSLCQDLSDSE-FLHDSPFDL------ALCEDVASLKSFDSLTGC
PlaA3      ---------MRKNKSEGLAFLVEKDRGPLSPGCQNDLLGGRQGKHPFKTGETFAADCLAQDLSDSE-LQSDSSYDY------ALCEDVASLKSFDSLTGC
RabA3      ---------IRRNKTENVAALVAKGKSLSSPGGLSRAAG-RRGTTLLPLGEDPGLDGLCHDLSDGD-LLLDSSFDL------ALCEDVASLKSFDSLTGC
ChimpA3    ---------IRRNKTEDLASLAAEGKSLPSPGDPSDPGG-RRSKAFLPPGEGPGLDGLCQDLLDSE-LLADASFGL------ALCEDVASLQSFDSLTGC
PongA3     ---------IRRNKTEDLASLAAEGKSLPSPGDPSDPGG-RRSKAFFPPGEGLGLDGLCQDLSDSE-LLADASFGL------ALCEDVASLQSFDSLTGC
megA3      ---------IRR-KTENLASLATNGKSLSSPWVSSDADG-RS-QVFFPG--ELGLDGLCQDLSDNE-LLPDSSFDL------ALCEDIASLKSFDSLTGC
ratA3      ---------MRRSKTENLASLSAKGKNLSPPG----VPA-QQGKPFFSMGEGLGLDSLCQDLSDSE-FLHDSPFDL------ALCEDVASLKSFDSLTGC
DolA3      ---------IRRNKTENLASLATKGKSLSSPEGPSEAGG-QQGTACFPLGEGLGLDSLCQDLSDSE-FLPDSSFDL------ALCEDVASLKSFDSLTGC
HuA3       ---------IRRNKTEDLASLAAEGKSLPSPGDPSDPGG-RRSKAFLPPGEGPGLDGLCQDLLDSE-LLADASFGL------ALCEDVASLQSFDSLTGC
chickA3    ---------IKKSKPESLTFLVEKDKNLSSSGCKGEVPG-RLTKSLFNTGETFAADCLSQDCSDNE-LQSDSSYDC------ALCEDVASLKSFDSLTGC
XeTA3      ---------LKRNKCQDIVSLTETNR-LQQILFNAEEGK-------MDSREIHPDELSVYEFSDNE--LNIDPIDR------RFCEDVASLKSFDSLTGC

                    410       420       430       440       450       460       470       480       490       500
               ....|....|....|....|....|....|....|....|....|....|....|....|....|....|....|....|....|....|....|....|

MusA1      GDIIAEQDMDSMT--------------DSMASGGQRANRDGTKRSSCLVTYQGGGEEMALPDDDDNEEEEEEEEEELLEDEEE--VKDGEENDD--LEYL
cowA1      GDIIAEQDMDSMT--------------DSMASGGQRANRDGTKRSSCLVTYQGGGEEMALPDDDD-EEEEEEE-VELEEGEE----VKEEEDDD--LEYL
DogA1      GDIIAEQDMDSMT--------------DSMASGGQRANRDGTKRSSCLVTYQGGGEEMALPDDEE-EEEEEDE-VELEEEEED---VKEEEDYD--LEYL
GuiA1      GDIIAEQDMDSMT--------------DSMASGGQRANRDGTKRSSCLVTYQGGGEEMALHD-------------------------------LEYL
ArmA1      GDIIAEQDMDSMT--------------DSMASGGQRANRDGTKRSSCLVTYQGGGEEMALPDDDDDEEEDEEEEVELEEEEEE--VK-EEEEDD--LEYL
HoA1       GDIIAEQDMDSMT--------------DSMASGGQRANRDGTKRSSCLVTYQGGGEEMALPDDDD-EEEEEEEDVELEEEEE----VKEEEDYD--LEYL
HedA1      GDIIAEQDMDSMT--------------DSMASGGQRANRDGTKRSSCLVTYQGGGEEMALPDDEE----EEEEEIELEEEEEEEEEAKEEEDND--LAYL
CatA1      GDIIAEQDMDSMT--------------DSMASGGQRANRDGTKRSSCLVTYQGGGEEMALPDDDD-EEEEEDEEVELEEEEED---VKEEEDYD--LEYL
chickA1    GDIIAEPDILSIA--------------ESTISVER--SRDTAKRSPCLVTYQGGGEEMAMS--------EEEYLQQIWDSTAE------------EGRS
StickA1    GDIIADQEDDSVT--------------ESSVSGER--SRSGGKRASCYLTYQGGGEEMASPEDLD---EECLHDFWATNASEDICCTYSQEHTDM-----
GoA1       GDIIAEQDMDSMT--------------DSMASGGQRANRDGTKRSSCLVTYQGGGEEMALPDDDDEEEEEEEEEVELEEEEEE--VKEEEEDDD--LEYL
HuA1       GDIIAEQDMDSMT--------------DSMASGGQRANRDGTKRSSCLVTYQGGGEEMALPDDDDEEEEEEEE-VELEEEEEE--VKEEEEDDD--LEYL
EleA1      GDIIAEQDMDSMT--------------DSMASGGQRANRDGTKRSSCLVTYQGGGEEMALPDDD---EEEEEEEVELEEEEEEE-VKGEEEDDD--LEYL
MacA1      GDIIAEQDMDSMT--------------DSMASGGQRANRDGTKRSSCLVTYQGGGEEMALPDDD--EEEEEEEEVELEEEEEE--VKEEEEDDD--LEYL
MicroA1    GDIIAEQDMDSMT--------------DSMASGGQRANRDGTKRSSCLVTYQGGGEEMALPDDEEEEEEEEEEEVELEEEEEE--VKEEQEEDD-DLEYM
OpoA1      GDIIAEQDVDSIT--------------ESIISAERGPGRESVKRSSCLVTYQGGGEEMAMQEEME------------EEEDEE------EETMG------
RabA1      GDIIAEQDMDSMT--------------DSMASGGQRANRDGTKRSSCLVTYQGGGEEMALPDDDDDDEEEEEE--ELEEEEEE--VKEEEEDND--LEYL
MedA1      GDITADQEDDSIT--------------DSTVSGER--SRNGGKRASCYLTYQGGGEEMASPEDFD---ETCLQEFWGN--NDAICCTCNPDQTD------
BushA1     GDIIAEQDMDSMT--------------DSMASGGQRANRDGTKRSSCLVTYQGGGEEMALPDDEE-----EEEEVELGEEEDE--AKEEEEEEDNDLEYI
ChimpA1    GDIIAEQDMDSMT--------------DSMASGGQRANRDGTKRSSCLVTYQGGGEEMALPDDDDEEEEEEEEEVELEEEEEE--VKEEEEDDD--LEYL
PongA1     GDIIAEQDMDSMT--------------DSMASGGQRANRDGTKRSSCLVTYQGGGEEMALPDDDDEEEEEEEEEVELEEEEEE--VKEEEEDDD--LEYL
HyrA1      GDVIAEQDMDSMT--------------DSMASGGQRANRDGTKRSSCLVTYQGGGEEMALPDE----EEEEEEEVELEGEEEE--VK-EEEEDD--LEHL
MegA1      GDIIAEQDMDSMT--------------DSMASGGQRANRDGTKRSSCLVTYQGGGEEMALPDDDD-EEEEEEEEVELEEEGEE---VKAEEDDD--LEYL
RatA1      GDIIAEQDMDSMT--------------DSMASGGQRANRDGTKRSSCLVTYQGGGEEMALPDDDDNEEEEEDEEVELEDEEEE--VKDGEEDDD--LEYL
ShrA1      GDIIAEQDMDSMT--------------DSMASGGQRANRDGTKRSSCLVTYQGGGEEMALPDDDD-----EEEEVELEVEEEV-----KEEDDD--LEYV
FugA1      GDIIADQEDDSIT--------------ESTVSGER--SRNGGKRASCYLTYQGGGEDMALPDDLA---EECLHDFWENDASEEMCYTCNQEHTEL-----
TupA1      GDIIAEQDIDSMT--------------DSMASGGQRANRDGTKRSSCLVTYQGGGEEMALPDDDDNEQDEEEEEVELEQ-EEE--LKEEEEDDD--LEYL
ZFA1       GDIIADQDDVSVA--------------ESSVSADRG-SRNAGKRSSCFVTYQGGGEEMATPDEID---ADYLQSLWESETSNEVCYIPSDRGSD------
DolA1      GDIIAEQDMDSMT--------------DSMASGGQRANRDGTKRSSCLVTYQGGGEEMALADDDD-EEEEEEE-VELEEEEEE---VKEEEDDD--LEYL
XeTA1      GDIIADQDIDTVS--------------DNAISLEQ--TRENAKRSSCLVTYQGGGEEMATPDDM-----EEEYLQQLLEDANE-------------AEGS
LizA2      GDIIADHEGDAGGGGGVSGGGGGSSSSEKSV-PGASKKPVS---VGGVLTYQGGGEEMASPEQVGDCAPEFWDVLSQTSRG-----------------
MusA2      GDIIAD-PEEEA---------GPS--CDKHV-PGPGKPVLS-KKNASVVAYQGGGEEMASPDQVDDYLPEFWDMLSQTEDQ----GQGTQEGAA--KAAT
AmrA2      GDIIAVLQEEEA---------GAS--GDKSA-PGPGKQVLS-QKTPGLLAYQGGGEEMASPDEVDDYLQEFWDMLSQTEGQ----GPGPREVAAK-AAAL
KratA2     GDIIAD-QEEEA---------DSS--CDKHM-PGSGQPALLSKKNPTVIAYQGGGEEMASPDGAGDYLQEFWDMLSQTEDPP---EARPQDRVAKGAMAQ
ChickA2    GDIIADQEEDVG---------GGSGGCEKST-PGAGKLGAP-KKHPTMVAYQGGGEEMASPDQVDDYLQEFWDMLSQTEET---------ETGGGGGGGG
StickA2    GDIIADAEEDGPGGN-----------------ARAPPPPQPHPAGSGVVAYMGGGEEMASPEGVDDDMQGLWHMLPSTGDNSPALPRSHQTPSTTPTSTY
HuA2       GDIIAD-QEEEA---------GPS--CDKHV-PGPGKPALS-KKNPGVVAYQGGGEEMASPDEVDDYLQEFWDMLSQTEEQ----GPEPQEGAAKVAAAL
MacA2      GDIIAD-PEEEA---------GPS--CDKHA-PGPGKPALS-TKNPGVVAYQGGGEEMASPDEVDDYLQEFWDMLSQTEEQ----APGPQEGAAKVAAAL
OpoA2      GDIIAD-QEDEA---------GAS--CDKNA-SGTSKPAPS-KKNPNMVAYQGGGEEMASPDEVDDHLQEFWDMLSQTEDQEAQEPQGVTAKAA--AGAA
MyoA2      GDIIAD-QEDEA---------GPS--CDKHA-PGTGKPGVS-KKNPSVVAYQGGGEEMASPDGVDDYLQEFWDMLSQTEDQ----GQGAPEGLA--KAAA
PikaA2     ALWRYNCRPGRRG--------GAS--CDRHG-PGPGKPVAS-KKTPGVVAYQGGGEEMASPDEADDCLQDFWDMLSQTEEQ----GQGPAEVEATTTAAP
RabA2      GDIIAD-QEDEA---------GPS--CDKHG-PGPGKPVLP-RKTPSVVAYQGGGEEMASPDQVDDYLQEFWDMLSQTEEP----APGAPEAAAKAAPAP
```

```
MedA2      GDIIADAEEEGPVGNGGSGTSSSSSGGGGGS-NKAPPPPQQHPAGSGVVAYMGGGEEMASPEGVDDDMQGLWHMLPSVGDDSPALPRVHQLSSTNPTSTC
ChimpA2    GDIIAD-QEEEA---------GPS--CDKHV-PGPGKPALS-KKNPGVVAYQGGGEEMASPDEVDDYLQEFWDMLSQTEEQ----GPEPQEGAAKVAAAL
PongA2     GDIIAD-QEEEA---------GPS--CDKHV-PGPGKPALS-KKNPGVVAYQGGGEEMASPDEVDDYLQEFWDMLSQTEEQ----GPEPQEGAAKVAAAL
RatA2      GDIIAD-PEEEA---------GPS--CDKHA-PGPGKPVLS-KKNPSVVAYQGGGEEMASPDEVDDYLPEFWDMLSQTEDQ----GQGTQEGAA--KAAT
SquiA2     GDIIAD-PEEEA---------GPS--CDKHA-PGPGKPVLS-KKNPSVVAYQGGGEEMASPDEVDDYLQEFWDMLSQTEEQ----GPGPQEGAA--KAAA
BoarA2     GDIIAD-PEDEA---------GPS--CAKHA-PGPGQPGPA-KKPPGVVAYQGGGEEMASPAEADDYVQEFWDMLSQTEDQ----GQGPQEGAATAAAAA
FugA2      GDIIADADDDGPSGNAGSGTSSSSSGGGGG--ARAPPSPEHHPPGSGVVAYMGGGEEMASPEGVDDDMQGLWHMLPSTGDNSPALPRSHQLPSSTQTSTY
TetraA2    GDIIADADDEGPSGNGGSGTSSSSSGGGGGGGARAPPSPQHHPPGSGVVAYMGGGEEMASPEGVDDDMQGLWHMLPSTGDNSPALPRSHQLPSSTPTSTY
XeTA2      GDVIAD-QDDDG---------GSSM-GSKLV-PGNGKKVMS-KKNTNIVAYQGGGEEMASPEQVDEYVQELFSMIPPSEGA----------------SEK
XeLA2      GDVIAD-QDDDG---------GSSK-GSKVV-PGNGKKVTS-KKNANIVAYQGGGEEMASPEEADEYVQELFSMIPQSEGA----------------SEK
ZFA2       GDIIADPEED--SGNGGSATSSGTGSSSGG--QRPRAAPK-KPQGSGVVAYMGGGEEMASPEGVDDDMQGLWHMLPQKDEDSPAPRRAEPV---------
CowA3      GEIFADESSVPSLELNEGL-------------ASPAQRSQASDSKTFRGPFQGSREQLASPAQNEMDFAKFWDHVNHSVRQQQ--------------HAL
DogA3      GEIFADESSVPSLELNEGL-------------ESPAQVSQALENKVLRGPFQGSVEQLASPAQNEMDFAKFWDSVNHSVRQQH--------------HAL
GuiA3      GEIFADESSVPSLELKDNL-------------KSPTQMSQASESKAPMGPS-WSMEQLASPAQKEVSFTKFWDSTTHSVQQQQ--------------HTL
ZFA3       GEIFADDLVSPDGVLN----------------QHDRVACGTPKQSPTTLGIQGGTECLASPANAEVDMFGLWETLNRTLLSEQ----------------S
ArmA3      GEIFADESSVPSLELGLGA-------------RSPTREDPALESGAPRGPLQGGVEQLASPAQNEADFATFWDSVNRSVRQQQQ------------QRAL
HoA3       GEIFADESSVPSLELNEGL-------------ESPAGASQALESKVPRGPFQGSVEQLASPAQNEMDFATFWDSVNQSVKQQQ--------------RVL
GoA3       GEVFADESSVPSLEL?EGP-------------ESPTQAAQGLESKVPRGPLQGSVEQLASPAQNEADFTRFWDSVNRSVRQQQ--------------RAL
MacA3      GEVFADDSSVPSLELNEGL-------------ESPTQAAQGLESKVPRGPLQGSVEQLASPAQNEADFTRFWDSVNRSVRQQQ--------------RAL
OpoA3      GEIFADEVSAS-LELEGSQ-------------EILTRRPRARENTSAMGSFQGGIEQLASPAQNEADFAKFWDNINWSVRRHQ--------------SAL
musA3      GEIFADGSSVPSVELKDGP-------------ESPAHSPQALDCKTPCGPAQGGMEQLMSPAQNEADFNKFWDSVNRSVQQQQ--------------RAL
PlaA3      GEIFADESSAP-LELEPSK-------------ESLAQRAKPKENSAPGGDLGKGRLEAAAAP--------------------------------------
RabA3      GEIFADESSVPSLELNDSL-------------ESPARASQAPESKTPGGPFQGGVEKLASPAQNEVDFTRFWDSVNRSVRQQH--------------RAL
ChimpA3    GEVFADESSVPSLELNEGP-------------ESPTQAAQGLESKVPRGPLQGSVEQLASPAQNEADFTRFWDSVNRSVRQQQ--------------RAL
PongA3     GEVFADESSVPSLELNEGP-------------ESPTQAAQGPESKVPRGPLQGSVEQLASPAQNEADFTRFWDSVNRSVRQQQ--------------RAL
megA3      GEIFADESSVPSLELNEGL-------------ESPARASQALDSKVPRDPFQG-----SMPAQNEMDFAKFWDSVNHSVRQQQ--------------HAL
ratA3      GEIFADGSSVPSVELKDGP-------------ESPAHSPQALDSKTPCGPSQGSMEQLMSPAQNEADFTKFWDSVNRSVQQQQ--------------RAL
DolA3      GEIFADESSVPSLELHEGL-------------ESPARASQAPDCMASRGPFQGSVEQLASPAQNEMDFDKFWDRVNHSVRQQQ--------------RAL
HuA3       GEVFADESSVPSLELNEGP-------------ESPTQAAQGLESKVPRGPLQGSVEQLASPAQNEADFTRFWDSVNRSVRQQQ--------------RAL
chickA3    GEIFADESSAH-LELESSK-------------EVLVRRSKHKE-SPVMGSFQGGVEQLASPGQNEAEFAKFWDNINKSVRLHQ--------------STL
XeTA3      GEIFADENHTFLGTDDK---------------KAEASLKCITKDRSMSGTFQGGVEKLASPAKAESDFTRLCGHNKSAKCFCS---------------NL

                   510       520       530       540       550       560       570       580       590       600
           ....|....|....|....|....|....|....|....|....|....|....|....|....|....|....|....|....|....|....|....|

MusA1      WASAQIYPRFNMNLGYHTAISPSH-QGYMLLDPVQSYPNLGLGELLTPQSDQQESAPNSDEGYYDSTTPGFEDDSGEALG-LAHRDCLPRDSYSGDALYE
cowA1      WASSQMYPRPILNPGYHPTTSPGH-LGYMLLDPVRSYPGSAPGELLTPQSDQQESAPNSDEGYYDSTTPGLEDDSGEALG-LVHRDCLPRDSYSGDALYE
DogA1      WASAQMYPKSNLNLSYHPITSPGH-HGYMLLDSVRSYPGLAPGELLTPQSDQQESAPNSDEGYYDSTTPGFEDDSGEALG-LIRRDCLPRDSYSGDALYE
GuiA1      WASAQMYPRPNINLDYHSTTSPGH-HGYRLLDSVQSYSGLASEELLTPQSDQQESSPNSDEGYYDSTTPGFEDDSGEALG-LVHRDCLPRDSYSGDALYE
ArmA1      WASAQMYPRPSLNLGYHSTTSPSH-HNYMLLDPVQSYPGLASGDLLTPQSDQQESAPNSDEGYYDSTTPGFEDDSGEALG-LIHKDCLPRDSYSGDALYE
HoA1       WASTQMYPRPNLNLGYYPTTSPGH-HSYILLDPVRSYPGLAPGDLLTPQSDQQESAPNSDEGYYDSNTPGFEDDSGEALG-LVRRDCLPRDSYSGDALYE
HedA1      WTNALIYPRPHLNLGYHSTTFSGHHHGYMLLDPGQSCPLLAVGNLLTPQSDQQESAPNSDEGYYDSTTPGFEDDSGETLG-LIHKDCLPRDSYSGDALYE
CatA1      SASAQMYPRPNLHLGYHPITSPGE-HGYMLLDSVRSYPGLAPGDLLTPQSDQQESAPNSDEGYYDSTTPGFEGDSGETLG-LVRRDCLPRDSYSGDALYE
chickA1    YEAPLPPVVSSPELQAVSSKLETRGLHEGEVHPYASGAVDG-VELLTPQSDQQESAPNSDEGYYDSTTPGPEDETGDGLC-EIKKDRLPRDSYSGDALYE
StickA1    --TADLTSSHNMDLLNSNSAQQGS---------GMDASSI--ADVLTPQSEHQESVPTSDEGYYDSTTPG-PEDGQDKAD-GLRAEKLPRDSYSGDALYE
GoA1       WETAQMYPRPNMNLGYHPTTSPGH-HGYMLLDPVRSYPGLAPGELLTPQSDQQESAPNSDEGYYDSTTPGFEDDSGEALG-LV?RDCLPRDSYSGDALYE
HuA1       WETAQMYPRPNMNLGYHPTTSPGH-HGYMLLDPVRSYPGLAPGELLTPQSDQQESAPNSDEGYYDSTTPGFEDDSGEALG-LVRRDCLPRDSYSGDALYE
EleA1      WASAQMYLRPNLNLSYHPTTSPGD-HSYMLLDPVRSYPGLASGDLLTPQSDQQESAPNSDEGYYDSITPGFEDDSGEALG-LVHRDCLPRDSYSGDALYE
MacA1      WATAQMYPRPNMNLGYHPTTSPGH-HGYMLLDPVRSYPGLAPGELLTPQSDQQESAPNSDEGYYDSTTPGFEDDSGEALG-LVHRDCLPRDSYSGDALYE
MicroA1    WASAQMYPRPNQNLGYHPTTSPSH-HGCMLLDTVRSYPGLAPGELLTPQSDQQESAPNSDEGYYDSTTPGFEDDSGEALG-LVHRDCLPRDSYSGDALYE
OpoA1      HLSERANSAREEAPIYHLTRELGP-HSCFLFEGAHSYSDPEPGELLTPQSDQQESAPNSDEGYYDSNTPGPDDDSGDGLG-PGKKDQLPRDSYSGDALYE
RabA1      WASAQMYPRPNMNLGYPPTTSPGH-HAYMLLDPVRSYPGLAPGELLTPQSDQQESAPNSDEGYYDSTTPGFEDDSGEALG-LVRRDCLPRDSYSGDALYE
MedA1      --TNELTSSHNLDSLNSSSAQQAV---------GIVVSST--ADALTPQSEHQESFPNSDEGYYDSTTPG-PQEGQEEGD-GLRAGRLPRDSYSGDALYE
BushA1     WASAKIYPRHNLNLSYHPTTSPGH-YGNMLLDPVRSYPSLSPGELLTPQSDQQESAPNSDEGYYDSTTPGFEDDSGEALG-LVLRDCLPRDSYSGDALYE
ChimpA1    WETAQMYPRPNMNLGYHPTTSPGH-HGHMLLDPVRSYPGLAPGELLTPQSDQQESAPNSDEGYYDSTTPGFEDDSGEALG-LVRRDCLPRDSYSGDALYE
PongA1     WETAQMYPRPNMNLGYHPTTSPGH-HGYMLLDPVRSYPGLAPGELLTPQSDQQESAPNSDEGYYDSTTPGFEDDSGEALG-LVRRDCLPRDSYSGDALYE
HyrA1      WASAQMYLRPSLNLGYHPTASPGH-HNYMLHDPVRSYPGLASGDLLTPQSDQQESAPNSDEGYYDSTTPGFEDDSGEALG-LAHKDCLPRDSYSGDALYE
MegA1      WASAQMYPRPNLNLGYHTTTSPGH-HSYMLFDPVQSYPGLAPGDLLTPQSDQQESAPNSDEGYYDSTTPGFEDDSGEALG-LVHRDCLPRDSYSGDALYE
RatA1      WASAQIYPRFNMNLGYHTAISPSH-QGYMLLDPVQSYPNLGLGELLTPQSDQQESAPNSDEGYYDSTTPGFEDDSGEALG-LVHRDCLPRDSYSGDALYE
```

```
ShrA1      WASAQMYSRSNLNLDYHSTTFPRH-PGYMVHDPVQFYPGLAPGDLLTPQSDQQDSAPNSDEGYYDSTTPGFEDDSGEALG-LVHRDCLPRDSYSGDALYE
FugA1      --TADLTISQNMNLLNSNSTQQPS---------GTDTSS---ADALTPQSDHQESVPNSDEGYYDSTTPG-PDEGQEKND-RLNNDRLPRDSYSGDALYE
TupA1      WVSAHMYLRPNLNLSYHPTTSLGH-QGYMLLDPVRSYPGQAAGEILTPQSDQQESAPNSDEGYYDSTTPGFEDDSGEALG-LVNRDCLPRDSYSGDALYE
ZFA1       --SPSLTPDQQLSSIRATSSSSPM---------GITETALTPADLLSPQSDRQESVPNSDEGYYDSTTPG-MEE--ESRE-RPHQERLPRDSYSGDALYE
DolA1      WTSSQVYPRPILNPGYHPTTSSGH-LGYMLLDPVRSYPGLGPGDLLTPQSDQQESAPNSDEGYYDSTTPGLEDDSGEALG-IVHRDCLPRDSYSGDALYE
XeTA1      YELNNE--KEATKVRTAFSQVETAVYPLCAQDSFTDRAILSNTELLTPQSDQQESAPNSDEGYYDSNTPGPEDDSGEGFN---HKERLPRDSYSGDALYE
LizA2      -KES---PKASKEVN-GAKGVQDGSASG----QHVGFNHSHKEDQKSRDKEQQEAIPSGDEGYWDSTTPGPEEDTTN----NIQKEVIPRDSYSGDALYD
MusA2      ASDIKLAPETSSDTR-CGEAAKDMSSVKRRRLHRIPIESQQKEEPKHPEKEHQEGVPNSDEGYWDSTTPGPEEESISNSS-SSKKVVIPRDSDSGDALCD
AmrA2      ETKA--GPEAPQDAR-GADAPRDASSVKRRRLHRIPTELQQKAEAKAREKEPQEGVPNSDEGYWDSTTPGPEEDGAG----SGQKAGLPRDSCSGDALYD
KratA2     EAKAS-VPETAKDAR-RAEAAKDASSVKRRRLNRIPVDPQAKEDPRHPEKEPQEGVPNSDEGYCDSTTPGPEEDRVN----SSKKARVPRDSDSGDALYD
ChickA2    GTKT---PEGLKENR-GTEGAQNRVAVKRGGLNQIPIHLNNKEEQKGREKEQHEGVPNSDEGYWDSTTPGPEEDSTT----SIQKETLPRDSYSGDALYD
StickA2    PPRATTNAAGCHLLSAHRSADRRLPPVKALGLSKIPVVGAAGGRAAKPPIPPGEKEPLSDEGYWDTPTATPDESGQQ----RNRKTALTRDSCSGDHLYD
HuA2       ETKV--VPETPKDTR-CVEAAKDASSVKRRRLNRIPIEPHPKEEPKHPEKEQQEGVPNSDEGYWDSTTPGPEEDSSS----SGKKAGIPRDSYSGDALYD
MacA2      EAKV--APETPKDTR-CVEAAKDASSVKRRRLNRIPIEPHPKEEPKHPEKEQQEGVPNSDEGYWDSTTPGPEEDSAS----SGKKAGIPRDSYSGDALYD
OpoA2      AKETKVAPESSKDGK-GKEGARDASLVKRSRIHRIPIELHHKEDLKHREKEQQEGIPNSDEGYWDSTTPGPEEDSTS----TIQKEGIPRDSYSGDALYD
MyoA2      ALDAKGAPETSKDTR-GVEVAKDVSSVKRRRLNRIPIESQPKEEPKHLQKEQQEGVPNSDEGYWDSTTPGPEEDGTG----SGKKAGISRDSYSGDALYD
PikaA2     EATSG-RSDTCPDAAMGTEAAKDVSSVKRRRLNRMPAEPHPKEEPRRPEKELEGGVPNGDQGSWDPTTPGPEGGSAG----GGPKAAVPRNSGGG-----
RabA2      ETKA--GPDTCPDARGGVDAAKDASSVKRRRLSRIPTELQPKEEAKRPEQEQQEGVPNSDEGYWDSTTRGSEGDGAG----SGQKAALPRDRYSGDALYD
MedA2      SSRANP----TFLSSGPGCSDRKAPQVKTLGLSKIPVVGAVGSRSAKPPLPPGEKEPLSDEGYWDTPTATPDESGLQ----RNQKLTLSRDSCSGDHLYD
ChimpA2    ETKV--VPETPKDTR-CVEAAKDASSVKRRRLNRIPIEPHPKEEPKHPEKEQQEGVPNSDEGYWDSTTPGPEEDSSS----SGKEAGIPRDSYSGDALYD
PongA2     ETKV--VPETPKDTR-CVEAAKDASSVKRRRLNRIPIEPHPKEEPKHPEKEQQEGVPNSDEGYWDSTTPGPEEDSSS----SGKKAGIPRDSYSGDALYD
RatA2      ASDTKLAPETSNDAR-CGEAAKDVSLVKRRRLHRIPIESQQKEEPKHPEKEHQEGVPNSDEGYWDSTTPGPEEVSVSNSS-SSKKVVIPRDSNSGDVLCD
SquiA2     ALETKVVPETSKDAR-SGEASKDVSLVKRRRLNRIPIESHPKEQPKHPEKEQPEGVPNSDEGYWDSTTPGPEEESAS----SGKKAGIPRDSYSGDALYD
BoarA2     APEAQVPPETPKDAR-CAEGAKEASSVKRRRVQRIPAELQPKEEPKQPDKEPQEGVPNSDEGYWDSTTPGPEEDGAR-------KVGLPRDSDSGDALYD
FugA2      PPHATSGLPSSYLPSGPRSVDRKLSQVKTLGISKIPVVGGAGTKAGKPPLPPGEKEPLRDEGYWDTPTATPDDNGLQ----QNQRTALSRDSCSGDHLYD
TetraA2    PPRATSGLPSSYLPSGPRSVDRKQSQVKTLGLSKIPVVGGATTKAGKTPLSPGEKEPLRDEGYWDTPTATPDDSGLQ----QNQRIGLSRDSCSGDHLYD
XeTA2      TEKVNGTTQAS-REVKCSDSAQDRNAIKPSKLKQVPVYRKERGDQNSKANEKRQCLRNSDEGYWDSPTPGQEEEEPR----SVGKQALARDSCSGDALYD
XeLA2      PEKVNGTTQVTAREVKCSDSGRDRNTVKPSKLRQVPIRRKERGDQNSKGNEKRQCHRNSDEGYWDSTTLGQEEEEPR----SVGKQALPRDSCSGDALYD
ZFA2       ------------LHHAPARLEKRPPQVKALGLSKIPVSGSS--KTGKQQPSELQDAPPSDEGYWDSPTPGPEDEDST----FLRRDGLLRDSCSGDALYD
CowA3      LGPWLGGPQASDTDQPRPDAARLAELPLCPCRDPHSSSKASSTDTGTPKSERPESVSTSDEGYYDSFSPGLEEDKKEAPSPGTPTAAFPRDSYSGDALYE
DogA3      LGPRL----GTDTDRPRLDAAGLAELPLCPCRDPYSGSKASSIDTGTPKSEQPESVSTSDEGYYDSFSPGLEEDKKEALSPGTRSAVFPRDSYSGDALYE
GuiA3      LGSWPAGPQGTDTAQPRPDMAELVKLPLFPYRGPPSGSQASSIDTGTPKSEQPESVSTSDEGYYDSFSPGLEE----ASGPTTPAVVFPRDSYSGDALYE
ZFA3       LKAMGPATKHTAPITISPSTTNSADITVSTPHPVEPEIKELNAKVMTPKSD--NQGNTSDEGYCDYVSPGYEDHSKSSFS-PVHSSKIPRDTYSGDALYE
ArmA3      LGRWLAGPPGPEADPPRPDAARLPELPLRPCGDPRGDSKASSVDTGTPKSEQPESVSTSDEGYYDSFSPGLEEDKREALSPGTPTSTFPRDSYSGDALFE
HoA3       LGPWLGVPQGTDTDQPRLDAAGLAELPLCPCKDPHSGSKASSVDTGTPKSEQPESLSTSDEGYYDSFSPGLEEDKKEALSPGMPAATFPRDSYSGDALYE
GoA3       LGPWLSGPQGTDRDQPRLDTAGLAELPLCPCRDPRSGSKASSIDTGTPKSEQPESVSTSDEGYYDSFSPGLEEDKKEAESPGTPAATFPRDSYSGDALYE
MacA3      LGPWLSGPQGTDRDQSRLDTAGLAELPLCPCRDPRSGSKASSIDTGTPKSEQPESVSTSDEGYYDSFSPGLEEDKKEAESPGTPAATFPRDSYSGDALYE
OpoA3      FDRKPEGSKPSDAGKPKYEPVMLADLPLSP-GGDPDSSKNSSIDTGTPKSEHQESTSTSDEGYYDSFSPGLEDDGKEAQSPGVPSGRFPRDSYSGDALYE
musA3      MGPWLTSPEGTETDQTRLDTSGLAELPLFPCRGPPSGSKASSIDTGTPKSEQPESVSTSDEGYYDSFSPGLEEEKKEAASPGTPAATFPRDSYSGDALYE
PlaA3      ---------------------MPSQPLSP-GRDPSSSKDSSIDTGTPKSEQQESTSTSDEGYYDSFSPGLEDEVKEAQTPSTP-GRFPRDSYSGDALYE
RabA3      LGPWLAGPQGTDTDQPKLDTAELAELPLCPCRDPPSGSKASSIDTGTPKSEQPESVSTSDEGYYDSFSPGLEEDKKEAPSPGTPAATFPRDSYSGDALYE
ChimpA3    LGPWLSGPQGTDRDQPRLDTAGLAELPLCPCRDPRSGSKASSIDTGTPKSEQPESVSTSDEGYYDSFSPGLEEDKKEAESPGTPAATFPRDSYSGDALYE
PongA3     LGPWLSGPQGTDRDQPRLDTAGLAELPLCPCRDPRSGSKASSIDTGTPKSEQPESVSTSDEGYYDSFSPGLEEDKKEAESPGTPAATFPRDSYSGDALYE
megA3      LGPWLGGPQVTDTAQPRLASAGLPELPLCPCRDAPSGSKASSIDTGTPKSEQPESVSTSDEGYYDAFSPGLEEDRKEALSPGTHAASFPRDSYSGDALYE
ratA3      LGPWLMSPQGTETDQPRLDSSGLAELPLFPCRGPPSGSKASSIDTGTPKSEQPESVSTSDEGYYDSFSPGLEEEKKEAASPATPAAAFPRDSYSGDALYE
DolA3      LGPWLGGPQGSDTDKPRPDAAGLAELPLCPCRDPHGGSKASSIDTGTPKSERPESVSTSDEGYYDSFSPGLEEDKKEAPSPGTPAAAFPRDSYSGDALYE
HuA3       LGPWLSGPQGTDRDQSRLDTAGLAELPLCPCRDPRSGSKASSIDTGTPKSEQPESVSTSDEGYYDSFSPGLEEDKKEAESPGTPAATFPRDSYSGDALYE
chickA3    FDKKVLKVPGSDRGKAGGQAAAPATSPQTPPDKDGDNSKESSVETGTPKSDNQESISTSDEGYYDSFSPGQDDEVKEAQTPGVP-GRFPRDSYSGDALYE
XeTA3      QVDLTLFPASQYTVAKEASHAPTIQQELESPSNDLVSSNGTFTDAESP-------VSTSDEGYCDSMSPGMFDEKKENAS----LPTYPRDSYSGDALFE

                   610       620       630       640       650       660       670       680
           ....|....|....|....|....|....|....|....|....|....|....|....|....|....|....|....|....|
MusA1      FYEPDDSLEHSPPGDDCLYDLRGRNSEMLDPFLNLEPFSS-RPPGAMETEEERLVTIQKQLLYWELRREQR---EAQEA-
cowA1      FYEPDDSLENSPPGDDCLYDLHCHSSEMFDPFLNFEPFSSPRPPGAMETEEERLVAIQKQLLYWELQREQ---REAREAC
DogA1      FYEPDDSLETSPPVDDCLYDLHGHSSEIFDPFLNFELFSSSRPPGAMETEEERLVTIQKQLLCWELRREQLEAREAQEAC
GuiA1      FYEPDDSLENSPPEDDCLYDLHGHSSKIFDPFLNFEPFSSSRPPGAMETEEERLVTIQKQLLYWELRREQL---EAWETC
ArmA1      FYEPVDSLENSPPGDDCLYDLHGCSSEMFDRFLDFEPFSSSRPPGAMETEEERLVTIQKQLLYWELRREQLEAREAWEAH
HoA1       FYEPDDSLENSPPGDDCLYDLHGHSSEIFDPFLNFETFSSSRPPGAMETEEERLVTIQKQLLYWELRREQLEAQEARETQ
```

```
HedA1     FYEPDDSLENSPPRDDCFYDLHGRNSEIFDSFWNFEPFSSSRPPGAMETEEERLVTIQKQLLYWELRREQLEAQQALEAR
CatA1     FYEPDDSLEASPPLDDCLYDLHGRSSEKFDPFLNFEPFSSSRPPGAMETEEERLVTIQKQLLYWELRREQLEVQEAQEAR
chickA1   FYEPDDTLMSPSHGEQSLFESKVSHPEIFSYFLDFCLPPEKSLIGVMETEEERLAAIQKELLFWELQREPG--LKRLDVP
StickA1   LFAPDESLISPHYESKSKLAGSKPCEYLSQPVDATDSAFVPDMNSREMYEVRDFLEIG----------------------
GoA1      FYEPDDSLENSPPGDDCLYDLHGRSSEMFDPFLNFEPFSSSRPPGAMETEEERLVTIQKQLLYWELRREQL---EAQEAR
HuA1      FYEPDDSLENSPPGDDCLYDLHGRSSEMFDPFLNFEPFLSSRPPGAMETEEERLVTIQKQLLYWELRREQL---EAQEAR
EleA1     FYELDDSLENSPPGDDCLYDLHGHSSEMFDPFLDFEPFSSSRPPGAMETEEERLVTIQKQLLYWELRREQLEAREA---H
MacA1     FYEPDDSLENSPPGDDCLYDLHGRSSEMFDPFLNFEPFSSSRPPGAMETEEERLVTIQKQLLYWELRREQL---EAQEAR
MicroA1   FYEPDDSLENSPPGDDCLYDLHGRSSEIFDPFLNFEPFSSFRPPGAMETEEERLVTIQKQLLYWELRREQL---EAWEAC
OpoA1     FFEPCDSLTGSPPGDEGLFDPPGPGPELFDNFLSFKPFPSSKGPGTMETEEERLVTIQRQLLYWELRRKRQ---------
RabA1     FYEPDDSLENSPPEDDCLYDLHGRSSEMFDPFLNFEPFSSSRPPGAMETEEERLVTIQKQLLYWELRREQL---EAREAR
MedA1     LFAPDESLISPHYENKSPHPNSKSSTFLSEPEDEMDAAFVPEMNGAELYAES-FLERPGTCSKSL---------------
BushA1    FYEPDDSLENSPPGDDCFYDLHGHSSGIFDPFLNFESLSSSRPPGAMETEEERLVTIQKQLLYWELCREQL---EAREAC
ChimpA1   FYEPDDSLENSPPGDDCLYDLHGRSSEMFVPFLNFEPFSSSRPPGAMETEEERLVTIQKQLLYWELRREQL---EAQEAR
PongA1    FYEPDDSLENSPPGDDCLYDLHGRSSEMFDPFLNFEPFSSSRPPGAMETEEERLVTIQKQLLYWELRREQL---EAQEAR
HyrA1     FYELDDSLENSPPGDDCLYDLHGHTSEMFDPFLDFEPFSSSRPPGAMETEEERLVTIQKQLLYWELRREQLEAQEAWETH
MegA1     FYEPDDSLENSPPGDDCLYDLHGRSSEIFDPFLNFEPFSSSRPPGAMETEEERLVTIQKQLLYWELRREQ---LEAQEA-
RatA1     FYEPDDSLEHSPPGDDCLYDLRGRNSEMLDPFLNLEPFSS-RPPGAMETEEERLVTIQKQLLYWELRREQR---EAQEA-
ShrA1     FYEPDNSLENSPSGDDCIYDLHGHSSEIFDPFT-FEPFSSSRPPGAMETEEERLVTIQKQLLYWELQREKFEAQEVWEAH
FugA1     LFVPDESLISPRYENKAALPGSNLCPYLNEAVGASDSAFVMDMNGAELYKIHHFLERPGACGQFK---------------
TupA1     FYEPDDNLENSPPEDDCLYDLHGHSSEMFDPFLNFEPFSSSRPPGAMETEEERLVTIQKQLLYWELRREQL---EAQEAR
ZFA1      LFEPDDRLLSPSLPPKD--AHSFVGAPLQADKSPTNPLYSLASTGAMETEEERLSKIQHALLCCE---------------
DolA1     FYEPDDSLENSPPGDDCLYDLHGHSSEMFDPFLNFEPFSSSRPPGAMETEEERLVTIQKQLLYWELRREQ---REAREAC
XeTA1     FYEPEDSLMSPAPGGESLYETKTPCSEIFDQFFDFSLTSNNDLICATETEEERLAVIQKQLLFWERQREAT--LKGMAHV
LizA2     LYTEPEEN--TATTT---SAEDVATGTCGKPLSPVTTTCPVKTHTTSLKDSKIPISIKHFTSPHSSHGPDT---------
MusA2     LYVEPEAS--PATLP---ATEDPPCLSRLKPVSPGTITCPLRTPGSLLKDSKIPISIKHLSNLPSSH-------------
AmrA2     LDAEPDDG--APGLP---ATEETSCLSRLKPVSPVTITCPLRTPGSLLKDSRIPISIKHLANLPASH-------------
KratA2    LYTEPNGS--PAAPP---ASEENTCLSRLKPVVPGTITCPLRTPGSLLKDSKIPISVKHLTNLPSSQ-------------
ChickA2   LYAEPDEN--PPGGP---PEEEVTCMPRSKPVSPITTTCSLKTPSSTVKDSKIPISIKHLASHPASHGTDT---------
StickA2   LYNDPEGAGESTPSPSSEYKLSPTSQTSPPSSSSSSFQSLKGSTSLPRDSKIP---------------------------
HuA2      LYADPDGS--PATLPGGKDNEETSSLSRLKPVSPGTITCPLRTPGSLLKDSKIPISIKHLTNLPSSH-------------
MacA2     LYADPDGS--PATLPGGKDNEETSCLSRLKPVSPGTITCPLRTPGSLLKDSKIPISIKHLTNLPSSH-------------
OpoA2     LYTDPEEN--PAGLP---SDEEVSGLSRSKPVSPITITCPLKTPGSLLKDSKIPISIKHLASLPASH-------------
MyoA2     LYADPDGS--PAALP---ANEEASCVSRLKPVSPGSITCPLRTPGSLLKDSKIPISVKHLANLPSSH-------------
PikaA2    ---DADAS--PAALP---AAEETTCTSRLKPVSPGAITCPLRTPGSLLKDSKIPISIKHLTNLPASH-------------
RabA2     LYADPDAS--PAAAP---ASEETSCASRLKPVSPGTITCPLRTPGSLLKDSKIPISIKHLANLPASH-------------
MedA2     LYNDPE---ESTPSPSTEHKVSPTSQTSPPSSSSSSFRSMKGSTSLPRESKIPVSTRQTSPPHSVSQSAL----------
ChimpA2   LYADPDGS--PATLPGGKDNEETSSLSRLKPVSPGTITCPLRTPGSLLKDSKIPISIKHLTNLPSSH-------------
PongA2    LYADPDGS--PATLPGGKDNEETSCLSRLKPVSPGTITCPLRTPGSLLKDSKIPISIKHLTNLPSSH-------------
RatA2     LYVEPEAS--PAALP---ATEDPPCLSRLKPVSPGTITCPLRTPGSLLKDSKIPISIKHLSNLPSSH-------------
SquiA2    LYADPDGS--PAVPP---ANEETSCLSRLKPVSPGTITCPLRTPGSLLKDSKIPISIKHLTNLPSSH-------------
BoarA2    LYAEPDGS--PAALP---AGEETSCLSRLKPVSPVTVTCPLRTPGSLLKDSKIPISIKHLANLPSSH-------------
FugA2     LYNEPEEEGESSPSPPSEYKLSPISPTSP-TSSSSSTFLSLKGSCSLPRESKIPISNRQSSPPHSTSQSAL---------
TetraA2   LYNDPEEEGESSLSPASECKLSPTSPTSP-TSS--STFLSMKGSCSLPRESKIPRSNRQSSPPHSTSQSAL---------
XeTA2     LYTDPDES--IAKAQ---VEEPPLSHSHSKPLSPVTTSCPVKT-ASSNKESKIPISIKHLPVHTTNQGTDS---------
XeLA2     LYTDPDES--IPKAQ---AEEPPVSHSHSKPLSPVTASCPVKT-ASSNKESKIPISIKHLPVHTTNQGIDS---------
ZFA2      LYDPDSPSAASPTKSAGDLKMNLPSPKCS--SSATSSFRSMKGSTSLPRDSKIPISVRQTPPSHSSSQGAL---------
CowA3     LFYDPAEGPGPSLDDDLCVSESPSGPAPGAPLSMCSFHVGAEEN-LAPVPGPDLLSQGFLQSSWRGKECLLK--------
DogA3     LFYDPSEGPVPSLDDDLCVSESLSGPALGAPLSMCSFHVGAEEN-LAPAPGPDVLSQSFLQSSWKGKECLLK--------
GuiA3     LFYDPSKGPVPSLDDELGVSGSLSGPALGTPLSMCSFHVGAEEN-LAPAPSPDLLSQGFLKSSWKGKECLLK--------
ZFA3      LFCDPSDAEIPICDDEIDLTDSIVGQCSDLPLSMYSFRVGAEEN-LAPSLARDFVGQELLESKWMGKDCLLK--------
ArmA3     LFCDPSESPAPGLDDDLCVSESLSGPALGTPLSMCSFCVGAEEN-LAPPPGPDLLSQS-LQSSWEGKECLL---------
HoA3      LFYDPREGPIPSLDDELCVSESLSGPALGAPLSMCSFHVGAEEN-LAPALGPDLLSQGFLQSSWKGKECLLK--------
GoA3      LFHDPSEGPVPSPDDDLCVSESLSGPALGTPLSICSFRVGAEEN-LAPAPGPDLLSQGFLQSSWKGKECLLK--------
MacA3     LFHDPSEGPVPSPDDDLCVSESLSGPALGTPLSMCSFRVGAEEN-LAPAPGPDLLSQGFLQSSWKGKECLLK--------
OpoA3     LFYDPSEAKIPILDDDLCVSENLSEQAVEIPLSIYSFHVGAEEN-LAPQPTLDIISQSFLHSTWKGKECLLK--------
musA3     LFYDPSEAPVPILDDDC-VSESLSGPALGTPLSMCSFRVGAEEN-LAPAPGPDLLSQGFLQSTWKGKECLLK--------
PlaA3     LFYDPGDAKIPILDDDLCVSESISEQAVNAPLSIYSFHVGAEEN-LAPRPPVDIISQGFLHSTWKGKECLLK--------
```

```
RabA3     LFYDPSESPVPSLDDDLCVSESLSGPALGTPLSMCSFHVGAEEN-LAPAPGPDLLSQGFLQSSWKGKECLLK--------
ChimpA3   LFHDPSEGPVPSPDDDLCVSESLSGPALGTPLSICSFRVGAEEN-LAPAPGPDLLSQGFLQSSWKGKECLLK--------
PongA3    LFHDPSEGPVPSPDDDLCVSESLSGPALGTPLSICSFRVGAEEN-LAPAPGPDLLSQGFLQSSWKGKECLLK--------
megA3     LFYDPSEGPIPSLEDDLCVSESLSGSALGAPLSMCSFHVGAEEN-LPLAPGPDLLSQSFLQSSWKGKECLLK--------
ratA3     LFYDPSEAPVPILDDDC-VSESLSGPALGTPLSMCSFRVGAEEN-LAPAPGPDLLSQGFLQSTWKGKECLLK--------
DolA3     LFYDPTEGPGPSLDDDLCVSESLSGPALGAPLSMCSFHVGAEEN-LAAAPGPDLLSQSFLQSSWRGKECLLK--------
HuA3      LFHDPSEGPLPSPDDDLCVSESLSGPALGTPLSICSFRVGAEEN-LAPAPGPDLLSQGFLQSSWKGKECLLK--------
chickA3   LFYDPNEVKIPVMDDDLCTSESVSEQAIEIPLSIYSFHVGAEEN-MASQPGIDIISQGFFHSTWKGKECLLK--------
XeTA3     LFYNSNETTPSALDSALSISGHSTD----NPQSMYSFCVGSEEN-MATHIASDLVEDFGLQGSWKGKECLMK--------
```

Figure SM8. Alignment of the 73 Amer1/WTX, Amer2, Amer3 proteins used for the construction of the Bayesian phylogenetic tree (additional file 3, Figure SM6) and for the Maximum Likelihood tree. Amer proteins are named in the left margin using abbreviations of species (additional file 2, Table 1). Proteins were aligned with the multiple sequence alignment program Clustal W and the alignment was manually refined where necessary.
